# A synopsis of the tribe Lachnophorini, with a new genus of Neotropical distribution and a revision of the Neotropical genus *Asklepia* Liebke, 1938 (Insecta, Coleoptera, Carabidae)

**DOI:** 10.3897/zookeys.430.8094

**Published:** 2014-08-01

**Authors:** Terry L. Erwin, Laura S. Zamorano

**Affiliations:** 1 Hyper-diversity Group, Department of Entomology, MRC-187, National Museum of Natural History, Smithsonian Institution, Washington, P.O. Box 37012, DC 20013-7012, USA; 2 Research Student, MRC-187, National Museum of Natural History, Smithsonian Institution, Washington, P.O. Box 37012, DC 20013-7012, USA; 3 Laboratorio de Zoología Acuática LAZOEA, Departamento de Ciencias Biológicas, Universidad de los Andes, Colombia

**Keywords:** Amazon Basin, Australia, Nearctic, Neotropics, Paleotropics, Eucaerina, Lachnophorina, Odacanthitae, Odacanthini, Calophaenini, new taxa, new distribution records, Cuenca Amazónica, Australia, Neárctico, Neotrópico, Paleotrópico, Eucaerina, Lachnophorina, Odacanthitae, Odacanthini, Calophaenini, nuevos taxones, nuevos reportes de distribución

## Abstract

This synopsis provides an identification key to the genera of Tribe Lachnophorini of the Western and Eastern Hemispheres including five genera previously misplaced in carabid classifications. The genus *Asklepia* Liebke, 1938 is revised with 23 new species added and four species reassigned from *Eucaerus* LeConte, 1853 to *Asklepia* Liebke, 1938. In addition, a new genus is added herein to the Tribe: *Peruphorticus* **gen. n.** with its type species *P. gulliveri* **sp. n.** from Perú. Five taxa previously assigned to other tribes have adult attributes that make them candidates for classification in the Lachnophorini: *Homethes* Newman, *Aeolodermus* Andrewes, *Stenocheila* Laporte de Castelnau, *Diplacanthogaster* Liebke, and *Selina* Motschulsky are now considered to belong to the Lachnophorini as genera *incertae sedis*. Three higher level groups are proposed to contain the 18 recognized genera: the Lachnophorina, Eucaerina, and *incertae sedis*.

Twenty-three new species of the genus *Asklepia* are described and four new combinations are presented. They are listed with their type localities as follows: (***geminata* species group)** *Asklepia geminata* (Bates, 1871), **comb. n**, Santarém, Rio Tapajós, Brazil; (***hilaris* species group)** *Asklepia campbellorum* Zamorano & Erwin, **sp. n.**, 20 km SW Manaus, Brazil, *Asklepia demiti* Erwin & Zamorano, **sp. n.**, circa Rio Demiti, Brazil, *Asklepia duofos* Zamorano & Erwin, **sp. n.**, 20 km SW Manaus, Brazil, *Asklepia hilaris* (Bates, 1871), **comb. n**, São Paulo de Olivença, Brazil, *Asklepia grammechrysea* Zamorano & Erwin, **sp. n.**, circa Pithecia, Cocha Shinguito, Perú, *Asklepia lebioides* (Bates, 1871), **comb. n**, Santarém, Rio Tapajós, Brazil, *Asklepia laetitia* Zamorano & Erwin, **sp. n.**, Leticia, Colombia, *Asklepia matomena* Zamorano & Erwin, **sp.n.**, 20 km SW Manaus, Brazil; (***pulchripennis* species group)** *Asklepia adisi* Erwin & Zamorano, **sp. n.**, Ilha de Marchantaria, Lago Camaleão, Brazil, *Asklepia asuncionensis* Erwin & Zamorano, **sp. n.**, Asunción, Río Paraguay, Paraguay, *Asklepia biolat* Erwin & Zamorano, **sp. n.**, BIOLAT Biological Station, Pakitza, Perú, *Asklepia bracheia* Zamorano & Erwin, **sp. n.**, circa Explornapo Camp, Río Napo, Cocha Shimagai, Perú, *Asklepia cuiabaensis* Erwin & Zamorano, **sp. n.**, Cuiabá, Brazil, *Asklepia ecuadoriana* Erwin & Zamorano, **sp. n.**, Limoncocha, Ecuador, *Asklepia kathleenae* Erwin & Zamorano, **sp. n.**, Belém, Brazil, *Asklepia macrops* Erwin & Zamorano, **sp. n.**, Concordia, Río Uruguay, Argentina, *Asklepia marchantaria* Erwin & Zamorano, **sp. n.**, Ilha de Marchantaria, Lago Camaleão, Brazil, *Asklepia marituba* Zamorano & Erwin, **sp. n.**, Marituba, Ananindeua, Brazil, *Asklepia paraguayensis* Zamorano & Erwin, **sp. n.**, San Lorenzo, Rio Paraguay, Paraguay, *Asklepia pakitza* Erwin & Zamorano, **sp. n.**, BIOLAT Biological Station, Pakitza, Perú, *Asklepia pulchripennis* (Bates, 1871), **comb. n**, Santarém, Rio Tapajós, Brazil, *Asklepia samiriaensis* Zamorano & Erwin, **sp. n.**, Boca del Río Samiria, Perú, *Asklepia stalametlitos* Zamorano & Erwin, **sp. n.**, Guayamer, Río Mamoré, Bolivia, *Asklepia strandi* Liebke, 1938, Guyana, *Asklepia surinamensis* Zamorano & Erwin, **sp. n.**, l’Hermitage, Surinam River, Surinam, *Asklepia vigilante* Erwin & Zamorano, **sp. n.**, Boca del Río Samiria, Perú. Images of adults of all 18 genera are provided.

## Abstract (es)

La presente sinopsis provee una clave dicotómica para todos los géneros del hemisferio oriental y occidental incluyendo cinco géneros anteriormente mal clasificados dentro de Carabidae. El género *Asklepia* Liebke, 1938 es revisado; 23 especies nuevas para la ciencia son incluídas, además de cuatro especies de *Eucaerus* LeConte, 1853, que son reasignadas dentro del género *Asklepia* Liebke, 1938. Adicionalmente, un nuevo género es asignado dentro de la tribu: *Peruphorticus* **gen. n.** y la especie tipo *P. gulliveri* **sp. n.** de Perú. Cinco taxones anteriormente asignados a otras tribus presentan atributos que los hacen buenos canditados para ser clasificados como Lachnophorini: *Homethes* Newman, *Aeolodermus* Andrewes, *Stenocheila* Laporte de Castelnau, *Diplacanthogaster* Liebke y *Selina* Motschulsky, son actualmente considerados como pertenecientes del complejo Lachnophorini como géneros *incertae sedis.* Se propone que los 18 géneros se distribuyen en tres taxones superiores: Lachnophorina, Eucaerina e *incertae sedis*.

Veinte y tres especies nuevas del género *Asklepia* son descritas y cuatro nuevas combinaciones son presentadas. Todas las especies y sus localidades tipo son enlistadas de la manera siguiente: (**grupo de especies *geminata*)** *Asklepia geminata* (Bates, 1871), **comb. n**, Santarém, Rio Tapajós, Brazil; (**grupo de especies *hilaris*)** *Asklepia campbellorum* Zamorano & Erwin, **sp. n.**, 20 km SW Manaus, Brazil, *Asklepia demiti* Erwin & Zamorano, **sp. n.**, circa Rio Demiti, Brazil, *Asklepia duofos* Zamorano & Erwin, **sp. n.**, 20 km SW Manaus, Brazil, *Asklepia hilaris* (Bates, 1871), **comb. n**, São Paulo de Olivença, Brazil, *Asklepia grammechrysea* Zamorano & Erwin, **sp. n.**, circa Pithecia, Cocha Shinguito, Perú, *Asklepia lebioides* (Bates, 1871), **comb. n**, Santarém, Rio Tapajós, Brazil, *Asklepia laetitia* Zamorano & Erwin, **sp. n.**, Leticia, Colombia, *Asklepia matomena* Zamorano & Erwin, **sp. nov.**, 20 km SW Manaus, Brazil; (**grupo de especies *pulchripennis*)** *Asklepia adisi* Erwin & Zamorano, **sp. n.**, Ilha de Marchantaria, Lago Camaleão, Brazil, *Asklepia asuncionensis* Erwin & Zamorano, **sp. n.**, Asunción, Río Paraguay, Paraguay, *Asklepia biolat* Erwin & Zamorano, **sp. n.**, BIOLAT Biological Station, Pakitza, Perú, *Asklepia bracheia* Zamorano & Erwin, **sp. n.**, circa Explornapo Camp, Río Napo, Cocha Shimagai, Perú, *Asklepia cuiabaensis* Erwin & Zamorano, **sp. n.**, Cuiabá, Brazil, *Asklepia ecuadoriana* Erwin & Zamorano, **sp. n.**, Limoncocha, Ecuador, *Asklepia kathleenae* Erwin & Zamorano, **sp. n.**, Belém, Brazil, *Asklepia macrops* Erwin & Zamorano, **sp. n.**, Concordia, Río Uruguay, Argentina, *Asklepia marchantaria* Erwin & Zamorano, **sp. n.**, Ilha de Marchantaria, Lago Camaleão, Brazil, *Asklepia marituba* Zamorano & Erwin, **sp. n.**, Marituba, Ananindeua, Brazil, *Asklepia paraguayensis* Zamorano & Erwin, **sp. n.**, San Lorenzo, Río Paraguay, Paraguay, *Asklepia pakitza* Erwin & Zamorano, **sp. n.**, BIOLAT Biological Station, Pakitza, Perú, *Asklepia pulchripennis* (Bates, 1871), **comb. n**, Santarém, Rio Tapajós, Brazil, *Asklepia samiriaensis* Zamorano & Erwin, **sp. n.**, Boca del Río Samiria, Perú, *Asklepia stalametlitos* Zamorano & Erwin, **sp. n.**, Guayamer, Río Mamoré, Bolivia, *Asklepia strandi* Liebke, 1938, Guyana, *Asklepia surinamensis* **sp. n.**, l’Hermitage, Surinam River, Surinam, *Asklepia vigilante* Erwin & Zamorano, **sp. n.**, Boca del Río Samiria, Perú. Imágenes de los adultos de los 18 géneros son proporcionadas.

## Introduction

One of the major lacunae in our knowledge of tropical Carabidae is the Tribe Lachnophorini whose 15 Western Hemisphere genera (mostly Neotropical) and three Eastern Hemisphere genera (Paleotropical and Australian) have never been fully revised, nor have two of the Eastern Hemisphere genera been associated formally with the Tribe, until now. Lachnophorini is a tribe known mostly from 19th century isolated species descriptions and lists, or a general coverage in papers of broader scope (Ball and Bousquet 2000; Erwin et al. 2010; Reichardt 1977). The sole exception is Liebherr’s (1988) detailed treatment of the few Caribbean species arrayed in only four of the 18 genera recognized herein. None of the 18 genera have received a complete modern taxonomic revision. Jeannel (1948), while treating *Selina westermanni* Motschulsky from Madagascar, attempted a classification of groups he knew, or had access to, namely *Selina*, *Lachnophorus*, *Anchonoderus*, *Ega*, and *Calybe*. Typical of his sense of relationships, as with most French entomologists of his era, he regarded the group as the Lachnophoridae, with two Subfamilies, the Anchonoderitae and the Lachnophoritae. He also included a Péringuey species, *Amoebaea mashuna* Péringuey, as aligned with *Selina* in his Selinini. The name *Amoebaea* has since been recognized as a junior synonym of *Smeringocera* Chaudoir, now classified in the Odacanthini, Odacanthina (Lorenz 2005). We here reject provisionally Jeannel’s proposed arrangement on the grounds that he did not have enough lachnophorine taxa available (only 5 of 18) to make a sound classification. “Provisionally” is inserted on the basis that further investigation might reveal that some of Jeannel’s classification is actually sound, not likely, but possibly. Based on the full range of lachnophorine genera, we offer a different arrangement (see below).

The larva of only one species of the tribe has been described (Liebherr 1983). Many museums have numerous adult specimens of several of the genera and they remain in the unidentified section of those collections, for the most part. Several of the genera are inadequately defined and some of those infrequently collected. Because the Smithsonian’s National Museum of Natural History (NMNH) has an extensive collection of Lachnophorini, we decided to prepare this synopsis to lay the groundwork for future taxonomic work on these morphologically and strikingly varied beetles. Since 1971, the senior author (TLE) has been collecting Lachnophorini adults as part of his work in the rainforests of Middle and South America. The junior author (LSZ), as Erwin's Smithsonian Intern (2001–2012) at the NMNH from Colombia’s Universidad de los Andes in Bogotá, expressed an interest in learning carabid taxonomy; therefore, an opportunity presented itself for us to launch a long term taxonomic project on the genera and species of Lachnophorini (See Summary overview and future directions, below).

We were intrigued that Bates (1871) described four species in the genus *Eucaerus* (that here became reassigned to *Asklepia* Liebke) from the shores of the Amazon drainage system. Liebke (1938) without knowing of the Bates species erected a monobasic genus with his own species, *Asklepia strandi*, as the type species (Guyana), and that was based on a single specimen subsequently apparently lost during World War II (cf. Mroczkowski 1960). Reichardt (1974) then used his own misidentified specimens (from Santarém and vicinity) believing them to be *Asklepia strandi* to compare to his “new” species, *Asklepia ocellatus* Reichardt, which is actually the Bembidiini: Tachyina *Liotachys antennatus* Bates (Erwin, in Reichardt 1977). Still further, the specimens that Reichardt believed to be *Asklepia strandi* Liebke represent a new species that Bates did not collect at Santarém, even though he found a total of three species there during his years on the Amazon River system (Bates 1880). Given this historical comedy of errors, we provide a revision of the genus *Asklepia* Liebke and resolve several of those errors, in addition to describing many new species from various locations across South America from Argentina to Surinam and southeastern Colombia.

With the discovery of the minute species *Gehringia olympica* Darlington, 1933 and its later southern counterparts, the explosion of discovery of new species of anillines in the Western Hemisphere (Sokolov 2011; Sokolov et al. 2004), *Argentinatachoides* Sallenave et al. (2008), *Andinodontis* Erwin et al. (2010), and the present revision of *Asklepia* Liebke, we predict that many of the minute Carabidae remain undiscovered and we had better have a finer awareness of the next smaller fractal universe for this diverse family, if we are truly to understand it. Thus, we introduced this synopsis by way of poems, in part because of fractal universe sizes, as in set theory, applies to beetles, and in part as predators and parasites that attack and consume prey smaller than themselves. While the *Asklepia* species, covered herein, are very small as adults (less than 3.74 mm ABL), we pondered, what do they eat as adults? And, perhaps more interestingly, what do they eat as larvae. We provide size ranges in the Tables, and we noted their comparatively vast size ranges (SBL) within some well-collected species which led us to wonder if the larvae are ectoparasitic in their larval stages (e.g., see Erwin 1979, Frank et al. 2009).

**Figure 1.**
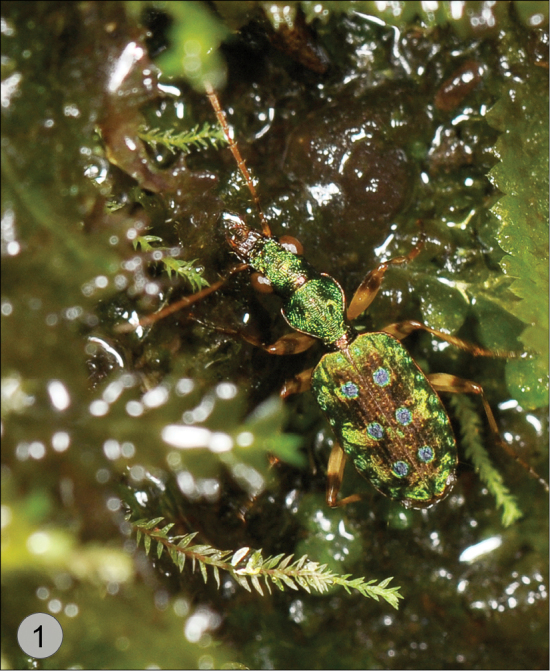
*Quammenis spectabilis* Erwin. Live individual in its natural hygropetric habitat in Costa Rica. Photo credit: K. Taro Eldredge, University of Kansas.

**Figure 2.**
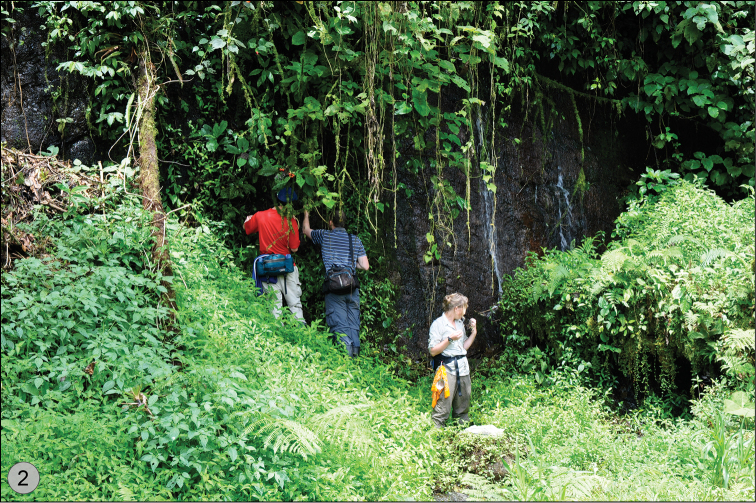
Natural hygropetric habitat in Costa Rica of *Quammenis spectabilis* Erwin with Andrew Short and his University of Kansas grad students Crystal Maier and Clay McIntosh. Photo credit: K. Taro Eldredge, University of Kansas.

## Materials and Methods

As noted in several past contributions, methods and species concepts follow those previously described (Erwin and Kavanaugh 1981; Kavanaugh and Erwin 1991). The species validation and diagnosis format follows as closely as possible that suggested in Erwin and Johnson (2000).

For measurements, an image of the specimens was obtained using a Leica M420 stereoscope coupled to an EntoVision^TM^ system. The resulting image was processed using the software Cartograph version 7.2.5 by Microvision Instruments. The magnification on the zoom was set to calibrate the system and it is embedded into the file of the image. The image was opened with the software program Archimed version 6.1.4 also by Microvision and the Measure tool, was then used to determine the lengths of the various parts. A total of 419 images were obtained.

Measurements of length (ABL, SBL) and width (TW) follow those of Ball (1972) and Kavanaugh (1979): ABL (apparent body length), measured from apex of labrum to apex of longer elytron (in adults of this genus, the abdomen often protrudes beyond the elytral apex), thus the ABL often is much larger that the SBL; SBL (standardized body length), equals the sum of the lengths of the head measured from apex of clypeus to a point on midline at level of the posterior edge of compound eyes; PL (pronotum length) is measured from apical to basal margin along midline; LE (elytron length) is measured from apex of scutellum to apex of the longer elytron; and TW (total width) measured across both elytra at their widest point with suture closed. Note that not all specimens available were measured due to either incomplete or broken specimens, or because more than 33 specimens were available we limited to that as a statistically valid sample. Sexes were measured separately; however, we found slight differences among the species, hence we report measurements for both sexes in our Tables (see Appendix 1). For the *Asklepia* treatment below, we provide relative size terms based on the SBL as follows: small-size < 2.5 mm, medium-size > 2.5 mm to 3.0 mm, and large-size > 3.0 mm. For an explanation of the measurements and their incorporation in Appendix 1, see Erwin (2011c) and Erwin and Ball (2011). For the present study, we report the harmonic mean, as we believe it better reflects the central tendency than the arithmetic mean.

Attributes of the abdominal ventral sterna are referred to using the numbering system generally accepted in carabid studies, i.e., the sternum divided medially by the hind coxae is sternum II (the first being hidden) and the last visible is sternum VII (Liu et al. 2011).

In a revision of the genus *Pericompsus* (Erwin 1974), a problem was encountered with the term “stria” for features of their punctate elytra (i.e., the so called striae were *not* striae, rather they were rows of punctures). The result was the use of the term “interneur” to apply to the attribute lying between intervals. Through use of this term, one could describe the feature as interneur striate, punctate, striatopunctate, etc.

The same problem exists for the proximal end of the median lobe of the male genitalia. In Snodgrass (1935), the term “phallobase” is used, and we have adopted it here (see Erwin 2011c). So, by extension, in Carabidae, we can say phallobase hooded (e.g., Lebiini, Pseudomorphini), phallobase of two parallel sclerotized struts (basal trechines and *Andinodontis*), phallobase of two uneven struts (*Bembidion*), etc. Kavanaugh (pers. comm.) points out that with struts there is still a connecting membrane surrounding the struts forming a “bulb.” We have chosen the aedeagal illustration of a male *Asklepia laetitia* sp. n. (Fig. 61) to have the identifying code letters and these apply to all illustrations of male genitalia of *Asklepia* and the male genitalia of *Peruphorticus gulliveri* sp. n. (Fig. 17), although there are some subtle differences in the endophallus from those of *Asklepia*.

This study includes a total of thousands of specimens of Lachnophorini, and 383 adult specimens of *Asklepia*, all currently at the National Museum of Natural History, Washington, DC (NMNH). Among the *Asklepia*, three specimens were received from the AMNH (Lee Herman, Curator); 31 specimens from the University of Alberta, Edmonton, Canada (UASM) collected in Brazil and sent to us by George E. Ball; 14 specimens from the Carnegie Museum of Natural History (CMNH) (Robert L. Davidson, Collection Manager); and ten specimens from the Museum of Zoology at the University of São Paulo, Brazil (MZUSP) (Dra. Sônia Casari, Curator). Also studied were the lectotype of *Asklepia lebioides* Bates and holotypes of *Asklepia pulchripennis*, *Asklepia geminata*, and *Asklepia hilaris* from the Muséum National d‘Histoire Naturelle, Paris (MNHP, Azadeh Taghavian, Collection Manager) and a paralectotype of *Asklepia lebioides* from the Natural History Museum in London (BMNH, Beulah Garner, Curator). Primary type specimens of new species will be deposited in their countries of origin if required by legal agreements, or museums of ownership at the conclusion of our studies on this tribe. The habitus images of the adult beetles portray most of the character states referred to in the keys provided. Illustrations of male genitalia are standard for descriptive taxonomy of carabid beetles in both preparation and aspects presented, as is the presentations of the female genitalia. The habitus images of the adults were made with a Visionary Digital^TM^ high resolution imaging system rendered using Photoshop to become “Digital Photo-illustrations.” Figure 56 demonstrates an elytron divided into six quadrants that we use to describe color patterns. Figure captions include an ADP number, which is a unique identification number for the specimen that was illustrated or imaged and links the specimen and associated illustrations and/or images to additional information, such as collecting notes, in electronic databases at the NMNH.

Geographical data are presented for species based on all known specimens available at the time of manuscript preparation, including those in the literature. Geo-referenced data have been determined from locality information provided on specimen labels; only those exact georeferences reported in decimal degrees that are provided on the label are placed in quotes. Otherwise, we have estimated others as closely as possible from places, mileage, or other locality data listed on the label and searched with Google Earth Pro. Latitude and longitude for those are reported in decimal degrees and have been corrected from those reported on the labels, if necessary; our bottom line is that georeferences locality data reported herein are far more accurate than those provided on specimens labels.

Distribution maps are provided for the species of *Asklepia* (Figs 76–78). Here, vernacular names in English are proposed, as common names are becoming increasingly needed in conservation reports and studies, and/or agricultural and forestry applications. These names are based on criteria set forth in Erwin (2011a) and applied in Erwin (2011b).

## Accounts of taxa

### Supertribe Odacanthitae
Key to the Tribes of Odacanthitae of the Western Hemisphere

**Table:** 

1	Antennal scape very long and apically swollen, at least 1 ½ times longer than antennomere 3	Calophaenini Jeannel, 1942
1	Antennal scape short and robust, shorter than or about coequal in length with antennomere 3	2
2 (1’)	Pronotum elongate and cylindrical, often bulbous at basal third	Odacanthini Laporte de Castelnau, 1834
2’	Pronotum not elongate and cylindrical, normally subquadrate or cordate with explanate, or beaded margins and usually well-defined hind angles	Lachnophorini LeConte, 1853

#### Lachnophorini

Taxon classificationAnimaliaColeopteraCarabidae

LeConte, 1853

Lachnophorini LeConte, 1853: 370
Anchonodérides Lacordaire, 1854: 373
Eucaeri LeConte, 1861: 22
Egini G. Horn, 1881: 152

##### Diagnosis.

Body form ranges from *Agonum*-like in *Anchonoderus* Reiche adults (Fig. 52) to ant-like in those of *Ega* Laporte de Castelnau (Fig. 56), *Selina* Motschulsky (Fig. 20), and *Stenocheila* Laporte de Castelnau (Fig. 21). Mandibles are markedly falciform (subfalciform in *Anchonoderus*). Subgena with patch of setae ventrad the eye, or entire venter of head with sparse short vestiture (except in *Amphithasus, Aporesthus*, *Diplacanthogaster*, *Guatemalteca*, *Homethes*, *Lachnaces*, and *Quammenis*); antennomeres 2 and 3 fully setose (except in *Aporesthus, Diplacanthogaster*, *Guatemalteca*, *Homethes*, and *Quammenis*); apical palpomeres inflated or fusiform; apical labial palpomere with short setae (except in *Anchonoderus*, *Aporesthus*, *Guatemalteca*, *Homethes*, *Peruphorticus*, and *Pseudophorticus*); elytra obliquely truncate (and deeply sinuate in *Aeolodermus*, *Quammenis*, and *Stenocheila*); abdominal sterna with scattered setae (except in *Aporesthus*, *Diplacanthogaster*, and *Quammenis*); spermatheca bipartite, or derivable from a bipartite ground plan (cf. Liebherr 1988), but not yet checked in several genera. We note that the mentum is toothed or not in adults of lachnophorine genera; we have not used that here, but LSZ will do so in an upcoming phylogenetic study of the tribe.

##### Notes.

We have arrayed the lachnophorine genera in two subtribes based on vestiture and body form: Eucaerina LeConte contains *Amphithasus*, *Aporesthus*, *Asklepia*, *Eucaerus*, *Guatemalteca*, and *Lachnaces*, all of which have adults with little, or no general setation and except for *Amphithasus* are of planate body form; and Lachnophorina LeConte contains *Anchonoderus*, *Calybe*, *Ega*, *Euphorticus*, *Lachnophorus*, *Pseudophorticus*, *Peruphorticus*, and *Selina*, adults of which are richly invested with setae and/or pubescence and of a medium to markedly convex body form. Given that *Selina* is the only Eastern Hemisphere taxon in this group, its adult similarity to *Ega* adults may be convergence.

We note that *Amphithasus* is somewhat “forced” into the Eucaerina herein provisionally until such time that a major phylogenetic analysis can be undertaken either by a detailed morphological analysis, a molecular analysis, or desirably both. Attributes of the rarely collected adults of this genus are sufficiently distinctive that they may deserve a subtribe of their own (and that subtribe may also include three undescribed genera of which we have only six specimens and are reluctant to describe at present – one of these, with three species, has evolved somewhat parallel to the members of *Rhadine* LeConte, 1848, a platynine genus). In regard to *Quammenis*, we believe it to be closely associated with *Diplacanthogaster* Liebke, 1932 and *Stenocheila* Laporte de Castelnau, 1832 of South America; and if so, then both *Homethes* Newman, 1842 and *Aeolodermus* Andrewes, 1929 of the Old World need to be reconsidered because adults of *Aeolodermus* have much in common with adults of *Quammenis*. For the present, we treat these five genera as *incertae sedis* within the Lachnophorini.

##### References.

Andrewes (1929), Ball and Hilchie (1983), Ball and Bousquet (2000), Bousquet (2012), Chaudoir (1872), Darlington (1956), Erwin (1991, 2000, 2004), Erwin et al. (2012), Liebke (1932, 1938), Jeannel (1948), Liebherr (1983, 1988, 1990), Louwerens (1952), Reichardt (1967, 1974).

##### Key to the genera of the Lachnophorini

**Table:** 

1	Specimens from Australia, the Malay Archipelago, and/or the Philippines	17
1’	Specimens from Africa, Vietnam, and the Indian subcontinent (Habitus, Fig. 20)	*Selina* Motschulsky, 1858
1’’	Specimens from the Western Hemisphere	2
2(1’’)	Antennomeres 3 and 4 markedly elongate, length more than combined length of scape and pedicel combined and testaceous, antennomeres 5-11 markedly broad, flattened, and black (Habitus, Fig. 21)	*Stenocheila* Laporte de Castelnau, 1832
2’	Antennomeres 3 and 4 moderately elongate or not, length coequal to, or less than combined length of scape and pedicel, antennomeres 5-11 not broadened, cylindrical and color various	3
3(2’)	Pronotum with transverse rugae on disc and laterally with a setiferous dentiform projection; elytron with sutural apex obliquely truncate (Habitus, Fig. 8)	*Diplacanthogaster* Liebke, 1932
3’	Pronotum without transverse rugae on disc, or if so then laterally without a setiferous tooth; elytron with sutural apex rounded or acute	4
4(3’)	Elytron with 3 large ocellate fossae; apex deeply sinuate. Dorsal surface matte metallic green, fovea purple. Head and pronotum with numerous micro-rugosities (Habitus, Fig. 22)	*Quammenis* Erwin, 2000
4’	Elytron without ocellate fossae; apex obliquely truncate. Dorsal surface not green. Head and pronotum not rugose, but may be densely punctate	5
5(4’)	Elytron glabrous, with few fixed setae	6
5’	Elytron pubescent or densely setigerous	9
6 (5)	Elytron with dorsal surface dull or shiny – no trace of iridescence	7
6’	Elytron with dorsal surface moderately to markedly iridescent	14
7(6)	Form robust and convex. Elytral interneurs striate, or striatopunctate. Ultimate labial palpomere glabrous, fusiform, pointed, not acuminate (Habitus, Fig. 4)	*Amphithasus* Bates, 1871
7’	Form planate. Elytral interneurs punctate, punctures without connecting striae	8
8(7’)	Elytron bicolored, Pattern with dark pattern. Interneurs of discontinuous punctures, shallowly impressed; punctures slightly more impressed in basal and apical third of elytron. Ultimate labial palpomere pubescent, globose, subulate. Abdomen finely setose (Habitus, Figs 29–56)	*Asklepia* Liebke, 1938
8’	Elytron concolorous, black or infuscated, or with paler sutural interval. Interneurs of continuous punctures to apex, markedly impressed, “appearing” striatopunctate but fine punctures not connected with striations. Palpomeres fusiform, glabrous. Abdomen with only fixed ambulatory setae (Habitus, Fig. 6)	*Aporesthus* Bates, 1871
9(5’)	Pronotal disc with scattered supplemental robust black setae	10
9’	Pronotal disc without supplemental robust setae (Habitus, Fig. 5)	*Anchonoderus* Reiche, 1843
10(9)	Elytron with deep transverse depression across basal third; pronotum cylindrical with barely developed lateral bead	11
10’	Elytron without transverse depression, but disc may be broadly fossate at basal third	12
11(10)	Neck markedly constricted, narrower than dorsal diameter of eye from a dorsal aspect. Pronotum and head smooth and glabrous with few fixed setae, surface shiny and smooth (Habitus, Fig. 9)	*Ega* Laporte de Castelnau, 1835
11’	Neck not much constricted, broader than dorsal diameter of eye from a dorsal aspect. Pronotum and head pubescent, surface dull (Habitus, Fig. 7)	*Calybe* Laporte de Castelnau, 1834
12(10’)	Head and pronotum multipunctate, these markedly impressed and dense. Elytron with interneurs striatopunctate, rows of punctures markedly impressed, and with three shallow fossae in third interval (Habitus, Fig. 16)	*Peruphorticus* Erwin & Zamorano, gen. n
12’	Head and pronotum smooth, with scattered punctures or densely pubescent; intervals with fossae or not	13
13(12’)	Ultimate palpomeres fusiform, pointed (Fig. 28), not acuminate or subulate. Pronotum surface densely pubescent, feebly rugose and punctate (Habitus, Fig. 19)	*Pseudophorticus* Erwin, 2004
13’	Ultimate labial palpomeres basally globose, apically acuminate or subulate. Pronotum smooth, surface densely and finely pubescent, or with scattered long setae	16
14(6’)	Pronotum broad, rectangulate, apical and basal margin as wide as base of elytron, lateral margins slightly rounded. Maxillary palpus elongate, basal palpomere slim, longer than scape (Fig. 26). Ultimate labial palpomere acuminate (Habitus, Fig. 14)	*Lachnaces* Bates, 1872
14’	Pronotum narrowed at the base, more or less trapezoidal. Maxillary palpus not elongate, basal palpomere robust, about coequal in length with scape. Ultimate labial palpomere fusiform or subulate	15
15(14’)	Pronotum surface iridescent and smooth. Ultimate labial palpomere fusiform (Fig. 25) (Habitus, Fig. 12)	*Guatemalteca* Erwin, 2004
15’	Pronotum surface with numerous micro-punctures densely distributed; dull. Ultimate labial palpomere globose, subulate (Fig. 23) (Habitus, Fig. 10)	*Eucaerus* LeConte, 1853
16(13’)	Elytron with three fossae, basal fossa larger and spread across interneurs 2 and 3, mid and apical fossae centered on interneur 2. Ultimate labial palpomere subulate (Fig. 27) (Habitus, Fig. 15)	*Lachnophorus* Dejean, 1831
16’	Elytron without fossae. Ultimate labial palpomere acuminate (Fig. 24) (Habitus, Fig. 11)	*Euphorticus* Horn, 1881
17(1)	Abdominal sterna and dorsal surface of tarsomeres glabrous (Habitus, Fig. 13)	*Homethes* Newman, 1842
17’	Abdominal sterna and dorsal surface of tarsomeres with fine vestiture (Habitus, Fig. 3)	*Aeolodermus* Andrewes, 1929

### Synopsis of genera herein assigned to the Tribe Lachnophorini

#### Aeolodermus

Taxon classificationAnimaliaColeopteraCarabidae

Andrewes, 1929

**Figure 3–6.**
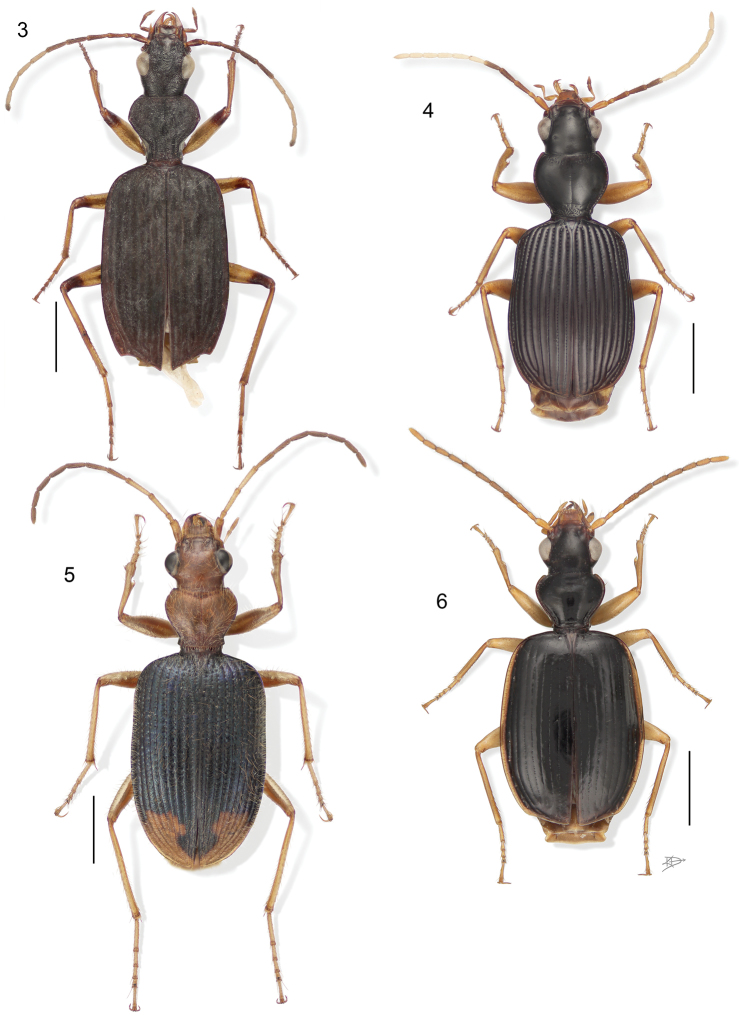
**3** *Aeolodermus emarginatus* Chaudoir. Digital Photo-illustration. Habitus, dorsal aspect, based on specimen ADP133799 from Mabatobato, Luzon, Philippines **4** *Amphithasus* sp. Digital Photo-illustration. Habitus, dorsal aspect, based on specimen ADP023719 from Rio Sucusari, Perú **5** *Anchonoderus apicalis* Reiche. Digital Photo-illustration. Habitus, dorsal aspect, based on specimen ADP132554 from nr. Atalaya, Perú **6** *Aporesthus* sp. Digital Photo-illustration. Habitus, dorsal aspect, based on specimen ADP132542 from Tena, Ecuador.

Velvet-topped Beetles Fig. 3

Aeolodermus Andrewes, 1929:368

##### Type species.

*Homethes emarginatus* Chaudoir, 1872:389

##### Way of life.

Size range – 7.5 mm to 8.0 mm; adults of this genus are active in May (1 ex. CAS) and have been found at lights (Andrewes 1929).

##### Distribution.

Indonesia, Malaysia, and the Philippines.

##### Notes.

Chaudoir (1872) believed his species was close to *Stenocheila* Laporte de Castelnau; however, Andrewes (1929) followed the advice of Sloane (in litt. or pers. comm.) and left it in the “Anchomenini.” Darlington (1956) followed Andrewes; however, we believe Chaudoir was clearly correct and we have classified it here within the Lachnophorini along with *Stenocheila*, *Diplacanthogaster*, and *Homethes*.

##### References.

Darlington (1956); Liebherr (1990); Moore et al. (1987).

#### Amphithasus

Taxon classificationAnimaliaColeopteraCarabidae

Bates, 1871

Double white-horned beetles Fig. 4

Amphithasus Bates, 1871:32
Amphitasus Bates: Erwin (1991), Lorenz (1998a), Ball and Bousquet (2000), Bousquet (2012).

##### Type species.

*Amphithasus truncatus* Bates, 1871:32

##### Way of life.

Size range – 3.5 mm to 6.0 mm; adults of this genus are found singly at night on wet leaf litter at the margins of swamps and medium-sized rivers, and on dry trails in primary rainforest.

##### Distribution.

Brazil, Colombia, Ecuador, Perú.

##### Notes.

Currently, two described species are assigned to this genus, however, there are five species represented in the NMNH collection. There is a need for a taxonomic revision of the group. *Anchomenus elegans* Dejean, 1831:725, was placed in “*Amphithasus*” by Lorenz (1998a), but we question his generic placement. Recourse to the type in Paris will solve the problem.

##### References.

Erwin (1991); Ball and Bousquet (2000); Bousquet (2012).

#### Anchonoderus

Taxon classificationAnimaliaColeopteraCarabidae

Reiche, 1843

Narrow thorax beetles Fig. 5

Anchonoderus Reiche, 1843:38
Axylosius Liebke, 1936:461 (Lorenz 1998a)

##### Type species.

*Platynus elegans* Brullé, 1838:25 [=*Anchonoderus eximius* (Audouin), 1836:34]

##### Way of life.

Size range – 4.5 mm to 8.5 mm; adults of this genus are found on the muddy banks of rivers and streams where they run in the sun from crack to crack in the baked mud. Other ubiquitous species are found throughout the forest where there is sandy soil and thin dry leaf litter, as well as on upper parts of river banks in thin layers of leaf litter. However, most species occur at stream margins among small stones and gravel and in flood debris along upper stream margins. The fact that only 27 species are described from the Neotropics and 11 occur at a single locality at Pakitza, Perú indicates the identification of species is impossible without recourse to types. A taxonomic revision of the group is need.

##### Distribution.

Arizona and Texas south to Argentina, including many of the Caribbean islands.

##### Notes.

Currently, 27 described species are assigned to this genus.

##### References.

Ball and Hilchie (1983); Liebherr (1988); Erwin (1991); Ball and Bousquet (2000); Bousquet (2012); Erwin et al. (2012).

#### Aporesthus

Taxon classificationAnimaliaColeopteraCarabidae

Bates, 1871

Perplexing beetles Fig. 6

Aporesthus Bates, 1871:103
Phaedrusium Liebke, 1941:249: Synonymy by Erwin, 1991:44.

##### Type species.

*Aporesthus anomalus* Bates, 1871:103

##### Way of life.

Size range – 4.0 mm to 5.5 mm; these very interesting small beetles occur on the underside of suspended logs and branches that straddle small to medium sized streams in the rainforest. They run to the top when the underside is splashed with water and when the streams rise due to heavy rainfall. Nothing more has been published about species of this genus.

##### Distribution.

Bolivia, Brazil, Guyana, Guyane, Ecuador, Paraguay, Perú, Surinam.

##### Notes.

Currently, three described species are assigned to this genus; however, ten species are represented in the NMNH collection. A taxonomic revision of the group is needed. Identification of species is impossible without recourse to types.

##### References.

Ball and Hilchie (1983) as *Phaedrusium*; Liebherr (1988) as *Phaedrusium*; Erwin (1991); Ball and Bousquet (2000); Bousquet (2012); Erwin et al. (2012).

### *Asklepia* Liebke, 1938 (see revision below)

#### Calybe

Taxon classificationAnimaliaColeopteraCarabidae

Laporte de Castelnau, 1834

**Figure 7–10.**
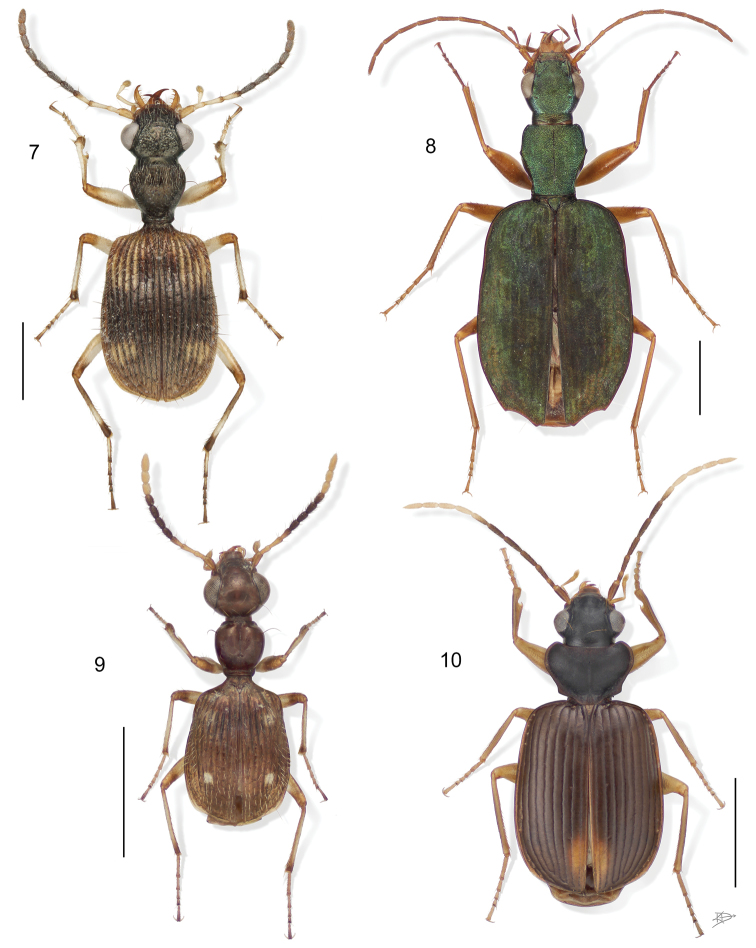
**7** *Calybe* sp. Digital Photo-illustration. Habitus, dorsal aspect, based on specimen ADP132561 from Pakitza, Perú **8** *Diplacanthogaster bicolor* Liebke. Digital Photo-illustration. Habitus, dorsal aspect, based on specimen ADP133817 from Ouro Preto, Brazil **9** *Ega* sp. (no described species of this genus is known from Perú). Digital Photo-illustration. Habitus, dorsal aspect, based on specimen ADP132560 from Pakitza, Perú **10** *Eucaerus* sp. Digital Photo-illustration. Habitus, dorsal aspect, based on specimen ADP132552 from Pakitza, Perú.

Ant-like hut beetles Fig. 7

Calybe Laporte de Castelnau, 1834:92
Chalybe Lacordaire, 1854:378

##### Type species.

*Calybe leprieuri* Laporte de Castelnau, 1834:92

##### Way of life.

Size range – 3.1 mm to 4.9 mm; species of this genus not only have color patterns and movements like ants, but also have body constrictions giving them near ant-like proportions. They live near water bodies usually on steep slopes just above the margin on damp clay soils.

##### Distribution.

Northwestern México south to Argentina.

##### Notes.

Currently, eight described species are assigned to this genus; however, many more species are represented in the NMNH collection. There is a need for a taxonomic revision of the group. Identification of species is impossible without recourse to types.

##### References.

Ball and Hilchie (1983); Liebherr (1983; 1988), Erwin (1991); Ball and Bousquet (2000), Bousquet (2012); Erwin et al. (2012).

#### Diplacanthogaster

Taxon classificationAnimaliaColeopteraCarabidae

Liebke, 1932

Two-cornered beetles Fig. 8

Diplacanthogaster Liebke, 1932:148

##### Type species.

*Diplacanthogaster bicolor* Liebke, 1932

##### Way of life.

Size range – 7.0 mm to 9.0 mm; the single species of this genus is active in April, July, and August, and probably in proximity of water.

##### Distribution.

Brazil.

##### Notes.

Reichardt (1967) regarded this genus as congeneric with *Stenocheila* Laporte de Castelnau. However, his study shows he had no male specimens of the latter for comparisons. We have presented in the key to genera above the attributes that justify Liebke’s separation of the two genera and not Reichardt’s synonymy.

##### References.

Liebke (1932), Reichardt (1967).

#### Ega

Taxon classificationAnimaliaColeopteraCarabidae

Laporte de Castelnau, 1835

Ant-like waterside beetles Fig. 9

Ega Laporte de Castelnau, 1835: 93

##### Type species.

*Ega formicaria* Laporte de Castelnau, 1835: 93

##### Way of life.

Size range – 2.8 mm to 4.7 mm; species of this genus not only have color patterns and movements like ants, they have body constrictions, giving them ant-like proportions, even more so than their probable adelphotaxon, *Calybe*. They live near water bodies usually on steep slopes above the water on damp clay soils, in the company of similar-sized ants. They catch small arthropod prey on the run much like tiger beetles. Identification is very difficult without recourse to types; the genus is in need of a taxonomic revision.

##### Distribution.

Southern USA (CA to GA, FL) south to Argentina. Not known from the Caribbean islands.

##### Notes.

Currently, 17 described species are assigned to this genus, some of which are likely *Calybe* species; however, many more true *Ega* species are represented in the NMNH collection. A taxonomic revision of the group is needed. Identification of species is impossible without recourse to types.

##### References.

Liebherr (1983); Erwin (1991); Ball and Bousquet (2000); Bousquet (2012); Erwin et al. (2012).

#### Eucaerus

Taxon classificationAnimaliaColeopteraCarabidae

LeConte, 1853

Easy fit beetles Figs 10 , 23

Eucaerus LeConte, 1853:386

##### Type species.

*Eucaerus varicornis* LeConte, 1853:387

##### Way of life.

Size range – 2.4 mm to 5.5 mm; these species are all hygrophilous, occurring in rotting leaf litter in densely shaded wet situations, stream sides, or swamps, and at margins of open marshes. They are common in some places; however, they run fast and it is difficult to obtain series without a lot of work. The opposite is true at other places where 100 or more individuals will flush together from a third of a square meter of wet leaf litter. Their way of life is unknown, but they likely are predatory on small arthropods.

##### Distribution.

Maryland, USA south through Texas and Florida to Brazil, including the larger Caribbean islands, México, and Central America.

##### Notes.

Currently, 10 described species are assigned to this genus; however, several more species are represented in the NMNH collection. A taxonomic revision of the group is needed. Identification of species is impossible without recourse to types.

##### References.

Ball and Hilchie (1983); Liebherr (1988); Erwin (1991); Ball and Bousquet (2000); Bousquet (2012); Erwin et al. (2012).

#### Euphorticus

Taxon classificationAnimaliaColeopteraCarabidae

Horn, 1881

**Figure 11–14.**
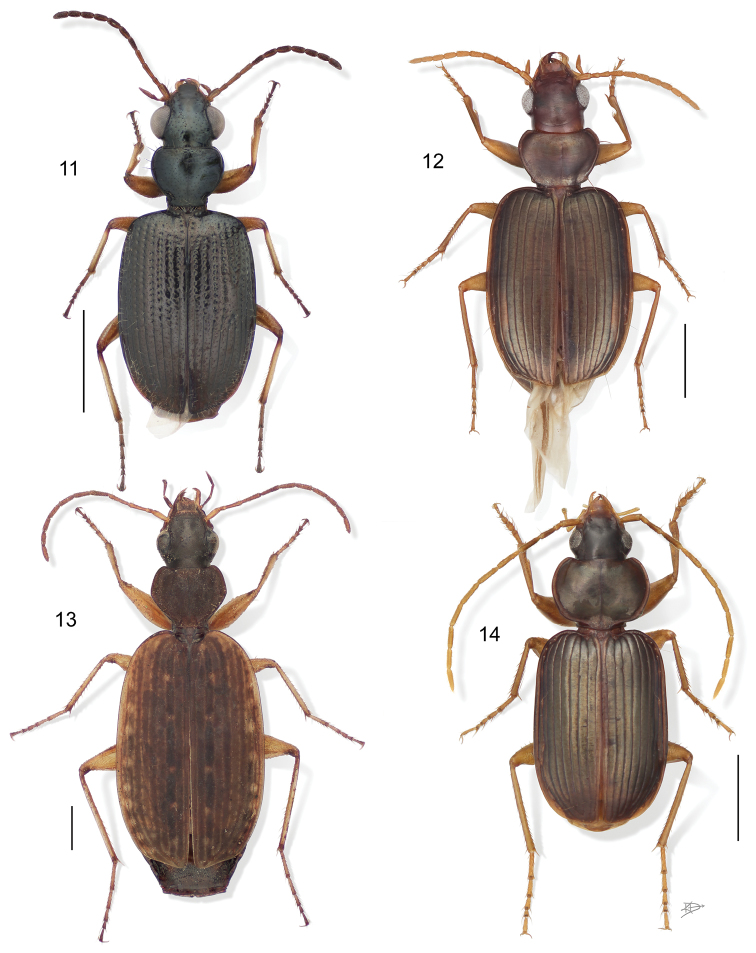
**11** *Euphorticus* sp. (only *Euphorticus pubescens* (Dejean) is known from México and this is not that species). Digital Photo-illustration. Habitus, dorsal aspect, based on specimen ADP132558 from Tapilulu, México **12** *Guatemalteca virgen* Erwin. Digital Photo-illustration. Habitus, dorsal aspect, based on specimen ADP132546 from nr. La Virgen, Costa Rica **13** *Homethes* sp. Digital Photo-illustration. Habitus, dorsal aspect, based on specimen ADP133797 from Australia **14** *Lachnaces* sp. (at present this genus has three described species, all from the upper Amazon Basin). Digital Photo-illustration. Habitus, dorsal aspect, based on specimen ADP132578 from Tambopata Reserved Zone, Explorer’s Inn, Perú.

Beauty-bearing beetles Figs 11 , 24

Euphorticus G. Horn, 1881:144

##### Type species.

*Lachnophorus pubescens* Dejean, 1831:30

##### Way of life.

Size range – 4.0 mm to 4.9 mm; these beetles are somewhat ubiquitous. They occur near water or at damp places, on a variety of soil types. Adults of all species are dark, often black, and one new species from Paraguay is vividly metallic. Adults of another new species from Perú was found readily under clumps of cut grass in an open field, as well as on algae covered sandy clay running in the bright sunshine. Adults are attracted to lights.

##### Distribution.

Southern United States south to southern Brazil, including some Caribbean islands.

##### Notes.

Currently, four described species are assigned to this genus; however, two additional new species are represented in the NMNH collection. A taxonomic revision of the group is needed. Identification of species is impossible without recourse to types.

##### References.

Liebherr (1988); Erwin (1991); Ball and Bousquet (2000); Bousquet (2012); Erwin et al. (2012).

#### Guatemalteca

Taxon classificationAnimaliaColeopteraCarabidae

Erwin, 2004

Shiny downy-bellied beetles Figs 12 , 25

Guatemalteca Erwin, 2004:12

##### Type species.

*Guatemalteca virgen* Erwin, 2004:12

##### Way of life.

Size range – 4.3 mm to 5.2 mm; these small beetles occur along small rocky streams in the highlands and in wet leaf litter in the lowlands; they take cover in the day time under stones or stream side debris. Many adults have been collected in Guyane with flight intercept traps (FITs).

##### Distribution.

Costa Rica; Guatemala; Guyane; México; Perú.

##### Notes.

Currently, one described species is assigned to this genus; however, two additional new species are represented in the NMNH collection, one from Guyane and another from Perú. A taxonomic revision of the group is needed.

##### References.

Erwin (2004); Bousquet (2012); Erwin et al. (2012).

#### Homethes

Taxon classificationAnimaliaColeopteraCarabidae

Newman, 1842

Velvet-backed beetles Fig. 13

Homethes Newman, 1842:402

##### Type species.

*Homethes elegans* Newman, 1842:402

##### Way of life.

Size range – 8.5 mm to 9.0 mm; these small beetles, at least in some species, are known to fly. According to Moore et al. (1987) they are predaceous.

##### Distribution.

Australia, Indonesia, Malaysia, and the Philippines.

##### Notes.

Nine species are known to occur in Australia (Moore et al. 1987).

##### References.

Darlington (1956); Moore et al. (1987); Liebherr (1990).

#### Lachnaces

Taxon classificationAnimaliaColeopteraCarabidae

Bates, 1872

Dull downy-bellied beetles Figs 14 , 26

Lachnaces Bates, 1872:201

##### Type species.

*Lachnaces sericeus* Bates, 1872:202

##### Way of life.

Size range – 3.2 mm to 5.4 mm; these small beetles occur in Amazonian inundation forests of the Varzea and Igapó systems. Adults occur in very wet leaf litter and in rotten wood in swampy areas. One adult was found by insecticidal fogging of the suspended fronds of an *Astrocaryum* palm; no doubt it was seeking refuge from inundation.

##### Distribution.

Brazil, Perú.

##### Notes.

Currently, three described species are assigned to this genus: however, eight species are represented in the NMNH collection. A taxonomic revision of the group is needed.

##### References.

Ball and Hilchie (1983); Erwin (1991); Ball and Bousquet (2000).

#### Lachnophorus

Taxon classificationAnimaliaColeopteraCarabidae

Dejean, 1831

**Figure 15–18.**
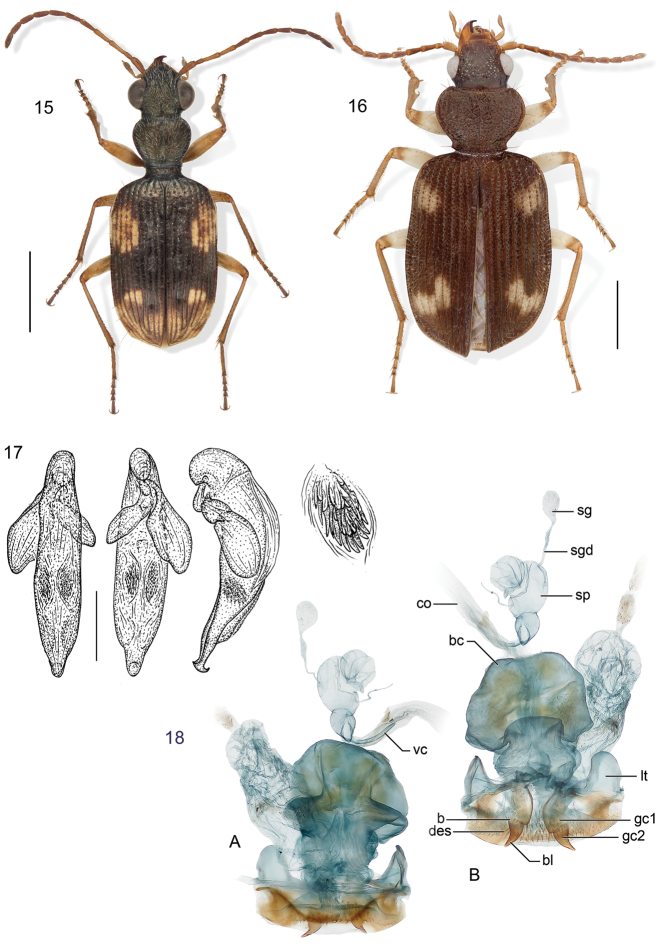
**15** *Lachnophorus* sp. Digital Photo-illustration. Habitus, dorsal aspect, based on specimen ADP132570 from Pakitza, Perú **16** *Peruphorticus gulliveri* sp. n. Digital Photo-illustration. Habitus, dorsal aspect (specimen slightly teneral) based on specimen BIOLAT #10190 from Pakitza, Perú **17** *Peruphorticus gulliveri* sp. n. Illustrations, male aedeagus, dorsal, ventral, left lateral aspects, and details of armature of endophallus. BIOLAT/COLE13107, Pakitza, Perú. See Fig. **61** for labeled attributes **18** *Peruphorticus gulliveri* sp. n. Digital Photo-illustration, female genitalia, based on specimen BIOLAT/COLE16860 from Pakitza, Perú. **A** Ventral aspect. Legend, **bc** bursa copulatrix; **co** common oviduct; **sg** spermathecal gland; **sgd** spermathecal gland duct; **sp** spermatheca. dorsal aspect; **vc** villous canal; **lt** laterotergite; **gc1** gonocoxite 1; **gc2** gonocoxite 2. **B** Dorsal aspect. Legend, **b** base of gonocoxite 2; **bl** blade of gonocoxite 2; **des** dorsal ensiform seta.

Down-bearing beetles Figs 15 , 27

Lachnophorus Dejean, 1831:28
Stigmaphorus Motschulsky, 1862:48
Aretaonus Liebke, 1936:461

##### Type species.

*Lachnophorus pilosus* Dejean, 1831:29

##### Way of life.

Size range – 4.0 mm to 6.3 mm; these small beetles are found on bare sandy stream banks in shady forests, or on sand in sunny areas along rivers. Their coloration pattern makes them appear like ants, and movements are ant-like, but they do not necessarily occur with ants. They catch small arthropod prey on the run much like tiger beetles.

##### Distribution.

Southern United States south to Uruguay, including the larger Caribbean islands.

##### Notes.

Currently, 42 described species are assigned to this genus. A taxonomic revision of the genus is needed. Identification is very difficult without recourse to types.

##### References.

Liebherr (1988); Erwin (1991); Ball and Bousquet (2000); Bousquet (2012); Erwin et al. (2012).

#### Peruphorticus

Taxon classificationAnimaliaColeopteraCarabidae

Erwin & Zamorano gen. n.

http://zoobank.org/D1D6C243-89D4-433C-8EEE-F75CD80EAAA6

Peruvian beauty-bearing beetles Figs 16 , 74 , 75 , 79

##### Type species here designated.

*Peruphorticus gulliveri* Erwin & Zamorano, sp. n.

##### Proposed english vernacular name.

Peruvian beauty-bearing beetles.

##### Diagnosis

(Figs 63, 74, 75). With the attributes of Lachnophorini (see above), body form robust, and occiput and pronotum deeply and coarsely punctate, the latter also partly rugose. Ultimate palpomeres elongate and slightly acuminate. Elytral interneurs striatopunctate, deeply engraved; intervals convex and multipunctate and with three shallow fossae in third interval; apex subtruncate, outer angle rounded.

##### Dispersal potential.

The wings are fully developed, thus it is likely that these beetles are moderate to strong flyers.

##### Distribution.

As currently recorded, member species are known from Costa Rica, Ecuador, and Perú.

##### Way of life.

These small beetles are found in dry or wet leaf litter in Amazonian rainforests independent of water bodies, and particularly along dusty trails. In Costa Rica, they occur at higher elevations in cloud forest. At least two species with adults somewhat reddish in color are found only on the lateritic soils pushed up by members of the ant genus *Atta* during nest building and nest maintenance activities.

##### Notes.

Nine species are represented in the NMNH, in addition to the one described herein. It is probable that the nine additional species are new to science; however, types of the genera *Lachnophorus* and *Euphorticus* need to be examined to make sure they are rightfully assigned to those genera and not *Peruphorticus*.

#### Peruphorticus gulliveri

Taxon classificationAnimaliaColeopteraCarabidae

Erwin & Zamorano sp. n.

http://zoobank.org/1A142F39-A267-4708-8484-E25CC095AE84

Gulliver’s beauty-bearing beetle Figs 16 , 17 , 18 , 79

##### Holotype.

**Perú**, Madre de Dios, BIOLAT Biological Station, Pakitza, Zone 5, Trocha Aguajal 92, 11.9427°S, 71.2926°W, 324m, 28 October 1990 (T.L. Erwin)(NMNH: BIOLAT10190, male).

##### Derivation of specific epithet.

The epithet “*gulliveri*” is a Latinized eponym, genitive case, based on the name made famous by the Irish writer Jonathan Swift in 1726, namely Gulliver’s Travels. We so name this species because of its very large size in comparison to its congeners, reminding us of Gulliver’s travels on the island of Lilliput.

##### Proposed english vernacular name.

Gulliver’s beauty-bearing beetle.

##### Diagnosis.

With the attributes of the genus *Pseudophorticus*, as described by Erwin (2004) and as noted above, and large-sized for the genus. Adults with fuscous dorsum and venter and shiny throughout, the elytron with fulvous subhumeral and subapical spots, legs and mouthparts testaceous, antennomeres pale to slightly infuscated. Microsculpture absent from head and pronotum, of fine isodiametric sculpticells on most of elytron, surface luster somewhat shiny and slightly aeneous in some specimens. Head multipunctate, setiferous, punctures of occiput large and deep; pronotum multipunctate and rugose, setiferous, punctures large and deep; elytron striatopunctate, striae well-impressed, intervals moderately convex and multipunctate, setiferous. Labial palpomere 3 (Fig. 16) elongate and slightly acuminate (apex narrowly rounded). Pronotum lateral margin narrowly explanate throughout. Males with tarsomeres 1–3 slightly widened each with biserial rows of adhesive vestiture on ventral surface.

##### Description.

(Figs 16, 17, 18). ***Habitus*:** (Fig. 16). ***Size*:** [See also Appendix 2] Large for the genus; ABL = 5.52–6.86 mm, SBL = 5.21–6.05 mm, TW (total width) 2.30–3.6 mm, LP = 1.10–1.29 mm, WP = 1.52–1.81 mm, LE = 3.37–4.05 mm. ***Color*:** See diagnosis above. ***Luster*:** See diagnosis above. ***Head*** (Fig. 16): labrum rectangulate, sexsetose, clypeus convex with small tuberculate at middle and clearly demarcation from frons. Frons shallowly convex with moderately depressed lateral sulci, occiput shallowly domed; neck broad. Eyes large, moderately convex, longitudinal diameter coequal with length of antennomere 2 + 3; gena very short and flat. ***Prothorax*.** Pronotum (Fig. 16) markedly broad and cordiform, about half as long as head (LP/LH: mean, males 1.51, females 1.49), moderately broader than long (W/L, mean: males, 1.39, females, 1.41); margin narrowly explanate with seta at anterior third and at hind angle; base convex and laterally depressed; hind angle flared laterally, slightly obtusely produced; multipunctate and rugose, setiferous, punctures large and deep. ***Pterothorax*.** Normal for tribe. Elytron about same width as pronotum (WP/TW: mean, both sexes, 0.628), moderately convex, intervals moderately convex and slightly more so laterally, interval 3 with very shallow fossae. Hind wings fully developed. ***Legs*** (Fig. 16). Overall, normal for subgenus. Male front tarsus with tarsomeres 1–3 slightly dilated and each ventrally with two biserial rows of white articulo-setae. ***Abdominal sterna*.** Setation as for genus. ***Male genitalia*** (Fig. 17, see Fig. 61 for attribute labels). Median lobe with basal lobe about one-fifth length of shaft, basal opening small. Shaft moderately robust, sinuate ventrally, dorsally membranous except for two short sclerotized strips flanking distal part of long ostial opening; in ventral aspect constricted toward rather small hooked apex, preapically with prominent ridges lateral to a central trough, in lateral aspect, ridges converge in a small but prominently projected point. Parameres broad, apices rounded; left paramere longer than right paramere, about three quarters length of shaft (measured in left lateral aspect). Endophallus with two fields of densely packed spines. ***Female genitalia*** (Fig. 18). Ovipositor with broad laterotergite (**lt**) and two gonocoxites (**gc 1, gc 2**); gonocoxite 1 spinose (short thick setae); gonocoxite 2 moderately falcate, base (**b**) large, broad, blade (**bl**) rather short, with two dorsal ensiform setae (**des**), ensiform setae short; without ventral preapical nematiform setae. Reproductive tract proximally with short, broad bursa copulatrix (**bc**), continuous at its distal end with common oviduct (**co**) and short bulbous bifid spermatheca (**sp**), latter of three bulb sections; villous canal (**vc**) extended from near base of spermatheca well up in common oviduct; spermathecal gland (**sg**) bulbous; spermathecal gland duct (**sgd**) long, slender, attached to spermatheca at middle bulb.

##### Dispersal potential.

These beetles are macropterous and probably capable of flight. They are moderately swift and agile runners.

##### Distribution.

(Fig. 79) Currently known from Ecuador and Perú, but likely more widespread.

##### Way of life.

See Erwin (2004) for a general description under *Pseudophorticus*; some undescribed species mentioned there belong to the present new genus, not *Pseudophorticus*, for example, the reddish species present on leaf-cutter ant nests. This present species, *Peruphorticus gulliveri*, is very common running at night on bare spots on trails and in thin layers of leaf litter.

##### Other specimens examined.

**Ecuador**, Napo, 75km E Coca, 16 September 1990 (D.L. Pearson)(NMNH: ADP133803, female paratype). **Perú**, Loreto, circa Explornapo Camp, Rio Napo, Cocha Shimagai, 3.3563°S, 73.0467°W, 88m, 5 June 1992 (T.L. Erwin, E. Pfuno S., F. Pfuno S.)(NMNH: ADP023768, ADP023788, male paratypes); Explorama Lodge, Rio Sucusari, 3.257°S, 72.916°W, 101m, 6 June 1992 (T.L. Erwin, E. Pfuno, F. Pfuno)(NMNH: ADP053355, ADP053596, female paratypes), 7 June 1992 (T.L. Erwin, E. Pfuno, F. Pfuno)(NMNH: ADP053660, ADP053724, female paratypes); Explornapo Camp, Rio Sucusari, 3.225°S, 72.920°W, 95m, 7 June 1992 (T.L. Erwin)(NMNH: ADP053719, ADP053739, ADP008283, male paratypes, ADP008277, ADP008281, ADP008284, ADP008266, ADP008263, female paratypes), 18 June 1992 (T.L. Erwin)(NMNH: ADP009149, ADP009150, male paratypes, ADP009051, female paratype), 19 June 1992 (T.L. Erwin)(NMNH: ADP009261, ADP009262, ADP009263, ADP009264, ADP009265, ADP009266, ADP009267, male paratypes, ADP009060, female paratype), Rio Sucusari, Caño Yanamono, 3.257°S, 72.916°W, 101m, 30 May 1992 (T.L. Erwin, E. Pfuno, F. Pfuno)(NMNH: ADP051053, ADP051053 male paratypes, ADP051011, female paratype), 31 May 1992 (T.L. Erwin, E. Pfuno, F. Pfuno)(NMNH: ADP050771, female paratype), 22 June 1992 (T.L. Erwin, E. Pfuno, F. Pfuno)(NMNH: ADP010281, ADP010285, ADP010286, ADP010293, ADP010294, ADP010295, ADP010301, ADP010303, ADP010304, ADP010408, male paratypes, ADP010296, ADP010287, female paratypes), 24 June 1992 (T.L. Erwin, E. Pfuno, F. Pfuno)(NMNH: ADP010363, ADP010364, ADP010365, ADP010398, ADP010407, ADP010475, ADP010559, ADP010562, ADP010563, ADP010564, ADP010565, ADP010566, ADP010602, ADP010604, ADP010605, ADP010608, ADP010612, ADP010613, ADP010614, ADP010652, ADP010732, ADP010739, ADP010740, ADP010741, ADP010744, ADP010745, ADP010746, ADP010750, male paratypes, ADP010731, ADP010748, ADP010729, ADP010730, ADP010742, ADP010743, ADP010404, ADP010560, ADP010561, ADP010474, ADP010610, ADP010362, female paratypes); Pithecia, 5.1757°S, 74.655°W, 111m, 14 August 1989 (T.L. Erwin, G. Servat)(NMNH: ADP132743, male paratype, ADP132595, female paratype); Río Samiria, Boca Caño Inglés Camp, 5.1317°S, 75.0617°W, 117m, 20 August 1991 (G.E. Ball, D. Shpeley)(NMNH: ADP133807, male paratype); circa Pithecia, Cocha Shinguito, 5.1775°S, 74.6556°W, 111m, 26–29 August 1991 (G.E. Ball, D. Shpeley)(NMNH: ADP133809, male paratype); Río Samiria, Boca Caño Inglés Camp, 5.2265°S, 75.1058°W, 117m, 20 August 1991 (T.L. Erwin, M. Pogue, C. Reyes)(NMNH: ADP071551, female paratype); circa Pithecia, Cocha Shinguito, 5.1775°S, 76.6556°W, 112m, 25 August 1991 (T.L. Erwin, M. Pogue)(NMNH: ADP071181, female paratype), 26 August 1991 (T.L. Erwin, M. Pogue, C. Reyes)(NMNH: ADP071301, ADP071256, male paratypes); Madre de Dios, BIOLAT Biological Station, Pakitza, Zone 2, 11.9427°S, 71.2926°W, 324m, 5 September 1989 (T.L. Erwin)(NMNH: BIOLAT001189, BIOLAT008085, BIOLAT008086, BIOLAT008084, BIOLAT001191, BIOLAT007196, BIOLAT- 009090, BIOLAT002651, male paratypes), 5 February 1990 (T.L. Erwin)(NMNH: BIOLAT006698, BIOLAT006698, BIOLAT006693, BIOLAT007042, male paratypes), 10 February 1990 (NMNH: BIOLAT002650), 11 February 1990)(NMNH: BIOLAT007041), 13 February 1990 (NMNH: BIOLAT007176, male paratype), 18 October 1990 (E. Vega)(NMNH: BIOLAT008767, BIOLAT008769, BIOLAT008771, BIOLAT008788, male paratypes), 29 September 1991 (E. Vega)(NMNH: BIOLAT12607, female paratype), 29 September 1991 (T.L. Erwin, M.G. Pogue)(NMNH: BIOLAT12597, male paratype), 5 October 1991 (NMNH: BIOLAT13107, male paratype, BIOLAT13140, BIOLAT13199, female paratypes), 9 October 1991 (T.L. Erwin)(NMNH: BIOLAT13435, BIOLAT13436, BIOLAT13443, BIOLAT13445, BIOLAT13447, BIOLAT13446, BIOLAT13428, BIOLAT13471, BIOLAT13440, BIOLAT13444, BIOLAT13442, BIOLAT13449, BIOLAT13448, BIOLAT13473, BIOLAT13474, BIOLAT13441, male paratypes, 8 July 1992 (NMNH: BIOLAT16858, BIOLAT16860, female paratypes), 10 July 1992 (T.L. Erwin, E. Pfuno, F. Pfuno)(NMNH: BIOLAT17000, BIOLAT17001, BIOLAT17002, BIOLAT17004, BIOLAT17007, BIOLAT17008, BIOLAT17009, BIOLAT17005, BIOLAT17010, BIOLAT17011, BIOLAT17015, BIOLAT17018, BIOLAT17020, BIOLAT17021, BIOLAT17022, BIOLAT17023, BIOLAT17024, BIOLAT17028, BIOLAT17030, BIOLAT17019, BIOLAT17031, BIOLAT17033, BIOLAT17036, BIOLAT17037, BIOLAT17038, BIOLAT17043, BIOLAT17044, BIOLAT17045, BIOLAT17046, BIOLAT17049, BIOLAT17050, BIOLAT17051, BIOLAT17052, male paratypes, BIOLAT17003, BIOLAT17006, BIOLAT17012, BIOLAT17014, BIOLAT17016, BIOLAT17017, BIOLAT17026, BIOLAT17029, BIOLAT17032, BIOLAT17034, BIOLAT17035, BIOLAT17039, BIOLAT17041, BIOLAT17042, BIOLAT17053, BIOLAT16979, female paratypes), 12 July 1992 (NMNH: BIOLAT17107, BIOLAT17154, BIOLAT17142, BIOLAT17156, BIOLAT17155, male paratypes, BIOLAT17127, BIOLAT17151, female paratypes), 20 March 1992 (B. Brown, D. Feener)(NMNH: BIOLAT17440, male paratype), 28 October 1990 (T.L. Erwin)(NMNH: BIOLAT10190, male paratype), 20–30 September 1991 (T.L. Erwin)(NMNH: BIOLAT12606, male paratype), 7–13 October 1991 (T.L. Erwin)(NMNH: BIOLAT13238, BIOLAT13248, male paratypes), 24 June 1993 (T.L. Erwin, F. Pfuno)(NMNH: BIOLAT19108, male paratype); Puerto Maldonado, Explorers Inn, 12.819°S, 69.260°W, 207, 23 October 1982 (T.L. Erwin)(NMNH: ADP133815, male paratype), 31 August 1983 (T.L. Erwin)(NMNH: ADP133811, female paratype), 3 October - 15 November 1983 (N.E. Stork)(NMNH: ADP134017, ADP134021, ADP134023, ADP134025, ADP134027, male paratypes, ADP133815, female paratype), Rio Tambopata, Culpa de Guacamayos, 300m, October 1995 (A. Forsyth)(NMNH: ADP132491, female paratype).

#### Pseudophorticus

Taxon classificationAnimaliaColeopteraCarabidae

Erwin, 2004

**Figure 19–22.**
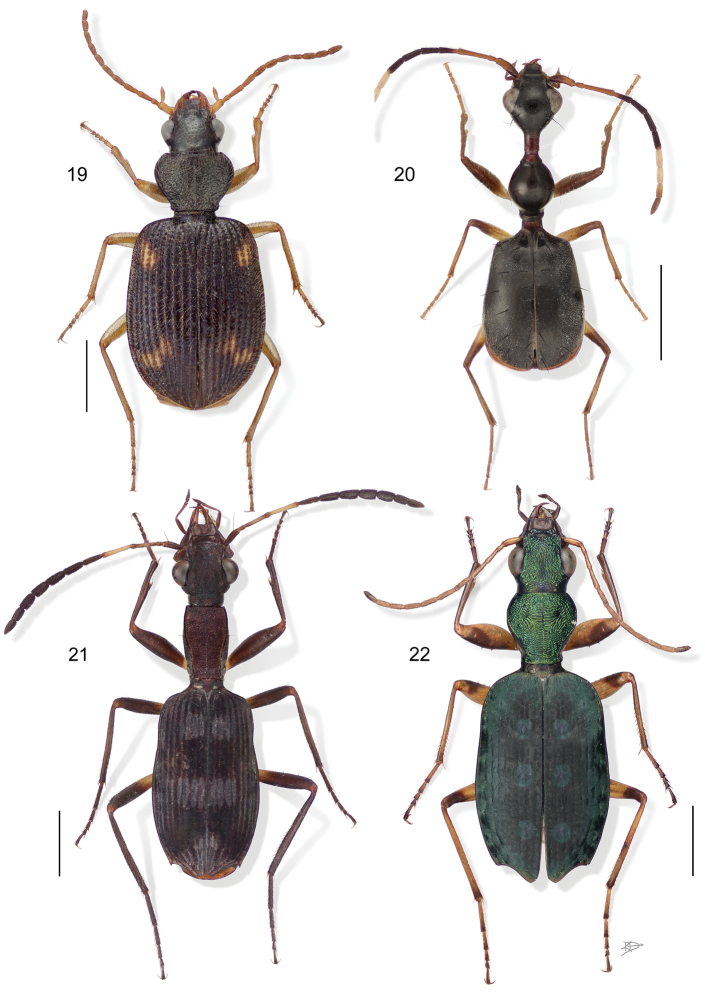
**19** *Pseudophorticus* sp. (all species in South America are either undescribed or placed in *Euphorticus* G. Horn). Digital Photo-illustration. Habitus, dorsal aspect, based on specimen ADP132538 from Pakitza, Perú **20** *Selina westermanni* Motschulsky. Digital Photo-illustration. Habitus, dorsal aspect, based on specimen ADP132536 from China Bay, Sri Lanka **21** *Stenocheila lacordairei* Laporte de Castelnau. Digital Photo-illustration. Habitus, dorsal aspect, based on specimen ADP133801 from Chapada dos Guimãres, Brazil **22** *Quammenis spectabilis* Erwin. Digital Photo-illustration. Habitus, dorsal aspect, based on specimen ADP100513 from Estacíon Zurqui, Costa Rica.

False beauty-bearing beetles Figs 19 , 28

Pseudophorticus Erwin, 2004:7

##### Type species.

*Pseudophorticus puncticollis* Erwin, 2004:8

##### Way of life.

Size range – 4.7 mm to 6.2 mm; these small beetles occur on the ground in rainforests; they are diurnal and run in clearings and on trails in open spots. Nothing is known about their way of life.

##### Distribution.

Costa Rica south to Perú and southeastern Brazil.

##### Notes.

Currently, one described species is assigned to this genus. Many undescribed species are represented in collections, misidentified as either *Euphorticus* or *Lachnophorus*; the genus is in need of a taxonomic revision. In order to do such a revision, one would need to study all the primary types of both *Euphorticus* and *Lachnophorus* to discover their correct generic assignments.

##### References.

Erwin (1991) as *incertae sedis*; Erwin (2004); Bousquet (2012); Erwin et al. (2012).

**Figures 23–28.**
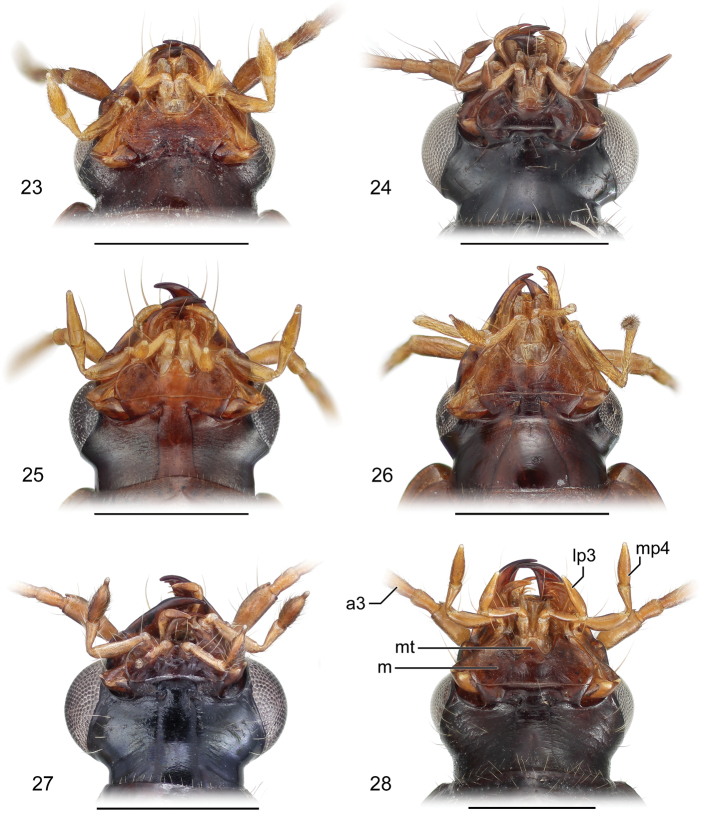
Mouthparts of adults. **23** *Eucaerus* sp. Mouthparts, ventral aspect, based on specimen ADP132552 from Pakitza, Perú **24** *Euphorticus* sp. Mouthparts, ventral aspect, based on specimen ADP132558 from Tapilula, México **25** *Guatemalteca virgen* Erwin. Mouthparts, ventral aspect, based on specimen ADP132546 from nr. La Virgen, Costa Rica **26** *Lachnaces* sp. Mouthparts, ventral aspect, based on specimen ADP132578 from Tambopata Reserved Zone, Explorer’s Inn, Perú **27** *Lachnophorus* sp. Mouthparts, ventral aspect, based on specimen ADP132570 from Pakitza, Perú **28** *Pseudophorticus* sp. Mouthparts, ventral aspect, based on specimen ADP132538 from Pakitza, Perú. Legend: **a3** Antennomere 3; **mt** Mentum tooth; **m** Mentum; **lp3** Labial palpomere 3; **mp4** Maxillary palpomere 4.

#### Quammenis

Taxon classificationAnimaliaColeopteraCarabidae

Erwin, 2000

Hygropetric green beetles Figs 1 , 2 , 22

Quammenis Erwin, 2000:279

##### Type species.

*Quammenis spectabilis* Erwin, 2000:280

##### Way of life.

Size range – 6.5 mm to 7.0 mm; these beetles occur on steep or vertical wet rocky surfaces with mosses and ferns and other hygrophilous plants in cloud forests (e.g., hygropetric habitats such as vertical waterfalls, seeps, wet rocks, etc. (see Fig. 2)).

##### Distribution.

Costa Rica.

##### Notes.

Currently, one described species is assigned to this genus.

##### References.

Erwin (2000); Bousquet (2012).

#### Selina

Taxon classificationAnimaliaColeopteraCarabidae

Motschulsky, 1858

Moon-necked beetles Fig. 20

Selina Motschulsky, 1858:110
Pselaphanax Walker, 1859:52
Steleodera Schaum, 1863:74
Ega Peringuey, 1896:146 [non Laporte de Castelnau, 1834]
Sphinctodera Fairmaire, 1901:128

##### Type species.

*Selina westermanni* Motschulsky, 1858:110

##### Way of life.

Size range – 3.9 mm to 5.0 mm; these small beetles occur on sandy banks of rivers (Lewis, 1882:480).

##### Distribution.

Africa, India, Madagascar, Sri Lanka, and Vietnam.

##### Notes.

Currently, six described species are assigned to this genus; names of five of them are listed by Lorenz (2005) as synonyms of *Selina westermanni* Mots. We believe that a revision is necessary. *Selina*, *Homethes*, and *Aeolodermus* are the only known members of Lachnophorini found in the Eastern Hemisphere.

##### References.

Erwin (1984); Bousquet (2012); Jeannel (1948).

#### Stenocheila

Taxon classificationAnimaliaColeopteraCarabidae

Laporte de Castelnau, 1832

Narrow-mouthed beetles Fig. 21

Stenocheila Laporte de Castelnau, 1832:12

##### Type species.

*Stenocheila lacordairei* Laporte de Castelnau, 1832:12

##### Way of life.

Size range – 7.8 mm to 9.3 mm; these beetles have been found on the campus of INPA at Manaus, Brazil. The area was an open managed unnatural habitat among buildings.

##### Distribution.

Bolivia, Brazil, Colombia, Guyane, Perú.

##### Notes.

This is a monobasic genus with a widespread species. See our notes under *Diplacanthogaster* Liebke, above.

##### References.

Reichardt (1967).

### Revision of the genus *Asklepia* Liebke, 1938

#### Asklepia

Taxon classificationAnimaliaColeopteraCarabidae

Liebke, 1938

Neat pattern-wing beetles

Asklepia Liebke, 1938:113

##### Type species.

*Asklepia strandi* Liebke, 1938:113.

##### Derivation of genus name.

Asklepius, a Greek God of healing. Why Liebke used this name is unknown. See: http://www.mythologydictionary.com/asclepius-mythology.html

##### Proposed english vernacular name.

Neat pattern-wing beetles.

**Diagnosis** (Figs 29–55, 57–75). Size range – ABL = 1.95 mm to 3.74 mm; with the attributes of the genus *Asklepia* as described by Liebke (1938). Adults with head and pronotum smooth and shiny; elytron quadrangular, apically truncated, margin slightly oblique, pattern coloration with pale maculae (slightly flavotestaceous in some individuals); labial palpomeres pubescent, ultimate article globose basally and subulate apically; legs testaceous; basal antennomeres fulvous, medial antennomeres infuscated, apical antennomeres white (the number of basal, medial, and apical antennomeres is described for each species below). Abdominal sterna sparsely pubescent.

Species are arrayed across three species groups based mainly on the armature of the male aedeagal endophallus: *geminata* species group (endophallus without spines, Fig. 57), *hilaris* species group (endophallus with multiple spines, Figs 58–63), and *pulchripennis* group (endophallus with two spines, Figs 64–74). In addition, the species of the *geminata* species group have elytral striae and moderately convex intervals; the *hilaris* group members have explanate lateral margins on the pronotum while those of the *pulchripennis* groups are feebly beaded only in the anterior half.

##### Dispersal potential.

The wings are fully developed in most individuals we have studied, thus it is likely that these beetles are moderate to strong flyers; however, in at least two species there are also brachypterous adults. This is unusual in lowland Amazonian species; for example, see Adis et al. (1997 [1998]) on a species of an odacanthine near Manaus, Brazil.

**Geographic**

##### distribution.

(Figs 76–78). As currently known, the range of this genus extends in cis-Andean South America from southeastern Colombia south to Bolivia and east to Guyana and Belém, Brazil, and from there south to Entre Ríos Province, Argentina.

##### Way of life.

These species live close to water in wet leaf litter and on aquatic vegetation (macrophytes) of backwashes along rivers, streams, and lakeshores of both Varzea and Igapó forests and among dead leaf accumulations on rocky or sandy stream banks. Immature stages are unknown; however, given the wide variation in adult size within a species as noted in the introduction, it is possible that larvae are ectoparasitoids (cf. Erwin 1967, 1979; Frank et al. 2009).

##### Notes.

Not much has been previously published on this genus. We now know there are many undescribed species across the Amazon Basin and on the Guiana Shield and the southern part of the Brazilian Shield; hence it is not monobasic as reported by Reichardt (1974). Bates (1871) actually described four species (assigned herein to *Asklepia*) placing them in the genus *Eucaerus* LeConte, none of which were recognized previously as an *Asklepia* species (cf. Liebke 1938; Reichardt 1974). Since the genus was in need of taxonomic revision, we have provided it here. Many areas of South America have not been sampled for very small water-side beetles; thus, it is likely many more species of this genus will be discovered in the future. As pointed out recently (Erwin et al. 2010), many very tiny-sized carabid clades have gone mostly unnoticed. In the 1930’s, the discovery of *Gehringia olympica* Darlington served to adjust the search image of carabidologists toward a fractal universe smaller than previously used; however, such search image needs to be focused more in the rainforests of the world.

##### Included species.

The species list below, as well as arrangement of descriptions that follows, is ordered alphabetically.

##### *geminata* species group taxa

*Asklepia geminata* (Bates), 1871:78, new combination, Brazil, Perú

##### *hilaris* species group taxa

*Asklepia campbellorum* Zamorano & Erwin, sp. n., Brazil

*Asklepia demiti* Erwin & Zamorano, sp. n., Brazil

*Asklepia duofos* Zamorano & Erwin, sp. n., Brazil

*Asklepia grammechrysea* Zamorano & Erwin, sp. n., Perú

*Asklepia hilaris* (Bates), 1871:79, comb. n., Brazil

*Asklepia laetitia* Zamorano & Erwin, sp. n., Colombia

*Asklepia lebioides* (Bates), 1871:79, comb. n., Brazil

*Asklepia matomena* Zamorano & Erwin, sp. n., Brazil

##### *pulchripennis* species group taxa

*Asklepia adisi* Erwin & Zamorano, sp. n., Brazil, Perú

*Asklepia asuncionensis* Erwin & Zamorano, sp. n., Paraguay

*Asklepia biolat* Erwin & Zamorano, sp. n., Perú

*Asklepia bracheia* Zamorano & Erwin, sp. n., Perú

*Asklepia cuiabaensis* Erwin & Zamorano, sp. n., Brazil

*Asklepia ecuadoriana* Erwin & Zamorano, sp. n., Ecuador

*Asklepia kathleenae* Erwin & Zamorano, sp. n., Brazil

*Asklepia macrops* Erwin & Zamorano, sp. n., Argentina

*Asklepia marchantaria* Erwin & Zamorano, sp. n., Brazil

*Asklepia marituba* Zamorano & Erwin, sp. n., Brazil

*Asklepia pakitza* Erwin & Zamorano, sp. n., Perú

*Asklepia paraguayensis* Zamorano & Erwin, sp. n., Paraguay

*Asklepia pulchripennis* (Bates), 1871:79, comb. n., Brazil

*Asklepia samiriaensis* Zamorano & Erwin, sp. n., Perú

*Asklepia stalametlitos* Zamorano & Erwin, sp. n., Bolivia

*Asklepia strandi* Liebke, 1938:113 Guyana

*Asklepia surinamensis* Zamorano & Erwin, sp. n., Surinam

*Asklepia vigilante* Erwin & Zamorano, sp. n., Perú

##### Key to the species of *Asklepia* Liebke, 1938

**Note.** Because of the variability within species and the marked similarity across some species, only features of the male genitalia provide reliable means for identification of some species. Unfortunately, we did not have at our disposal males of all the species to image.

**Table:** 

1	Elytron markedly striate, intervals moderately convex (*geminata* species group) (Habitus, Fig. 29)	*Asklepia geminata* (Bates)
1’	Elytron devoid of striae, intervals and interneurs effaced from surface (although inconsistently spaced serial rows of interneur micropunctures can be seen through the transparent cuticle in some species and on the surface of others)	2
2(1’)	Pronotum with lateral margin narrowly explanate (*hilaris* species group)	3
2’	Pronotum with lateral margin effaced posterior to medial lateral seta except just slightly anterior to hind angle — there shortly beaded; some species slightly beaded from anterior angle to medial lateral seta but not explanate (*pulchripennis* species group)	9
3(2)	Antennal scape and pedicel testaceous, antennomeres 3–7 deeply infuscated, antennomeres 8–11 white	4
3’	Antennal scape and pedicel testaceous, antennomeres 3–6 deeply infuscated, antennomere 7 bicolored (Habitus, Fig. 32)	*Asklepia duofos* sp. n.
4(3)	Pronotum completely infuscated or piceous (rarely median anterior margin slightly paler)	5
4’	Pronotum infuscated or piceous only laterally, disc concolorous with pale elytral maculae (Habitus, Fig. 33)	*Asklepia grammechrysea* sp. n.
5(4)	Dorsal surface largely dark, elytral maculae if present very small	6
5’	Dorsal surface with dark fore body and large elytral maculae	7
6(5)	Pronotum markedly constricted in basal third, markedly cordate. Mostly size larger (ABL = 3.2–3.83 mm; SBL = 2.18–3.20 mm; TW = 1.10–1.82 mm) (Habitus, Fig. 30)	*Asklepia campbellorum* sp. n.
6’	Pronotum much less constricted in basal third, tapered to base. Mostly size smaller (ABL = 2.47–2.96 mm; SBL = 2.2–2.5 mm; TW = 1.15–1.45 mm) (Habitus, Fig. 37)	*Asklepia matomena* sp. n.
7(5’)	Pronotum longer and narrow (W/L ratio = 1.331–1.550)	8
7’	Pronotum short and wide (W/L ratio = 1.686–1.960), proportionally large, as wide as head across eyes (Habitus, Fig. 36)	*Asklepia lebioides* (Bates)
7’’	Pronotum short and wide (W/L ratio = 1.686–1.960), proportionally small, not as wide as head across eyes (Habitus, Fig. 34)	*Asklepia hilaris* (Bates)
8(7)	Elytron with anterior macula very extensive; interneurs well impressed and striatopunctate (Habitus, Fig. 35)	*Asklepia laetitia* sp. n.
8’	Elytron with anterior macula small, divided in some individuals into two spots; interneurs of a very fine series of minute punctulae (Habitus, Fig. 31)	*Asklepia demiti* sp. n.
9(2’)	Elytron with pale lateral margin restricted to apical half, mostly an extension inclusive of median macula in sector C	10
9’	Elytron with pale lateral margin extended nearly to humerus (Habitus, Fig. 53)	*Asklepia strandi* Liebke
10(9)	Antenna mostly pale, only antennomeres 4, 5, and 6 darkly infuscated	11
10’	Antenna mostly infuscated, only antennomeres 1, 2, and 8–11 completely pale	12
11(10)	Head infuscated	13
11’	Head pale, flavous	14
12(10’)	Antennomere 7 bicolored, base infuscated, apex white	15
12’	Antennomere 7 entirely white	21
13(11)	Pronotum wider than long (W/L ratio = 1.340–1.385 (Habitus, Fig. 54))	*Asklepia surinamensis* sp. n.
13’	Pronotum nearly as wide as long (W/L ratio = 1.154 - 1.314) (Habitus, Fig. 38)	*Asklepia adisi* sp. n.
14(11’)	Mostly size larger (ABL = 3.25 mm; SBL = 3.054 mm; MW = 1.648 mm). Eyes very large, anterior/posterior diameter markedly greater than length of antennomere 3. Elytra across humeri narrower than across apical third. Antennomere 3 testaceous (Habitus, Fig. 45)	*Asklepia macrops* sp. n.
14’	Mostly size smaller (ABL = 2.72 mm; SBL = 2.477 mm; MW = 1.27 mm). Eyes normal for genus, anterior/posterior diameter about equal in length to that of antennomere 3. Elytra across humeri about equal to that at apical third. Antennomere 3 slightly infuscated (Habitus, Fig. 39)	*Asklepia asuncionensis* sp. n.
15(12)	Elytra markedly shiny, microsculpture effaced	16
15’	Elytra matte, microsculpture well developed, of isodiametric sculpticells (Habitus, Fig. 51)	*Asklepia samiriaensis* sp. n.
16(15)	Head and pronotum markedly contrasting, head much darker in color than pronotum	17
16’	Head and pronotum concolorous	18
17(16)	Head and pronotum flavous. Pronotum much wider than long (W/L ratio = 1.726) (Habitus, Fig. 42)	*Asklepia cuiabaensis* sp. n.
17’	Head and pronotum aurantiacus. Pronotum slightly wider than long (W/L ratio = 1.308–1.370) (Habitus, Fig. 48)	*Asklepia pakitza* sp. n.
18(16’)	Head and pronotum pale, flavous, or aurantiacus	19
18’	Head and pronotum infuscated (Habitus, Fig. 43)	*Asklepia ecuadoriana* sp. n.
19(18)	Pronotum with beaded lateral margin. Elytron with large macula of sectors A, B, and C markedly U-shaped (e.g., Fig. 55)	20
19’	Pronotum without beaded lateral margin. Elytron with large macula of sectors A, B, and C shallowly U-shaped in sector A (Habitus, Fig. 14)	*Asklepia biolat* sp. n.
20(19)	Macula of sectors E and F extended to lateral margin (Habitus, Fig. 55)	*Asklepia vigilante* sp. n.
20’	Macula of sectors E and F not extended to lateral margin (Habitus, Fig. 52)	*Asklepia stalametlitos* sp. n.
21(12’)	Head infuscated, pronotum pale	22
21’	Head and pronotum both pale, flavous or aurantiacus	23
22(21)	Size larger (ABL = 2.81 mm; SBL = 2.478 - 2.769 mm; TW = 1.264–1.506 mm). Elytra across humeri square, lateral margins parallel; all available specimens alate (Habitus, Fig. 49)	*Asklepia paraguayensis* sp. n.
22’	Size smaller (ABL = 2.26 mm; SBL = 2.167 mm; TW = 1.241 mm). Elytra across humeri narrow, lateral margins rounded, sloped to humerus; both available specimens brachypterous (Habitus, Fig. 44)	*Asklepia kathleenae* sp. n.
23(21’)	Elytra markedly short and convex (LE/LP ratio = 2.72–2.79)	24
23’	Elytra of normal length for genus (LE/LP ratio = 2.84–2.96)	25
24(23)	Pronotum narrow, its width less than width across eyes (Habitus, Fig. 41)	*Asklepia bracheia* sp. n.
24’	Pronotum broad, its width equal to width across eyes (Habitus, Fig. 47)	*Asklepia marituba* sp. n.
25(23’)	Pronotum globose, markedly convex and broad; frons markedly convex. Elytron with apex broadly infuscated (Habitus, Fig. 46)	*Asklepia marchantaria* sp. n.
25’	Pronotum moderately convex, narrow; frons barely convex. Elytron with apex completely pale (Habitus, Fig. 50)	*Asklepia pulchripennis* (Bates)

### *geminata* species group

This species group is monobasic and its single species is widespread from northern Perú to near the mouth of the Río Amazonas at Belém. Adults of the *geminata* species group are dark brown with markedly striate elytral interneurs, striae without punctures, intervals moderately convex; prothorax with narrowly explanate lateral margins and small, slightly produced obtuse hind angles. Endophallus of aedeagus without spines.

#### Asklepia geminata

Taxon classificationAnimaliaColeopteraCarabidae

(Bates, 1871) comb. n.

**Figures 29–32.**
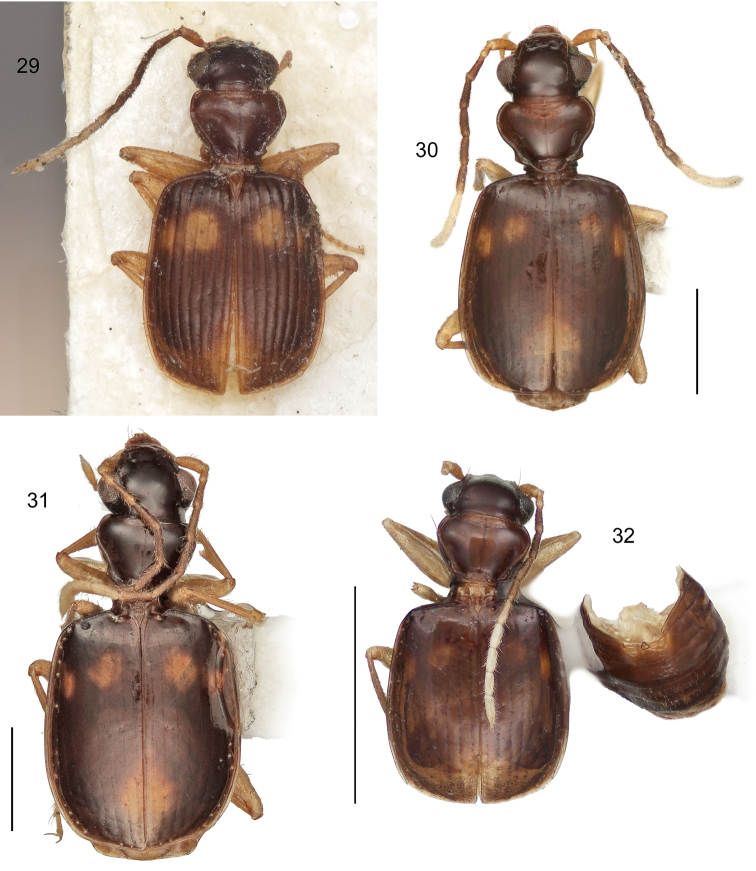
Digital Photo-illustrations, habitus, dorsal aspect of holotypes. **29** *Asklepia geminata* (Bates, 1871) ADP132513, Santarém, Río Tapajos, Brazil **30** *Asklepia campbellorum* Zamorano & Erwin, sp. n. ADP133032, 20 km SW Manaus, Brazil **31** *Asklepia demiti* Erwin & Zamorano, sp. n. ADP132539, Río Demiti, Brazil. Note that the holotype has feebly infuscated antennomeres 3-7, others in the series have more normal infuscation, as in other species with this attribute **32** *Asklepia duofos* Zamorano & Erwin, sp. n. ADP133147, 20 km SW Manaus, Brazil. Scale line = 1 mm.

Twin-spot pattern-wing beetle Figs 29 , 57 , 76

Eucaerus geminatus Bates, 1871:78.

##### Holotype.

**Brazil**, Pará, Santarém, Río Tapajos, 2.4079°S, 54.7969°W, 30m, (H.W.Bates)(MNHP: ADP132513, male). Specimen labeled “Holotype” by George E. Ball in 1972.

##### Derivation of specific epithet.

The specific epithet, *geminata*, is a Latin adjective, meaning double (for example, the celestial Gemini, sign of the twins, Romulus and Remus, the mythological founders of Rome), or arranged in pairs as used here by Bates with reference to the two elytral spots.

##### Proposed english vernacular name.

Twin-spot pattern-wing beetle.

##### Diagnosis.

With the attributes of the genus *Asklepia* as described by Liebke (1938) and as noted above under the generic diagnosis, and medium-sized for the genus (SBL = 2.512–2.794 mm). Adult with head and prothorax brunneus, elytral maculae fulvous or aurantiacus in some individuals; elytron brunneus with an elongated oval, horizontally oriented macula crossing basal quadrants and a round flavous macula in the apical proximal quadrant, lateral margin of medial and apical quadrant fulvous; metasternum, abdominal sterna III-VI, and epipleuron brunneus, abdominal sternum VII slightly paler; legs flavotestaceous; antennal scape and pedicel testaceous, antennomeres 3-6 and basal half of 7 deeply infuscated, apical half of 7, 8-11 white. Head and pronotum surface devoid of microsculpture surface luster very shiny; elytra surface with finely impressed isodiametric microsculpture. Pronotum cordiform, narrowly explanate, with medial lobe at base, lateral margin beaded; hind angle moderately prominent; median line well defined. Elytral interneurs striate and continuous along entire length of elytron.

##### Description.

(Figs 29, 57). ***Habitus*:** (Fig. 29). ***Size*:** [See also Table 1] Medium-size for the genus; ABL = 2.768–2.997 mm, SBL = 2.512–2.794 mm, TW (total width) 1.411–1.440 mm, LP = 0.536–0.603 mm, WP = 0.733–0.820 mm, LE = 1.622–1.849 mm. ***Color*:** See diagnosis above. *Luster*: See diagnosis above. ***Head*** (Fig. 29): as in description for genus above. ***Prothorax*.** Pronotum (Fig. 29) moderately broad, as wide as head across eyes (WH/WP, mean both sexes: 0.964), longer than head (LP/LH, mean both sexes: 1.641), wider than long (W/L, mean both sexes: 1.364); markedly cordiform and explanate, lateral margin beaded with seta at anterior third; anterior angles feebly produced; base markedly constricted with medial lobe at base; hind angle moderately produced and setose; median line well defined, transverse impression punctate, punctures infuscated; surface smooth throughout. ***Pterothorax*.** Normal for genus, see description for genus above. Elytra slightly convex; at apical third twice as long as head across eyes (WH/TW, mean both sexes: 0.524) and pronotum (WP/TW, mean both sexes: 0.544), longer than wide. Elytral interneurs striate and continuous along length of entire elytron. Surface with finely impressed microsculpture, sculpticells isodiametric. Hind wings fully developed. ***Legs*.** Overall, normal for genus, see description for genus above. ***Abdominal sterna*.** Overall, normal for genus, see description for genus above. ***Male genitalia*** (Fig. 57, see Fig. 61 for attribute labels). Median lobe with phallobase short about a fourth the length of shaft, basal opening moderately small, oriented parallel to shaft. Shaft narrow, moderately curved ventrally, dorsally sclerotized except for short ostium; in ventral aspect tapered toward rather narrowly rounded apex, in lateral aspect, a rounded apex. Parameres: left very large and broad, right small and triangular, apex of left paramere lobate much longer than right paramere about half the length of shaft (measured in left lateral aspect). Endophallus without preapical spines. ***Female genitalia*.** Not investigated, presumably similar to that of *Asklepia demiti* sp. n.

##### Dispersal potential.

These beetles are macropterous and probably capable of flight. They are moderately swift and agile runners.

##### Distribution.

(Fig. 76). The wide geographical range from near the mouth of the Río Amazon at Belém to the black-waters of Pacaya-Samiria Reserve in Perú on the upper Amazon drainage system is unusual for this genus. But as pointed out in the introduction, very small carabid beetles are not collected by any but carabid specialists, and there have been few of those, working the Amazon Basin.

##### Way of life.

See Erwin (1991) for a general description of the genus. Adults of this species are active in both the late rainy season and transition to the dry season in lowland rain forest. They occur particularly in wet leaf litter in “aguajales,” i.e., palm forests (*Mauritia flexuosa* L. f.) that are present along major waterways throughout the Amazon Basin and along river and stream margins and in open marshes.

##### Other specimens examined.

**Brazil**, Pará, Belém, 5m, 1.46°S, 48.42°W, 8 October 1978, (G.E. Ball, K.E. Ball)(UASM: ADP132557, female), Santarém, Río Tapajos, 2.4079°S, 54.7969°W, 30m, no date, (CMNH: ADP133571. male). **Perú**, Loreto, Rio Samiria, Boca Caño Inglés Camp, 5.1317°S, 75.0617°W, 117m, 23 August 1991, (T.L. Erwin, G.E. Ball, D. Shpeley)(NMNH: ADP109186, male).

### *hilaris* species group

This species group contains eight species and is widespread from northern Perú to at least Santarém on the lower Río Amazonas, north into Venezuela and only known south of the Río Amazonas at Manaus and Santarém by a few kilometers. Dorsal surface mostly dark, particularly the head and pronotum and base color of the elytra; abdominal sterna infuscated except VII which is aurantiacus. Pronotum with lateral margin narrowly explanate; hind angles produced and about right or acute. Endophallus of aedeagus with 6–12 spines, depending on the species.

#### Asklepia campbellorum

Taxon classificationAnimaliaColeopteraCarabidae

Zamorano & Erwin sp. n.

http://zoobank.org/EDA34F46-B81B-4D4E-8CF4-ECD7EFEE39F9

Campbells’ pattern-wing beetle Figs 30 , 58 , 77

##### Holotype.

**Brazil**, Amazonas, 20 km SW Manaus, 3.166°S, 60.234°W, 47m, 6 November 1969 (J.M. Campbell, B.A. Campbell)(NMNH: ADP133032, male).

##### Derivation of specific epithet.

The specific epithet, *campbellorum*, is an eponym based on the family name of Milt and Beverly Campbell**^†^**, collectors of the type series.

##### Proposed english vernacular name.

Campbells’ pattern-wing beetle.

##### Diagnosis.

With the attributes of the genus *Asklepia* as described by Liebke (1938) and as noted above under the generic diagnosis, and medium to large-sized for the genus (SBL = 2.941–3.198 mm). Adults with head and prothorax brunneus, elytral maculae aurantiacus; elytron fuscous with a small rounded aurantiacus macula in the lateral apical quadrant and proximal apical quadrant and an oval aurantiacus macula in the proximal apical quadrant, maculae reach the sutural area. Color pattern variable, maculae in the apical quadrants are connected, forming a single macula or completely absent in some individuals. Metasternum, abdominal sterna III-VI, and epipleuron brunneus, abdominal sternum VII paler; legs testaceous; antennal scape and pedicel testaceous, antennomeres 3-7 deeply infuscated, 8-11 white. Dorsal surface devoid of microsculpture, surface luster very shiny. Pronotum cordiform, narrowly explanate, with medial lobe at base, lateral margin beaded; anterior angles markedly produced, hind angle angulate, very prominent; median line moderately defined. Elytral interneurs evident as rows of continuous closely spaced fine punctures.

##### Description.

(Figs 30, 58). ***Habitus*:** (Fig. 30). ***Size*:** [See also Table 2] Small-size to large-size for the genus; ABL = 3.17–3.61 mm, SBL = 2.182–3.198 mm, TW (total width) 1.106–1.824 mm, LP = 0.467–0.691 mm, WP = 0.611–0.956 mm, LE = 1.386–2.069 mm. ***Color*:** See diagnosis above. ***Luster*:** See diagnosis above. ***Head*** (Fig. 30): as in description for genus above. ***Prothorax*.** Pronotum (Fig. 30) moderately broad, slightly wider than head across eyes (WH/WP, both sexes: 0.966), longer than head (LP/LH, mean both sexes: 1.414), wider than long (W/L, mean both sexes: 1.395); markedly cordiform and explanate, lateral margin beaded with seta at anterior third; base markedly constricted and lobed medially; anterior angles markedly produced, hind angle markedly prominent, produced and setose; median line moderately defined, apical transverse impression punctate, punctures infuscated; surface smooth throughout. ***Pterothorax*.** Normal for genus, see description for genus above. Elytra slightly convex; at apical third twice as wide as head across eyes (WH/TW, mean both sexes: 0.492) and pronotum (WP/TW, mean both sexes: 0.510). Elytral interneurs evident as rows of continuous closely spaced fine punctures; punctures homogeneous. Hind wings fully developed. ***Legs*.** Overall, normal for genus, see description for genus above. ***Abdominal sterna*.** Overall, normal for genus, see description for genus above. ***Male genitalia*** (Fig. 58, see Fig. 61 for attribute labels). Median lobe with phallobase short about a fourth the length of shaft, basal opening small, oriented parallel to shaft. Shaft broad, slightly curved ventrally, dorsally sclerotized except for short ostium; in ventral aspect tapered toward rather narrowly acute apex, in lateral aspect, a rounded apex. Parameres: left very large and broad, right small and triangular; apex of left paramere lobate much longer than right paramere about half the length of shaft (measured in left lateral aspect). Endophallus with 7 preapical spines. ***Female genitalia*.** Not investigated, presumably similar to that of *Asklepia demiti* sp. n.

##### Dispersal potential.

These beetles are macropterous and probably capable of flight. They are moderately swift and agile runners.

##### Distribution.

(Fig. 77). This species has been found at only one location on the shore of a small lake near the middle of the Amazon River drainage system. But that does not at all indicate its actual distribution: As has been pointed out above, very small beetles are inadequately sampled, especially in the Neotropics.

##### Way of life.

See Erwin (1991) for a general description. Adults of this species are active in lowland rainforest in the transition from rainy to dry seasons.

##### Other specimens examined.

**Brazil**, Amazonas, 20 km SW Manaus, 3.166°S, 60.234°W, 47m, 6 November 1969 (J.M. Campbell, B.A. Campbell)(NMNH: ADP132693, ADP133141, ADP133167, ADP132734, ADP133165, ADP133155, ADP132705, ADP133064, ADP109196, female paratypes, ADP133177, ADP132685, ADP133113, ADP133127, ADP133157, ADP132727, ADP133119, ADP133010, ADP133004, ADP133135, ADP132723, ADP133137, ADP133191, ADP133147, male paratypes), (ADP133137, male, forebody missing).

#### Asklepia demiti

Taxon classificationAnimaliaColeopteraCarabidae

Erwin & Zamorano sp. n.

http://zoobank.org/A36C1689-CB6E-450D-81E0-18B6027A7B1E

Demiti pattern-wing beetle Figs 31 , 59 , 75 , 77

##### Holotype.

**Brazil**, Amazonas, circa Rio Demiti, 0.5748°N, 66.6869°W, 116m, 13 September 1978 (G.E Ball, K.E. Ball) (NMNH: ADP132539, male).

##### Derivation of specific epithet.

The specific epithet, *demiti*, is a singular Latinized feminine noun in apposition, based on the name of the river along which these beetles are found.

##### Proposed english vernacular name.

Río Demiti pattern-wing beetles.

##### Diagnosis.

With the attributes of the genus *Asklepia* as described by Liebke (1938) and as noted above under the generic diagnosis, and medium to large-sized for the genus (SBL = 2.590–3.131 mm). Adults with head and prothorax fuscous, elytral maculae fulvous or aurantiacus in some individuals; elytron fuscous with rounded aurantiacus macula in the lower left corner of apical proximal quadrant and in the upper right corner of medial lateral quadrant; maculae are connected forming a single macula in some individuals, apical proximal quadrant with a rounded aurantiacus macula in the upper left corner and sutural area aurantiacus; metasternum, abdominal sterna III-VI, and epipleuron brunneus, abdominal sternum VII slightly paler; legs flavotestaceous; antennal scape and pedicel testaceous, antennomeres 3-7 infuscated (less so in the holotype), 8-11 white. Dorsal surface devoid of microsculpture, surface luster very shiny. Pronotum cordiform, narrowly explanate, with medial lobe at base, lateral margin beaded; anterior angle feebly produced, hind angle angulate, very prominent; median line moderately defined. Elytral interneurs evident as short discontinuous rows of fine punctures.

##### Description.

(Figs 31, 59, 75). ***Habitus*:** (Fig. 31). ***Size*:** [See also Table 3] Medium to large-size for the genus; ABL = 2.089–3.071 mm, SBL = 2.590–3.131 mm, TW (total width) 1.491–1.815 mm, LP = 0.545–0.642 mm, WP = 0.762–0.888 mm, LE = 1.721–2.036 mm. ***Color*:** See diagnosis above. ***Luster*:** See diagnosis above. ***Head*** (Fig. 31): as in description for genus above. ***Prothorax.*** Pronotum (Fig. 31) moderately broad, as wide as head across eyes (WP/WH, both sexes: 1.025), longer than head (LP/LH, mean both sexes: 1.471), wider than long (W/L, mean both sexes: 1.380); markedly cordiform and explanate, lateral margin beaded with seta at anterior third; apical margin straight, base markedly constricted with medial lobe at base; anterior angle feebly produced, hind angle markedly prominent and setose; median line moderately defined, apical transverse impression punctate, punctures coarse and infuscated; surface smooth throughout. ***Pterothorax.*** Normal for genus, see description for genus above. Elytra slightly convex; at apical third twice as wide as head across eyes (WH/TW, mean both sexes: 0.492) and pronotum (WP/TW, mean both sexes: 0.504). Elytral interneurs evident as short discontinuous rows of fine punctures; punctures with a fuscous halo at basal and apical proximal quadrant of elytron. Hind wings fully developed. ***Legs*.** Overall, normal for genus, see description for genus above. ***Abdominal sterna*.** Overall, normal for genus, see description for genus above. ***Male genitalia*** (Fig. 59, see Fig. 61 for attribute labels). Median lobe with phallobase short about a fifth the length of shaft, basal opening small, oriented parallel to shaft. Shaft broad, slightly curved ventrally, dorsally sclerotized except for short ostium; in ventral aspect tapered toward rather narrowly rounded apex, in lateral aspect, a broadly rounded apex. Left paramere very large and broad, right small and triangular, apex of left paramere lobate much longer than right paramere about half the length of shaft (measured in left lateral aspect). Endophallus with 10 small medial spines and one large distal spine. ***Female genitalia*.** (Fig. 75A and B) Ovipositor with broad laterotergite (**lt**) and two narrow gonocoxites (**gc 1, gc 2**); gonocoxite 1 apico-laterally not setose; gonocoxite 2 shallowly falcate, base (**b**) medium-size much broader than narrow blade (**bl**) which is elongate, with two dorsal ensiform setae (**des**), ventral ensiform seta absent, ensiform setae moderately short and robust; without ventral preapical nematiform setae. Reproductive tract proximally with moderately short, broad bursa copulatrix (**bc**), continuous at its distal end with common oviduct (**co**) and long robust bipartite spermatheca (**sp**) distal to broad short villous canal (**vc**), one lobe slightly narrowed distally; spermathecal gland not found in dissection; spermathecal gland duct (**sgd**) robust, heavily sclerotized, attached to oviduct at base of its broadened portion. Defense gland (Fig. 75C) with an annulated sausage-shaped accessory gland (**cc**) and large reservoir (**gldr**) distal to a long efferent duct (**ed**).

##### Dispersal potential.

These beetles are macropterous and probably capable of flight. They are moderately swift and agile runners.

##### Distribution.

(Fig. 77). This species has been found at only two locations on second-order white-water streams of the Río Negro drainage system. But that does not at all indicate its real distribution: as has been pointed out above, very small beetles are inadequately sampled, especially in the Neotropics.

##### Way of life.

See Erwin (1991) for a general description of the genus. Adults of this species are active in lowland Varzea rainforest in the late rainy season. It seems from the known samples that this species is found on white-water systems.

##### Other specimens examined.

**Brazil**, Amazonas, circa Rio Demiti, 0.5748°N, 66.6869°W, 116m, 13 September 1978 (G.E Ball, K.E. Ball)(NMNH: ADP132585, female paratype, ADP132483, ADP132501, male paratypes). **Venezuela**, Amazonas, 29 km S Puerto Ayacucho, Río Paria Chico, 5.4694N, 67.6029W, 71m, (J.T. Polhemus)(NMNH: ADP132605, male paratype).

#### Asklepia duofos

Taxon classificationAnimaliaColeopteraCarabidae

Zamorano & Erwin sp. n.

http://zoobank.org/45600768-3727-45A2-BDD8-F1C07ACA6CD8

Two-lights pattern-wing beetle Figs 32 , 77

##### Holotype.

**Brazil**, Amazonas, 20 km SW Manaus, 3.166°S, 60.234°W, 47m, 6 November 1969 (J.M. Campbell, B.A. Campbell)(NMNH: ADP132555, male).

##### Derivation of specific epithet.

The specific epithet, derived from the Greek *duofos*, δυο (duo) = two, fɸσ (fos) = lights) is a noun used in apposition referring to the two bright spots on the elytra.

##### Proposed english vernacular name.

Two-lights pattern-wing beetle.

##### Diagnosis.

With the attributes of the genus *Asklepia* as described by Liebke (1938) and as noted above under the generic diagnosis, and small to medium-sized for the genus (SBL = 2.248–2.941 mm). Adults with head and prothorax brunneus, elytral maculae aurantiacus; elytron fuscous with small rounded aurantiacus macula in the lower left corner of proximal basal quadrant, a small rounded aurantiacus macula in the upper left corner of proximal basal quadrant, maculae about the size of ¼ of the quadrant, a semicircular aurantiacus macula in the proximal apical quadrant, macula does not reach the apical margin; proximal quadrants slightly paler compared with lateral quadrants; metasternum, abdominal sterna III-VI, and epipleuron brunneus, abdominal sternum VII paler; legs testaceous; antennal scape and pedicel testaceous, antennomeres 3-6 and basal half of 7 deeply infuscated, apical half of 7, 8-11 white. Dorsal surface devoid of microsculpture, surface luster very shiny. Pronotum cordiform, narrowly explanate, lateral margin beaded; anterior angles feebly produced, hind angle markedly prominent; median line moderately defined. Elytral interneurs evident as short discontinuous rows of widely spaced coarse punctures.

##### Description.

***Habitus*** (Fig. 32). ***Size*:** [See also Table 4] Small-sized for the genus; ABL = 2.433 mm, SBL = 2.248 mm, TW (total width) 1.124 mm, LP = 0.495 mm, WP = 0.666 mm, LE = 1.407 mm. ***Color*:** See diagnosis above. ***Luster*:** See diagnosis above. ***Head*** (Fig. 32): as in description for genus above. ***Prothorax*.** Pronotum (Fig. 32) moderately broad, as wide as head across eyes (WH/WP: 0.939), longer than head (LP/LH: 1.423), wider than long (PW/PL: 1.397); markedly cordiform and explanate, lateral margin beaded with seta at anterior third; base markedly constricted; anterior angles feebly produced, hind angle markedly prominent, produced and setose; median line markedly defined, apical transverse impressions punctate, punctures infuscated; surface smooth throughout. ***Pterothorax*.** Normal for genus, see description for genus above. Elytra slightly convex; at apical third twice as wide as head across eyes (WH/MW: 0.489) and pronotum (WP/MW: 0.521), longer than wide. Elytral interneurs evident as short rows of discontinuous widely spaced coarse punctures. Hind wings fully developed. ***Legs*.** Overall, normal for genus, see description for genus above. ***Abdominal sterna*.** Overall, normal for genus, see description for genus above. ***Male genitalia*.** The male paratype was dissected for illustrating the male aedeagus; however, it was damaged in the process and we were hesitant to dissect the holotype at this time. We do note the presence of multiple spines on the endophallus. ***Female genitalia*.** Not investigated, presumably similar to that of *Asklepia demiti* sp. n.

##### Dispersal potential.

These beetles are macropterous and probably capable of flight. They are moderately swift and agile runners.

##### Distribution.

(Fig. 77). This species has been found at only one location on the shore of a small lake near the middle Amazon River drainage system. But that does not at all indicate its real distribution: as has been pointed out above, very small beetles are inadequately sampled, especially in the Neotropics.

##### Way of life.

See Erwin (1991) for a general description. Adults of this species are active in lowland rainforest during the transition from rainy to dry seasons

##### Other specimens examined.

None.

#### Asklepia grammechrysea

Taxon classificationAnimaliaColeopteraCarabidae

Zamorano & Erwin sp. n.

http://zoobank.org/26ADBF2A-0A81-4009-A1F2-54FB25AC978F

**Figure 33–36.**
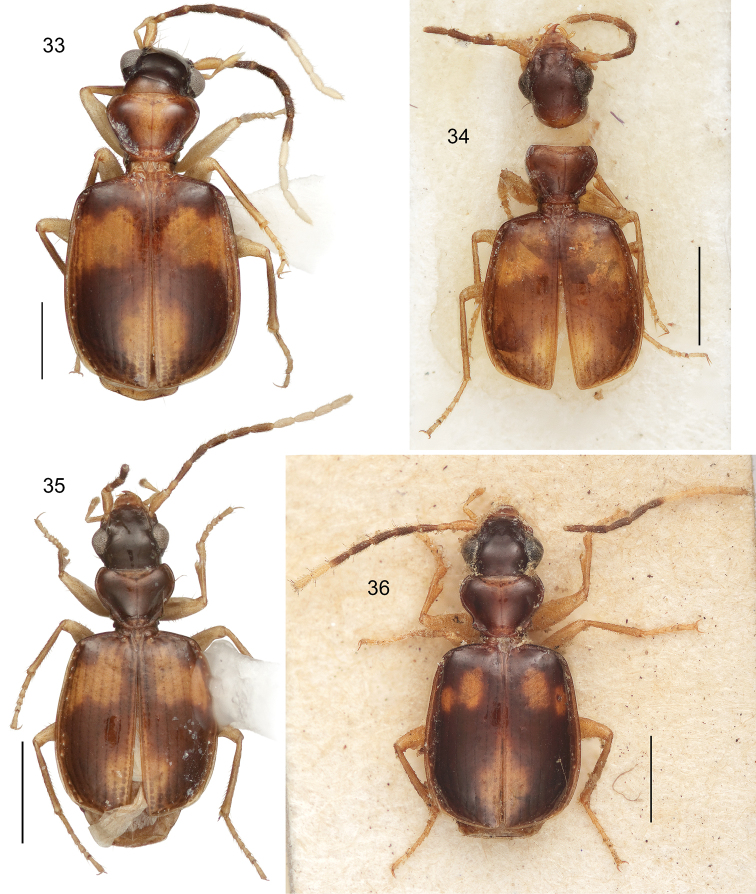
Digital Photo-illustrations, habitus, dorsal aspect of holotypes. **33** *Asklepia grammechrysea* Zamorano & Erwin, sp. n. ADP133151, Pithecia, Perú **34** *Asklepia hilaris* (Bates, 1871), comb. n., Brazil ADP132543, São Paulo de Olivença, Brazil **35** *Asklepia laetitia* Zamorano & Erwin, sp. n. ADP109190, Leticia, Colombia **36** *Asklepia lebioides* (Bates, 1871), comb. n., ADP109194, Santarém, Río Tapajos, Brazil. Scale line = 1 mm.

Golden-line pattern-wing beetle Figs 33 , 60 , 77

##### Holotype.

**Perú**, LORETO, circa Pithecia, Cocha Shinguito, 5.1757°S, 74.655°W, 111m, 16 August 1989 (T.L. Erwin, G.P. Servat)(MUSM: ADP133151).

##### Derivation of specific epithet.

The specific epithet, *grammechrysea* derived from the Greek γραμμή (grammae) = line, κριχισηα (chrysea) = golden), is a noun in apposition referring to the yellow (flavous) line on the pronotum.

##### Proposed english vernacular name.

Golden-line pattern-wing beetle.

##### Diagnosis.

With the attributes of the genus *Asklepia* as described by Liebke (1938) and as noted above under the generic diagnosis. Individuals of this species present a wide range of sizes from small to large for the genus (SBL = 2.265–3.736 mm). Adults with head fuscous, pronotum bicolored, its lateral areas fuscous, medial area aurantiacus; elytral maculae aurantiacus, elytron fuscous with an elongated horizontally oriented flavous macula crossing basal lateral and proximal quadrant, sutural area fuscous, and with a semicircular flavous macula in the apical proximal quadrant, sutural area fulvous, lateral margin of medial and apical quadrants fulvous; metasternum, abdominal sterna III-VI, basal half of sternum VII and epipleuron brunneus, apical half of abdominal sternum VII paler; legs fulvous; antennal scape and pedicel testaceous, antennomeres 3-7 deeply infuscated, 8-11 white. Dorsal surface devoid of microsculpture, surface luster very shiny. Pronotum cordiform, narrowly explanate, with medial lobe at base, apical margin slightly concave lateral margin beaded; hind angle markedly prominent; median line markedly defined. Elytral interneurs evident as continuous rows of fine closely spaced punctures; punctures fuscous; basal and apical punctures each with infuscated halo.

##### Description.

(Figs 33, 60). ***Habitus*:** (Fig. 33). ***Size*:** [See also Table 5] Small to large-sized for the genus; ABL = 2.336–3.78 mm, SBL = 2.265–3.736 mm, TW (total width) 1.382–2.216 mm, LP = 0.481–1.016 mm, WP = 0.659–1.065 mm, LE = 1.440–2.381 mm. ***Color*:** See diagnosis above. ***Luster*:** See diagnosis above. ***Head*** (Fig. 33): as in description for genus above. ***Prothorax*.** Pronotum (Fig. 33) moderately broad, as wide as head across eyes (WH/WP, mean both sexes: 1.002), larger than head (LP/LH, mean both sexes: 1.512), about as wider than long (WP/LP, mean both sexes: 1.381); markedly cordiform and explanate, lateral margin beaded and fuscous with seta at anterior third; base markedly constricted and lobed medially; anterior angles moderately produced, hind angle markedly produced and setose; median line markedly defined, basal and apical transverse impressions punctate, punctures fuscous; surface smooth throughout.

***Pterothorax*.** Normal for genus, see description for genus above. Elytra slightly convex; at apical third twice as width as head across eyes (WH/TW, mean both sexes: 0.481) and pronotum (WP/TW, mean both sexes: 0.493), longer than wide. Elytral interneurs evident as continuous rows of fine closely spaced punctures; punctures each with a fuscous halo in the basal and apical proximal quadrant of elytron. Hind wings fully developed. ***Legs*.** Overall, normal for genus, see description for genus above. ***Abdominal sterna*.** Overall, normal for genus, see description for genus above. ***Male genitalia*** (Fig. 60, see Fig. 61 for attribute labels). Median lobe with phallobase short about a fourth the length of shaft, basal opening small, oriented parallel to shaft. Shaft broad, slightly curved ventrally, dorsally sclerotized except for short ostium; in ventral aspect tapered toward rather narrowly rounded apex, in lateral aspect, a rounded apex. Parameres: left very large and broad, right small and triangular; apex of left paramere lobate much longer than right paramere about half the length of shaft (measured in left lateral aspect). Endophallus with 7 preapical spines. ***Female genitalia*.** Not investigated, presumably similar to that of *Asklepia demiti* sp. n.

##### Dispersal potential.

These beetles are macropterous and probably capable of flight. They are moderately swift and agile runners.

##### Distribution.

(Fig. 77). This species has been found at locations on black-water systems across the northern and western areas of the Amazon River drainage system. But that does not at all indicate its real distribution: as has been pointed out above, very small beetles are inadequately sampled, especially in the Neotropics.

##### Way of life.

See Erwin (1991) for a general

##### Description.

Adults of this species are active in lowland Igapó rainforest during the rainy season. They have been found in wet leaf litter at the edge of small streams and lake shores as well as in old levee forests of *Attalea* palms near a black water river; they also occur on mud with grasses and among crumbly clods of yellowish clay at salt licks, as well as near rotting tree trunks at the water’s edge in low lying inundation forest at the edge of a black water lake and on sandy shorelines with matted rhizomes and dry leaf litter.

##### Other specimens examined.

**Perú**, Loreto, circa Pithecia, Cocha Shinguito, 5.1757°S, 74.655°W, 111m, 16 August 1989 (T.L. Erwin, G.P. Servat)(NMNH: ADP132980, ADP132709, ADP132741, ADP133197, ADP132717, ADP133161, ADP133163, ADP- 132739, ADP133187, ADP133053, ADP133185, ADP132697, ADP133123, ADP133101, ADP133099, ADP133111, ADP132978, ADP133143, ADP133153, ADP132465, ADP132537, ADP132591, ADP132471, ADP132533, ADP132579, ADP132517, ADP132522, ADP132559, ADP132497, ADP132526, ADP132511, ADP132573, ADP132459, female paratypes, ADP013271, ADP132753, ADP133183, ADP- 133173, ADP132721, ADP133107, ADP133095, ADP133060, ADP132703, ADP109204, ADP133129, ADP132715, ADP133139, ADP133117, ADP133175, ADP133179, ADP132683, ADP132725, ADP133079, ADP132735, ADP133072, ADP132747, ADP133105, ADP132730, ADP133125, ADP132488, ADP132462, ADP132479, ADP132516, ADP132461, ADP132498, ADP132504, ADP132514, ADP132486, ADP132502, ADP132577, ADP132472, ADP132473, ADP132567, ADP132599, male paratypes; Cocha Shinguito, south side, 5.19543°S, 74.640°W, 121m, 17 May 1990 (T.L. Erwin)(NMNH: ADP093477, ADP094131, ADP071242, ADP066673, ADP093475, ADP093474, ADP093453 ADP093457, ADP093431, ADP071517, female paratypes, ADP071518, ADP071516, ADP094133, ADP093458, ADP093430, ADP093456, ADP093436, male paratypes); 25 August 1991 (T.L. Erwin, M.G. Pogue)(NMNH: ADP071240, ADP071201, female paratypes, ADP071249, ADP071247, ADP- 071239, ADP071240, ADP071241, ADP071225, ADP071226, ADP071243, ADP071244, ADP071217, ADP071224, ADP071198, ADP071218, ADP071202, ADP071588, male paratypes); Cocha Shinguito, 5.1757°S, 74.655°W, 111m, 14 June 1990 (T.L. Erwin)(NMNH: ADP094139, female paratypes, ADP094109, ADP094109, ADP094117, ADP094111, ADP094135, ADP094136, ADP094140, male paratypes); 25 August 1991 (G.E. Ball, D. Shpeley)(NMNH: ADP132551, female paratype, ADP132593, ADP132563, male paratypes); Cocha Shinguito, 5.1746°S, 74.6581°W, 119m, 26 August 1991 (T.L. Erwin, M.G. Pogue)(NMNH: ADP071520, ADP071521, female paratypes, ADP071519, male paratype); Cocha Shinguito, margin of Río Samiria, 5.1867°S, 74.6187°W, 124m, 17 May 1990 (T.L. Erwin)(NMNH: ADP066600, ADP066669, female paratypes, ADP066581, ADP066597, ADP066625, ADP066647, male paratypes); Río Samiria, Camp Terry, 5.4883°S, 75.1927°W, 132m, 14 May 1990 (T.L. Erwin)(NMNH: ADP094434, ADP094362, ADP094318, ADP094374, ADP094323, ADP094322, ADP132528, ADP132464, ADP094321, female paratypes ADP094348, ADP094342, ADP094345, ADP094349, ADP094344, ADP094324, ADP132510, ADP094341, ADP094346, ADP132541, ADP132549, ADP094365, ADP132523, male paratypes); Hamburgo, Boca Caño Inglés Camp, 5.1317°S, 75.0617°W, 117m, 20 August 1991 (T.L. Erwin)(NMNH: ADP071046, female paratypes, ADP071633, male paratype); Explornapo Camp, Río Napo, Río Sucusari, 3.2494°S, 72.9205°W, 101m, 4 June 1992 (T.L. Erwin, E. Pfuno S., F. Pfuno S.)(NMNH: ADP023587, ADP023593, female paratypes, ADP023587, ADP091311, male paratypes); 14 June 1992 (T.L. Erwin)(NMNH: ADP052649, ADP052564, ADP052650, female paratypes, ADP052548, ADP052548, male paratypes); 19 June 1992 (T.L. Erwin)(NMNH: ADP009175, ADP009123, ADP052565, female paratypes, ADP009173, ADP052563, ADP009176, ADP009174, male paratypes); circa Explornapo Camp, Río Napo, Cocha Shimagai, 3.3563°S, 73.0467°W, 88m, 13 June 1992 (T.L. Erwin, E. Pfuno S., F. Pfuno S.)(NMNH: ADP008129, ADP008114, ADP008128, ADP094109, female paratypes, ADP008122, ADP008124, ADP008127, ADP008118, ADP008115, ADP008116, ADP008119, male paratypes).

#### Asklepia hilaris

Taxon classificationAnimaliaColeopteraCarabidae

(Bates, 1871) comb. n.

Cheerful pattern-wing beetle Figs 34 , 77

Eucaerus hilaris Bates, 1871:79.

##### Holotype.

**Brazil**, Amazonas, São Paulo de Olivença, 3.4622°S, 68.9499°W, 77m, (H.W. Bates)(BMNH: ADP132543, male). This specimen labeled “Holotype” by George E. Ball in 1972.

##### Derivation of specific epithet.

The specific epithet, *hilaris*, is a Latin adjective that adequately describes this species with gaily colorful elytra.

##### Proposed english vernacular name.

Cheerful pattern-wing beetle.

##### Diagnosis.

With the attributes of the genus *Asklepia* as described by Liebke (1938) and as noted above under the generic diagnosis, and small-sized for the genus (SBL = 2.336 mm). Adults with head and prothorax brunneus, elytral maculae fulvous or aurantiacus in some individuals; elytron brunneus with a rectangular flavous macula crossing basal lateral and proximal quadrants, macula not extended to lateral margin or sutural area, and a rounded flavous macula in the apical proximal quadrant, sutural area fulvous; metasternum, abdominal sterna III-VI, and epipleuron testaceous, abdominal sternum VII paler; legs testaceous; antennal scape and pedicel testaceous, antennomeres 3-6 deeply infuscated, 7-11 white. Dorsal surface devoid of microsculpture, surface luster very shiny. Pronotum cordiform, narrowly explanate; anterior angles feebly produced; lateral margin beaded; hind angle angulate, moderately prominent; median line moderately defined. Elytral interneurs evident as rows of discontinuous, coarse punctures widely spaced.

##### Description.

***Habitus*** (Fig. 34). ***Size*:** [See also Table 6] Small in size for the genus; ABL = 2.464 mm, SBL = 2.336 mm, TW (total width) 1.233 mm, LP = 0.483 mm, WP = 0.617 mm, LE = 1.495 mm. ***Color*:** See diagnosis above. ***Luster*:** See diagnosis above. ***Head*** (Fig. 34): as in description for genus above. ***Prothorax*.** Pronotum (Fig. 34) moderately broad, as wide as head across eyes (WH/WP: 1.085), longer than head (LP/LH: 1.346), wider than long (W/L: 1.294); markedly cordiform and explanate, lateral margin beaded with seta at anterior third; apical margin straight; base markedly constricted; hind angle moderately produced and setose; median line moderately well defined, apical transverse impression punctate, punctures infuscated; surface smooth throughout. ***Pterothorax*.** Normal for genus, see description for genus above. Elytra slightly convex; at apical third about same width as head across eyes (WH/TW: 1.099) and pronotum (WP/TW: 1.013). Elytral interneurs evident as rows of discontinuous, widely spaced, coarse punctures. Hind wings fully developed. ***Legs*.** Overall, normal for genus, see description for genus above. ***Abdominal sterna*.** Overall, normal for genus, see description for genus above. ***Male genitalia*.** Dissected by G.E. Ball in 1972 at the BMNH, but the genitalia are missing from the microvial pinned under the holotype. ***Female genitalia*.** Not investigated, presumably similar to that of *Asklepia demiti* sp. n.

##### Dispersal potential.

These beetles are macropterous and probably capable of flight. They are moderately swift and agile runners.

##### Distribution.

(Fig. 77). This species has been found at only one location on the white-water system of the Río Amazonas drainage system. But that does not at all indicate its real distribution: as has been pointed out above, very small beetles are inadequately sampled, especially in the Neotropics.

##### Way of life.

See Erwin (1991) for a general description of the genus. No way of life information is available for this species other than that they occur in lowland Amazonia along the “Rio Amazon.”

##### Other specimens examined.

None.

##### Note.

The holotype and only specimen seen by us is glued to a card and is somewhat damaged.

#### Asklepia laetitia

Taxon classificationAnimaliaColeopteraCarabidae

Zamorano & Erwin sp. n.

http://zoobank.org/628C9676-9823-4241-AA36-3226C8561E7E

Colombian pattern-wing beetle Figs 35 , 61 , 77

##### Holotype.

**Colombia**, Amazonas, Leticia, 4.1896°S, 69.9711°W, 72m, no date, (F.M. Oliveira, P. Wygodzinsky)(AMNH: ADP109190, male).

##### Derivation of specific epithet.

The specific epithet, *laetitia*, from the Latin greeting *laetitia* used in the nominative case, is a singular feminine noun in apposition, based on the name of the town near which these beetles were found, and meaning happiness, joy, gladness, and delight.

##### Proposed english vernacular name.

Colombian pattern-wing beetle.

##### Diagnosis.

With the attributes of the genus *Asklepia* as described by Liebke (1938) and as noted above under the generic diagnosis, and small in size for the genus (SBL = 2.494 mm). Adults with head fuscous, prothorax brunneus, elytral maculae aurantiacus; elytron brunneus with a broad rectangular-shaped aurantiacus macula crossing basal and medial lateral and proximal quadrants, macula not extended to lateral margin or sutural area and, a rounded flavous macula in the apical proximal quadrant, sutural area fulvous; metasternum, abdominal sterna III-VI, and epipleuron testaceous, abdominal sternum VII paler; legs testaceous; antennal scape, pedicel, and antennomere 3 testaceous, antennomeres 4-7 deeply infuscated, 8-11 white. Dorsal surface devoid of microsculpture, surface luster very shiny. Pronotum cordiform, narrowly explanate; anterior angles feebly produced; lateral margin beaded; hind angle angulate, moderately prominent; median line well defined; basal and apical transverse impression punctate, punctures infuscated. Elytral interneurs evident as rows of continuous coarse punctures closely spaced.

##### Description.

(Fig. 35, 61). ***Habitus*:** (Fig. 35). ***Size*:** [See also Table 7] Small-size for the genus; ABL = 2.99 mm, SBL = 2.494 mm, TW (total width) 1.463 mm, LP = 0.489 mm, WP = 0.706 mm, LE = 1.633 mm. ***Color*:** See diagnosis above. ***Luster*:** See diagnosis above. ***Head*** (Fig. 35): as in description for genus above. ***Prothorax*.** Pronotum (Fig. 35) moderately broad, as wide as head across eyes (WH/WP: 1.032), longer than head (LP/LH: 1.316), wider than long (W/L: 1.444); markedly cordiform and explanate, lateral margin beaded with seta at anterior third; apical margin straight; base markedly constricted; hind angle moderately produced and setose; median line well defined, basal and apical transverse impression punctate, punctures infuscated; surface smooth throughout. ***Pterothorax*.** Normal for genus, see description for genus above. Elytra slightly convex; at apical third markedly wider than head across eyes (WH/TW: 0.468) and twice as wide as pronotum (WP/TW: 0.504). Elytral interneurs evident as rows of continuous, coarse punctures widely spaced, coarse punctures with infuscated halo in the proximal basal and apical quadrants. Hind wings fully developed. ***Legs*.** Overall, normal for genus, see description for genus above. ***Abdominal sterna*.** Overall, normal for genus, see description for genus above. ***Male genitalia*** (Fig. 61). Median lobe (**ml**) with phallobase (**pb**) of moderate length about a fourth the length of shaft (**ps**), basal opening (**bo**) large, oriented parallel to shaft’s apical third. Shaft broad, moderately curved ventrally, dorsally sclerotized except for short ostium (**oo**, **om**); in ventral aspect tapered toward rather rounded apex (**a**), in lateral aspect, a narrowly rounded apex. Left paramere (**lp**) very large and broad, right (**rp**) small and triangular, apex of left paramere lobate and much longer than right paramere, about half the length of shaft (measured in left lateral aspect). Endophallus with 5 medial spines (**ms**), and one very large distal spine (**ds**). ***Female genitalia*.** Not investigated, presumably similar to that of *Asklepia demiti* **sp. n.**

##### Dispersal potential.

These beetles are macropterous and probably capable of flight. They are moderately swift and agile runners.

##### Distribution.

(Fig. 77). This species has been found at only one location on the white-water system of the Amazon River drainage system. But that does not at all indicate its actual distribution, as has been pointed out above, very small beetles are inadequately sampled, especially in the Neotropics.

##### Way of life.

See Erwin (1991) for a general description of the genus. No way of life information is available for this species other than they occur in lowland Amazonian rainforest.

##### Other specimens examined.

None.

#### Asklepia lebioides

Taxon classificationAnimaliaColeopteraCarabidae

(Bates, 1871) comb. n.

Lebia-like pattern-wing beetle Figs 36 , 62 , 77

Eucaerus lebioides Bates, 1871:79.

##### Lectotype.

**Brazil**, Pará, Santarém, Río Tapajos, 2.4079°S, 54.7969°W, 30m, (H.W. Bates)(MNHP: ADP132553, female). This specimen was labeled “Lectotype” by George E. Ball in 1972.

##### Derivation of specific epithet.

The specific epithet, *lebioides*, is a Latin adjective that adequately describes this species with adults resembling (-oides) some adults in the lebiine genus, *Lebia*.

##### Proposed english vernacular name.

Lebia-like pattern-wing beetle.

##### Diagnosis.

With the attributes of the genus *Asklepia* as described by Liebke (1938) and as noted above under the generic diagnosis, and medium to large-sized for the genus (SBL = 2.692–3.142). Adults with head and prothorax fuscous, elytral maculae aurantiacus; elytron fuscous with a slender aurantiacus macula crossing apical lateral and proximal quadrants, macula does not reach the lateral margin and the sutural area, and a rounded aurantiacus macula in the proximal apical quadrant, macula reaches the sutural area; metasternum, abdominal sterna III-VI, and epipleuron brunneus, abdominal sternum VII paler; legs testaceous; antennal scape and pedicel testaceous, antennomeres 3–7 deeply infuscated, 8–11 white. Dorsal surface devoid of microsculpture, surface luster very shiny. Pronotum cordiform, narrowly explanate; apical margin concave, lateral margin explanate; anterior angles markedly produced, hind angle markedly produced; median line moderately defined. Elytral interneurs evident as continuous rows of coarse punctures; punctures of the apical margin each with a halo.

##### Description.

(Fig. 36, 62). ***Habitus*:** (Fig. 36). ***Size*:** [See also Table 8] Medium to large-size for the genus; ABL = 2.605–3.064 mm, SBL = 2.692–3.142 mm, TW (total width) 1.504–1.790 mm, LP = 0.551–0.647 mm, WP = 0.747–0.869 mm, LE = 1.698–2.023 mm. ***Color*:** See diagnosis above. ***Luster*:** See diagnosis above. ***Head*** (Fig. 36): as in description for genus above. ***Prothorax*.** Pronotum (Fig. 36) moderately broad, as wide as head across eyes (WH/WP, mean both sexes: 0.973) longer than head (LP/LH, mean both sexes: 1.349), wider than long (W/L: mean both sexes: 1.824); markedly cordiform and explanate, lateral margin beaded with seta at anterior third; base markedly constricted and lobed; anterior angles moderately produced, hind angle markedly acutely produced and setose; median line moderately defined, basal and apical transverse impressions punctate, punctures infuscated; surface smooth throughout. ***Pterothorax*.** Normal for genus, see description for genus above. Elytra slightly convex; at apical third twice as wide as the head across eyes (WH/TW: mean, 0.487) and pronotum (WP/TW: mean, 0.5). Elytral interneurs evident as rows of continuous fine closely spaced coarse punctures; punctures of the apical margin each with a halo. Hind wings fully developed. ***Legs*.** Overall, normal for genus, see description for genus above. ***Abdominal sterna*.** Overall, normal for genus, see description for genus above. ***Male genitalia*** (Fig. 62, see Fig. 61 for attribute labels). Median lobe with phallobase short about a fifth the length of shaft, basal opening small, oriented parallel to shaft’s apical third. Shaft broad, moderately curved ventrally, dorsally sclerotized except for short ostium; in ventral aspect tapered toward rather broadly rounded apex, in lateral aspect, a narrowly rounded apex. Left paramere very large and broad, right small and triangular, apex of left paramere lobate much longer than right paramere, about half the length of shaft (measured in left lateral aspect). Endophallus with 11 medial spines, and one large distal spine. ***Female genitalia*.** Not investigated, presumably similar to that of *Asklepia demiti* sp. n.

##### Dispersal potential.

These beetles are macropterous and probably capable of flight. They are moderately swift and agile runners.

##### Distribution.

(Fig. 77). This species has been found at locations on both the clear-water and white-water systems of the Amazon River drainage system. But that does not at all indicate its real distribution: as has been pointed out above, very small beetles are inadequately sampled, especially in the Neotropics.

##### Way of life.

See Erwin (1991) for a general description of the genus. Adults of this species are active in lowland Varzea rainforest in the late rainy season.

##### Other specimens examined.

**Brazil**, Brazil, Pará, Santarém, Río Tapajos, 2.4079°S, 54.7969°W, 30m, (H.W. Bates)(BMNH: ADP109194, male paralectotype); Amazonas, circa Rio Demiti, 0.5748°N, 66.6869°W, 116m, 22 August 1978 (G.E. Ball, K.E. Ball)(UASM: ADP109208, ADP130048, ADP130052, ADP130050, males), 13 September 1978 (G.E. Ball, K.E. Ball)(NMNH: ADP109202, ADP132689, ADP133133, ADP132506, ADP132731, females, ADP132687, ADP133181, ADP133145, ADP132751, ADP133159, ADP132691, ADP132719, ADP132729, ADP132733, ADP133131, ADP133026, ADP132499, ADP133103, circa Cucui, Rio Negro, 1.1972°N, 66.8382°W, 79m, 17 September 1978 (G.E Ball, K.E. Ball)(NMNH: ADP132701, male).

##### Note.

Bates (1871) mentions having three specimens. We have studied the lectotype from Paris (MNHP) and a paralectotype from London (BMNH), but we did not see Bates’ third specimen and its location is unknown.

#### Asklepia matomena

Taxon classificationAnimaliaColeopteraCarabidae

Zamorano & Erwin sp. n.

http://zoobank.org/A75BFD33-D209-4B7A-BE42-DC480915524B

**Figure 37–40.**
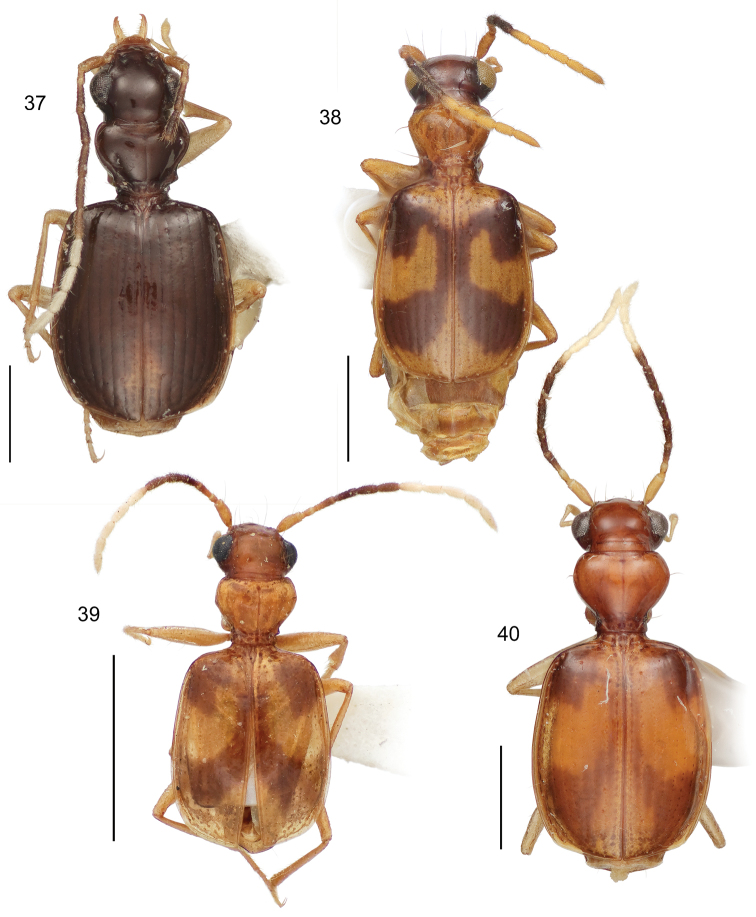
Digital Photo-illustrations, habitus, dorsal aspect of holotypes. **37** *Asklepia matomena* Zamorano & Erwin, sp. n. ADP132519, 20 km SW Manaus, Brazil **38** *Asklepia adisi* Erwin & Zamorano, sp. n. Adis #000691, Ilha de Marchantaria, Lago Camaleão, Brazil **39** *Asklepia asuncionensis* Erwin & Zamorano, sp. n. ADP130036, Asunción, Rio Paraguay, Paraguay **40** *Asklepia biolat* Erwin & Zamorano, sp. n. ADP132509, Pakitza, Perú. Scale line = 1 mm.

Reddish pattern-wing beetle Figs 37 , 63 , 77

##### Holotype.

**Brazil**, Amazonas, 20 km SW Manaus, 3.166°S, 60.234°W, 47m, 6 November 1969 (J.M. Campbell, B.A. Campbell)(NMNH: ADP132519, male).

##### Derivation of specific epithet.

The specific epithet, *matomena*, is derived from the Greek ματωμενα (bleeding) and used as an adjective in reference to the blood-red color of the head and elytra of these beetles.

##### Proposed english vernacular name.

Reddish pattern-wing beetle.

##### Diagnosis.

With the attributes of the genus *Asklepia* as described by Liebke (1938) and as noted above under the generic diagnosis, and small to medium-sized for the genus (SBL = 2.189–2.551 mm). Adults with head and prothorax fuscous, elytral maculae if present aurantiacus; elytron fuscous with a small semicircular aurantiacus macula in the proximal apical quadrant, macula does not reach the apical margin; metasternum, abdominal sterna III-VI, and epipleuron brunneus, abdominal sternum VII paler; legs testaceous; antennal scape and pedicel testaceous, antennomeres 3-7 deeply infuscated, 8-11 white. Dorsal surface devoid of microsculpture, surface luster very shiny. Pronotum cordiform, narrowly explanate, with medial lobe at base, lateral margin beaded; anterior angles feebly produced, hind angle angulated, very prominent; median line moderately defined. Elytral interneurs evident as continuous rows of fine closely spaced punctures.

##### Description.

(Figs 37, 63). ***Habitus*:** (Fig. 37). ***Size*:** [See also Table 9] Small to medium-sized for the genus; ABL = 2.476–2.961 mm, SBL = 2.189–2.551 mm, TW (total width) 0.575–0.726 mm, LP = 0.447–0.546 mm, WP = 0.603–0.730 mm, LE = 1.411–1.622 mm. ***Color*:** See diagnosis above. ***Luster*:** See diagnosis above. ***Head*** (Fig. 37): as in description for genus above. ***Prothorax*.** Pronotum (Fig. 37) moderately broad, as wide as head across eyes (WH/WP, mean males: 1.011), longer than head (LP/LH, mean males: 1.387), wider than long (W/L, mean males 1.345); markedly cordiform and explanate, lateral margin beaded with seta at anterior third; base markedly constricted and lobed medially; anterior angles feebly produced, hind angle markedly prominent, produced and setose; median line moderately defined, apical transverse impression punctate, punctures infuscated; surface smooth throughout. ***Pterothorax*.** Normal for genus, see description for genus above. Elytra slightly convex; at apical third twice as wide as head across eyes (WH/TW: mean males, 0.520) and pronotum (WP/TW: mean males, 0.514), longer than wide. Elytral interneurs evident as rows of continuous fine punctures closely spaced; punctures homogeneous. Hind wings fully developed. ***Legs*.** Overall, normal for genus, see description for genus above. ***Abdominal sterna*.** Overall, normal for genus, see description for genus above. ***Male genitalia*** (Fig. 63, see Fig. 61 for attribute labels). Median lobe with phallobase short about a fourth the length of shaft, basal opening small, oriented parallel to shaft. Shaft broad, slightly curved ventrally, dorsally sclerotized except for short ostium; in ventral aspect tapered toward rather narrowly acute apex, in lateral aspect, a rounded apex. Parameres: left very large and broad, right small and triangular; apex of left paramere lobate much longer than right paramere about half the length of shaft (measured in left lateral aspect). Endophallus with 7 preapical spines. ***Female genitalia*.** Not investigated, presumably similar to that of *Asklepia demiti* sp. n.

##### Dispersal potential.

These beetles are macropterous and probably capable of flight. They are moderately swift and agile runners.

##### Distribution.

(Fig. 77). This species has been found at only one location on the shore of a small lake near the middle Amazon River drainage system. But that does not at all indicate its real distribution: as has been pointed out above, very small beetles are inadequately sampled, especially in the Neotropics.

##### Way of life.

See Erwin (1991) for a general description. Adults of this species are active in lowland rainforest in the transition from rainy to dry seasons.

##### Other specimens examined.

**Brazil**, Amazonas, 20 km SW Manaus, 3.166°S, 60.234°W, 47m, 6 November 1969 (J.M. Campbell, B.A. Campbell)(NMNH: ADP133527, ADP133097, male paratypes).

### *pulchripennis* species group

Dorsal color variable, some species with lighter adults and some with darker ones; abdominal sterna III-VI aurantiacus, sternum VII infuscated. Without explanate pronotal lateral margins, although some with a partial bead from the hind angle to apical third and some with a bead extended slightly beyond the lateral seta; pronotum sub-spherical in anterior two-thirds; elytra patterned. Brachyptery present in two species. Endophallus of aedeagus with two rather large spines, one larger than the other.

#### Asklepia adisi

Taxon classificationAnimaliaColeopteraCarabidae

Erwin & Zamorano sp. n.

http://zoobank.org/01A360AD-0E9E-4626-B2FC-D90DB089F19F

Adis’ pattern-wing beetle Figs 38 , 64 , 78

##### Holotype.

**Brazil**, Amazonas, Rio Solimões, Ilha de Marchantaria, Lago Camaleão, 3.2488°S, 59.9556°W, 7m, 11 April 1981 (NMNH: ADIS # 000691, female).

##### Derivation of specific epithet.

The specific epithet, *adisi*, is an eponym, masculine, genitive case, based on the family name of Joachim Adis**^†^** who collected the type series.

##### Proposed english vernacular name.

Adis’ pattern-wing beetle.

##### Diagnosis.

With the attributes of the genus *Asklepia* as described by Liebke (1938) and as noted above under the generic diagnosis, and medium-sized for the genus (SBL = 2.542–2.688 mm). Adults with head brunneus, prothorax aurantiacus, elytral maculae aurantiacus; elytron fuscous with a broad triangular aurantiacus macula entirely covering proximal basal quadrant and humeral area of lateral basal quadrant, medial quadrants largely aurantiacus, sutural area fuscous, lateral margin, apical margin and sutural area aurantiacus; metasternum fulvous, abdominal sterna with III–VI, and epipleuron fulvous, abdominal sternum VII fuscous; legs flavotestaceous; antennal scape, pedicel, antennomere 3 and basal half of 4 testaceous, apical half of antennomere 4, 5–6 deeply infuscated, 7–11 white. Dorsal surface devoid of microsculpture, surface luster very shiny. Pronotum markedly convex with lateral margin effaced except just anterior to hind angle and there a simple bead; hind angle moderately prominent; median line feebly defined. Elytral interneurs evident as short discontinuous rows of widely spaced coarse punctures.

##### Description.

(Fig. 38, 64). ***Habitus*:** (Fig. 38) ***Size*:** [See also Table 10] Medium-size for the genus; ABL = 2.582–3.199 mm, SBL = 2.542–2.688 mm, TW (total width) 1.341–1.473 mm, LP = 0.513–0.591 mm, WP = 0.660–0.717 mm, LE = 1.578–1.712 mm. ***Color*:** See diagnosis above. ***Luster*:** See diagnosis above. ***Head*** (Fig. 38): as in description for genus above. ***Prothorax*.** Pronotum (Fig. 38) moderately broad, slightly narrower than head across eyes (WH/WP, mean both sexes: 1.103), longer than head (LP/LH, mean both sexes: 1.327), wider than longer (WP/LP, mean both sexes: 1.226); markedly cordiform and convex, lateral margin effaced with seta at anterior third on slightly raised area; apex markedly constricted; anterior angle feebly produced, hind angle slightly produced and setose; median line feebly define as an infuscate line, transverse impressions punctate, punctures infuscate; surface smooth throughout. ***Pterothorax*.** Normal for genus, see description for genus above. Elytra markedly convex; at apical third twice as wide as head across eyes (WH/TW, mean both sexes: 0.535) and pronotum (WP/TW, mean both sexes: 0.520), longer than wide. Elytral interneurs evident as short discontinuous rows of widely spaced coarse punctures. Hind wings fully developed. ***Legs*.** Overall, normal for genus, see description for genus above. ***Abdominal sterna*.** Overall, normal for genus, see description for genus above. ***Male genitalia*** (Fig. 64, see Fig. 61 for attribute labels). Median lobe with phallobase moderate in length, about a fourth the length of shaft, basal opening large, oriented parallel to the central part of the shaft. Shaft broad, slightly twisted ventrally, dorsally sclerotized except for short ostium; in ventral aspect tapered toward rather broad apex, in lateral aspect, a thick rounded apex. Left paramere very large and broad, right small and triangular; apex of left paramere lobate much longer than right paramere, about two-thirds the length of shaft (measured in left lateral aspect). Endophallus with one median spine, and one large distal spine. ***Female genitalia*.** Not investigated, presumably similar to that of *Asklepia demiti* sp. n.

##### Dispersal potential.

These beetles are macropterous and capable of flight as they have been captured at lights. They are moderately swift and agile runners.

##### Distribution.

(Fig. 78). This species has been found at locations on both the clear-water and black-water systems of the upper and middle Amazon River drainage system. But that does not at all indicate its real distribution: as has been pointed out above, very small beetles are inadequately sampled, especially in the Neotropics.

##### Way of life.

See Erwin (1991) for a general description. Adults of this species are active in the rainy season in the Varzea rainforest along the main course of the Rio Solimões. They occur at the Varzea forest edge on the floating macrophyte, *Eichornia crassipes* (Mart.) Solms and on culms of the creeping river grass, *Echinochloa polystachya* (Kunth) Hitchc. In addition, they are climbers and have been found in Varzea forest, some 3.6 m above ground, on *Macrolobium acaciifolium* (Benth.) Benth., a small tree in the Fabaceae. In Perú, one individual was found on a black water river (Igapó) and was attracted to lights on our boat.

##### Other specimens examined.

**Brazil**, Amazonas, Rio Solimões, Ilha de Marchantaria, Lago Camaleão, 3.2488°S, 59.9556°W, 7m, 11 April 1981 (NMNH: ADIS # 001149, female paratype), 28 July 1981 (NMNH: ADIS # 001650, female); 14 September 1981 (NMNH: ADIS # 000356, female paratype); 1 October 1981 (J. Adis)(NMNH: ADIS # 000662, ADIS # 000621, ADIS # 000667, ADIS # 000664, ADIS # 000666, ADIS # 000233, ADIS # 000239, ADIS # 000236, ADIS # 000241, ADIS # 000231, female paratypes, ADIS # 000670, ADIS # 000622, ADIS # 000659, ADIS # 000226, ADIS # 000232, ADIS # 000238, ADIS # 000240, ADIS # 000228, ADIS # 000265, ADIS # 000653, male paratypes), 1 October 1981 (NMNH: Adis # 000656, male, not paratype, damaged, Adis # 000185, female, not paratype, damaged), 20 October 1981 (NMNH: ADIS # 000750, ADIS # 000727, female paratypes, ADIS # 001335, ADIS # 001106, ADIS # 001623, ADIS # 001344, ADIS # 001418, male paratypes) 4 November 1981 (NMNH: ADIS # 001106, females); 13–17 September 1991 (C. Martius, A. Rebello)(NMNH: ADIS # 000849, male paratype); Pará, Santarém, Río Tapajos, 2.4079°S, 54.7969°W, 30m, 27-28 December 1967 (H. Reichardt)(MZUSP: ADP132811, ADP132817, ADP132809, male paratypes, ADP132807, ADP132805, ADP133655, ADP132819, ADP132813, female paratypes, Pacoval, Rio Curuá, 1.7733°S, 54.9971°W, 12m, 16 February 1968 (H. Reichardt)(MZUSP: ADP132815, female paratype). **Perú**, Loreto, Boca del Río Samiria, 1 km SW Vigilante post No. 1, 4.5005°S, 74.0659°W, 99m, 6 May 1990 (T.L. Erwin)(MUSM: ADP093365, female).

#### Asklepia asuncionensis

Taxon classificationAnimaliaColeopteraCarabidae

Erwin & Zamorano sp. n.

http://zoobank.org/2410C225-543E-46ED-B94A-FAD157AF8157

Asunción pattern-wing beetle Figs 39 , 78

##### Holotype.

**Paraguay**, Central, Asunción, Rio Paraguay, 25.320°S, 57.668°W, 54m, June (unknown)(CMNH: ADP130036, female).

##### Derivation of specific epithet.

The specific epithet, *asuncionensis*, is a singular Latinized feminine noun in apposition, based on the name of the place near where these beetles are found.

##### Proposed english vernacular name.

Asunción pattern-wing beetle.

##### Diagnosis.

With the attributes of the genus *Asklepia* as described by Liebke (1938) and as noted above under the generic diagnosis, and small-size for the genus (SBL = 2.477 mm). Adults with head aurantiacus, prothorax fulvous, elytral maculae fulvous or aurantiacus; elytron brownish with a broad triangular flavous macula covering most of proximal apical quadrant and lower half of lateral apical quadrant, broad flavous macula ending in hook crossing from medial lateral quadrant to right half of medial proximal quadrant and almost reaching the proximal margin, small rectangular flavous macula in the upper right corner of basal proximal quadrant, apical, basal, and lateral margin broadly fulvous; metasternum fulvous, abdominal sterna with III-VI, and epipleuron fulvous, abdominal sternum VII fuscous; legs flavotestaceous; antennal scape, pedicel and antennomere 3 testaceous, antennomeres 4-6 infuscated, 7-11 white. Dorsal surface devoid of microsculpture, surface luster very shiny. Pronotum markedly convex with lateral margin effaced except just anterior to hind angle and there a simple bead; hind angle moderately prominent; anterior angles feebly produced; median line moderately defined. Elytral interneurs evident as continuous rows of subsurface dots in the substantially transparent elytron.

##### Description.

***Habitus*** (Fig. 39). ***Size*:** [See also Table 11] Small-size for the genus; ABL = 2.70 mm, SBL = 2,477 mm, TW (total width) 1.270 mm, LP = 0.533 mm, WP = 0.648 mm, LE = 1.566 mm. ***Color*:** See diagnosis above. ***Luster*:** See diagnosis above. ***Head*** (Fig. 39): as in description for genus above. ***Prothorax*.** Pronotum (Fig. 39) moderately broad, as wide as head across eyes (WH/WP,1.035 mm), longer than head (LP/LH, mean both sexes: 1.410), wider than long (W/L, mean both sexes: 1.714); markedly cordiform and rounded, lateral margin effaced with seta at anterior third on slightly raised area; apex markedly constricted; anterior angle feebly produced, hind angle slightly produced and setose; median line moderately defined, apical transverse impressions punctate, punctures infuscated; surface smooth throughout. ***Pterothorax*.** Normal for genus, see description for genus above. Elytra moderate convex; at apical third twice as wide as head across eyes (WH/TW, mean both sexes: 1.071) and pronotum (WP/TW, mean both sexes: 1.018). Elytral interneurs evident as short discontinuous rows of widely spaced coarse punctures, interneurs effaced in the medial quadrants. Hind wings fully developed. ***Legs*.** Overall, normal for genus, see description for genus above. Male unknown. ***Female genitalia*.** Not investigated, presumably similar to that of *Asklepia demiti* sp. n.

##### Dispersal potential.

These beetles are macropterous and probably capable of flight. They are moderately swift and agile runners.

##### Distribution.

(Fig. 78). This species has been found at only one location on white-water of the middle Río Paraguay drainage system. But that does not at all indicate its real distribution: as has been pointed out above, very small beetles are inadequately sampled, especially in the Neotropics.

##### Way of life.

See Erwin (1991) for a general description.

##### Other specimens examined.

None.

#### Asklepia biolat

Taxon classificationAnimaliaColeopteraCarabidae

Erwin & Zamorano sp. n.

http://zoobank.org/F1BBAF55-DA55-407E-A3FD-12C00891707A

Biolat pattern-wing beetle Figs 40 , 65 , 78

##### Holotype.

**Perú**, Madre de Dios, BIOLAT Biological Station, Pakitza, Zone 9, 11.8931°S, 71.2564°W, 382m, 1 October 1989 (T.L. Erwin)(MUSM: ADP132509, male).

##### Derivation of specific epithet.

The specific epithet, *biolat*, is used as a noun in apposition based on the acronym of the Smithsonian Institution’s past program “Biodiversity in Latin America” (BIOLAT) which sought to field-train young Latin American biology students in biodiversity techniques and did so for more than 200 of them between 1987 and 1991 in Perú and Bolivia. These beetles were collected under the auspices of the BIOLAT Program.

##### Proposed english vernacular name.

Biolat pattern-wing beetles.

##### Diagnosis.

With the attributes of the genus *Asklepia* as described by Liebke (1938) and as noted above under the generic diagnosis, and medium to large-sized for the genus (SBL = 2.787–3.247 mm). Adults with head, prothorax, and elytral maculae (slightly fulvous in some individuals); elytron fuscous with scutellar area, basal third of disc, and apical sutural area aurantiacus; metasternum, abdominal sterna III-VI, and epipleuron flavotestaceous, abdominal sternum VII infuscated; legs testaceous; antennal scape and pedicel testaceous, antennomeres 3-6 and base of 7 deeply infuscated, apical half of 7 and 8–11 white. Dorsal surface devoid of microsculpture, surface luster very shiny. Pronotum markedly convex with lateral margin effaced except just anterior to hind angle and there a simple bead; hind angle acute, not very prominent. Elytral interneurs evident as rows of subsurface dots in the substantially transparent elytron.

##### Description.

(Fig. 40, 65). ***Habitus*:** (Fig. 40). ***Size*:** [See also Table 12] Large-size for the genus; ABL = 3.127–3.421 mm, SBL = 2.787–3.247 mm, TW (total width) 0.694–0.908 mm, LP = 0.637–0.746 mm, WP = 0.747–0.935 mm, LE = 1.750–2.035 mm. ***Color*:** See diagnosis above. ***Luster*:** See diagnosis above. ***Head*** (Fig. 40): as in description for genus above. ***Prothorax*.** Pronotum (Fig. 40) moderately broad, not quite as wide as head across eyes (WH/WP, mean both sexes: 1.007), longer than head (LP/LH, mean both sexes: 1.603), slightly wider than long (WP/LP, mean both sexes: 1.244); markedly cordiform, lateral margin effaced with seta at anterior third on slightly raised area; base markedly constricted; hind angle slightly acutely produced and setose; surface markedly smooth throughout. ***Pterothorax*.** Normal for genus, see description for genus above. Elytron twice as wide as head across eyes (WH/TW: mean, both sexes, 0.522), and pronotum (WP/TW, mean both sexes: 0.518), longer than wide, moderately convex, intervals and interneurs effaced (see diagnosis above), interneur 2 with 7 setae, interneur 5 with two setae. Hind wings dimorphic; most specimens studied are brachypterous. ***Legs*.** Overall, normal for genus, see description for genus above. ***Abdominal sterna*.** Overall, normal for genus, see description for genus above. ***Male genitalia*** (Fig. 65, see Fig. 61 for attribute labels). Median lobe with phallobase moderate in length, about a fifth the length of shaft, basal opening large, oblique to shaft. Shaft broad, slightly curved ventrally at distal sixth, dorsally sclerotized except for short ostium; in ventral aspect tapered toward rather moderately rounded apex, in lateral aspect, a rounded blunt apex. Left paramere very large and broad, right small and triangular; apex of left paramere lobate much longer than right paramere about half the length of shaft (measured in left lateral aspect). Endophallus with one very large preapical spine. ***Female genitalia*.** Not investigated, presumably similar to that of *Asklepia demiti* sp. n.

##### Dispersal potential.

These beetles are macropterous and probably capable of flight. They are moderately swift and agile runners.

##### Distribution.

(Fig. 78). This species has been found at locations on the white-water system of the upper Amazon River drainage system. But that does not at all indicate its real distribution: as has been pointed out above, very small beetles are inadequately sampled, especially in the Neotropics.

##### Way of life.

See Erwin (1991) for a general description of the genus. Adults of this species are active in all seasons in lowland rainforest along small stony stream margins in wet leaf litter on sand and are active at night. They also inhabit wet leaf litter lying on half submerged tree trunks and branches. In addition, they climb culms of *Paspalum* grasses at river edges.

##### Other specimens examined.

**Perú**, Madre de Dios, BIOLAT Biological Station, Pakitza, Zone 9, 11.8931°S, 71.2564°W, 382m, 1 October 1989 (T.L. Erwin)(NMNH: ADP132525, ADP132524, ADP132490, ADP132505, ADP132492, ADP132508, female paratypes, ADP132509, ADP132467, ADP132474, ADP132481, ADP132487, ADP132489, ADP132484, ADP132469, ADP132475, ADP132463, ADP132478, male paratypes), Río Manu, 11.9350°S, 71.3032°W, 329m, 18 & 21 February 1990 (T.L. Erwin, E. Pfuno S., F. Pfuno S.)(NMNH: ADP132575, ADP132480, male paratypes); Río Manu, Pakitza, 11.5647°S, 71.1700°W, 356m, 11 July 1992 (T.L. Erwin, E. Pfuno S., F. Pfuno S.)(NMNH: ADP109198, ADP132996, female paratypes); 11.9350°S, 71.3032°W, 329m, 13 July 1992 (T.L. Erwin, E. Pfuno S., F. Pfuno S.)(NMNH: ADP132988, female paratype, ADP133195, male paratype); 11.9099°S, 71.2717°W, 395m, 24 June 1993 (T.L. Erwin, F. Pfuno S.)(NMNH: ADP133189, female paratype, ADP133193, ADP133149, ADP132707, ADP133171, male paratypes).

##### Notes.

See Erwin (1990) and Venable & Erwin (1996) for detailed trail maps of the BIOLAT Biodiversity Station.

#### Asklepia bracheia

Taxon classificationAnimaliaColeopteraCarabidae

Zamorano & Erwin sp. n.

http://zoobank.org/05701FC7-7994-4D81-A068-849C5F60FD85

**Figure 41–44.**
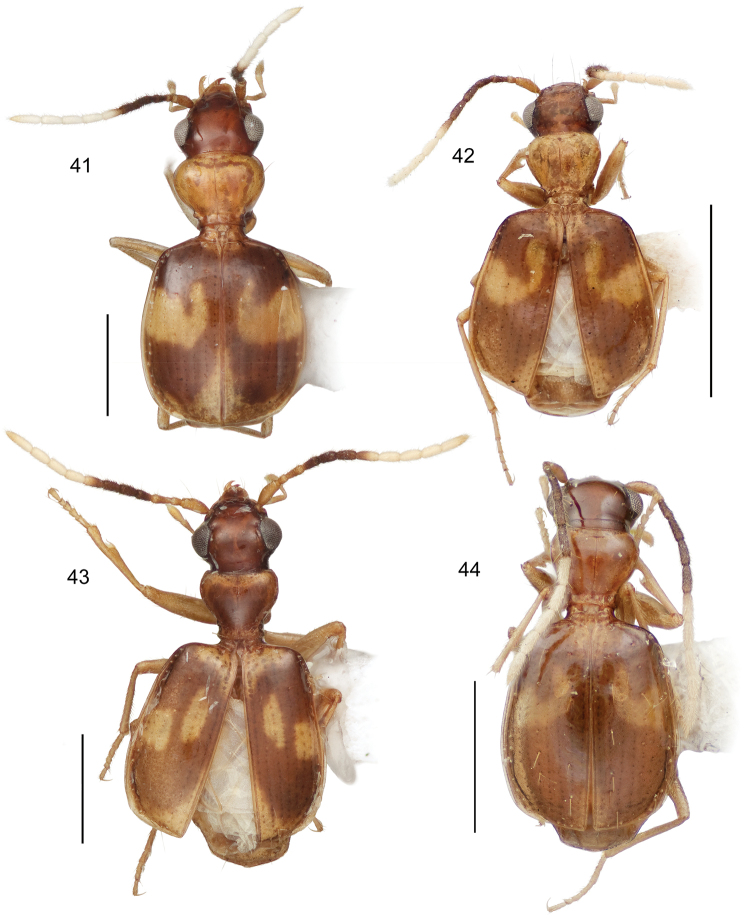
Digital Photo-illustrations, habitus, dorsal aspect of holotypes. **41** *Asklepia bracheia* Zamorano & Erwin, sp. n. ADP008121, Cocha Shimagai, Perú **42** *Asklepia cuiabaensis* Erwin & Zamorano, sp. n. ADP130044, Cuiabá, Brazil **43** *Asklepia ecuadoriana* Erwin & Zamorano, sp. n. ADP132485, Limoncocha, Ecuador **44** *Asklepia kathleenae* Erwin & Zamorano, sp. n. ADP132460, Belém, Brazil. Scale line = 1 mm.

Short pattern-wing beetle Fig. 41 , 66 , 78

##### Holotype.

**Perú**, Loreto, circa Explornapo Camp, Río Napo, Cocha Shimagai, 3.3563°S, 73.0467°W, 88m, 13 June 1992 (T.L. Erwin, E. Pfuno S., F. Pfuno S.)(NMNH: ADP08121, female).

##### Derivation of specific epithet.

The specific epithet, *bracheia*, is derived from the Greek βραχεια (small) and used as an adjective in reference to the small size of the elytra of these beetles.

##### Proposed english vernacular name.

Short pattern-wing beetles.

##### Diagnosis.

With the attributes of the genus *Asklepia* as described by Liebke (1938) and as noted above under the generic diagnosis, and small-sized for the genus (SBL = 1.952–2.345 mm). Adults with head aurantiacus, prothorax and elytral maculae fulvous or aurantiacus in some individuals; elytron fuscous with basal proximal quadrant, medial lateral quadrant and apical proximal quadrant fulvous, lateral margin in all the quadrants fulvous; metasternum, abdominal sterna III-VI, and epipleuron fulvous, abdominal sternum VII fuscous; legs fulvous; antennal scape, pedicel and apical half of antennomere 3 testaceous, antennomeres 4-7 and basal half of 3 deeply infuscated, apical half of 7 and 8-11 white. Dorsal surface devoid of microsculpture, surface luster very shiny. Pronotum markedly convex with lateral margin effaced except just anterior to hind angle and there a simple bead; hind angle not very prominent. Elytral interneurs evident as short discontinuous rows of widely spaced punctures, rows effaced at medial quadrants of elytron, punctures fuscous; elytron substantially transparent.

##### Description.

(Figs 41, 66). ***Habitus*:** (Fig. 41). ***Size*:** [See also Table 13] Medium-size for the genus; ABL = 2.343–2.537 mm, SBL = 1.952–2.344 mm, TW (total width) 1.074–1.274 mm, LP = 0.457–0.558 mm, WP = 0.557–0.701 mm, LE = 1.275–1.424 mm. ***Color*:** See diagnosis above. ***Luster*:** See diagnosis above. ***Head*** (Fig. 41): as in description for genus above. ***Prothorax*.** Pronotum (Fig. 41) slightly broad, about as wide as head across eyes (WH/WP, mean both sexes: 1.042), longer than head (LP/LH, mean both sexes: 1.590), wider than long (WP/LP: mean both sexes: 1.205); markedly cordiform, lateral margin effaced with seta at anterior third on slightly raised area; base markedly constricted; anterior angles feebly produced, hind angle slightly produced and setose; median line feebly defined, basal and apical transverse impressions evident as infuscated dots in the surface, surface smooth throughout. ***Pterothorax*.** Normal for genus, see description for genus above. Elytra moderately convex; at apical third twice as wide as head across eyes (WH/TW, mean both sexes: 0.529) and pronotum (WP/TW, mean both sexes: 0.507), longer than wide. Elytral interneurs evident as discontinuous rows of widely spaced punctures, rows effaced at lateral area of medial third and apical third, punctures present fuscous; elytron substantially transparent. Hind wings fully developed. ***Legs*.** Overall, normal for genus, see description for genus above. ***Abdominal sterna*.** Overall, normal for genus, see description for genus above. ***Male genitalia*** (Fig. 66, see Fig. 61 for attribute labels). Median lobe with phallobase long about a third the length of shaft, basal opening large, oriented parallel to shaft at apical third. Shaft narrow, slightly curved ventrally, dorsally sclerotized except for short ostium; in ventral aspect tapered toward rather moderately narrow rounded apex, in lateral aspect, a rounded apex. Parameres: left very large and broad, right small and triangular; apex of left paramere lobate much longer than right paramere about half the length of shaft (measured in left lateral aspect). Endophallus with 2 preapical spines. ***Female genitalia*.** Not investigated, presumably similar to that of *Asklepia demiti* sp. n.

##### Dispersal potential.

These beetles are macropterous and probably capable of flight. They are moderately swift and agile runners.

##### Distribution.

(Fig. 78). This species has been found at locations on the black-water systems of the upper Amazon River drainage system. But that does not at all indicate its real distribution: as has been pointed out above, very small beetles are inadequately sampled, especially in the Neotropics.

##### Way of life.

See Erwin (1991) for a general description. Adults of this species are active in the rainy season at the edges of streams and lakes in wet leaf litter and in muddy swales left by inundation of high river levels.

##### Other specimens examined.

**Perú**, Loreto, 1 km SW Boca del Río Samiria, Vigilante post No. 1, 4.5005°S, 74.0659°W, 99m, 14 & 15 August 1991 (T.L. Erwin, G.E. Ball, D. Shpeley)(NMNH: ADP133028, female paratype, ADP133169, male paratype), 5 May 1990 (T.L. Erwin, G.E. Ball, D. Shpeley)(NMNH: ADP086696, female paratype, ADP086682, ADP086742, ADP067305, ADP067304, male paratypes); circa Explornapo Camp, Río Napo, Cocha Shimagai, 3.3563°S, 73.0467°W, 88m, 13 June 1992 (T.L. Erwin, E. Pfuno S., F. Pfuno S.)(NMNH: ADP008125, female paratype, ADP008072, ADP008120, male paratypes).

#### Asklepia cuiabaensis

Taxon classificationAnimaliaColeopteraCarabidae

Erwin & Zamorano sp. n.

http://zoobank.org/CD3F2AE4-0CF7-4F6F-846F-224A25DE01A1

Cuiaba pattern-wing beetle Figs 42 , 78

##### Holotype.

Brazil, Mato Grosso, Cuiabá, 15.6416°S, 56.0732°W, 149m, August (unknown)(CMNH: ADP130044, female).

##### Derivation of specific epithet.

The specific epithet, *cuiabaensis*, is a singular Latinized feminine noun in apposition, based on the name of the place near where these beetles are found.

##### Proposed english vernacular name.

Cuiabá pattern-wing beetle.

##### Diagnosis.

With the attributes of the genus *Asklepia* as described by Liebke (1938) and as noted above under the generic diagnosis, and small-sized for the genus (SBL = 2.191 mm). Adults with head fuscous, prothorax fulvous, elytral maculae fulvous; elytron fuscous with a small and slender triangular flavous macula in the lower right corner of the proximal apical quadrant, narrow flavous macula ending in hook crossing from medial lateral quadrant to right half of apical proximal quadrant, slender quadrangular flavous macula in the upper right corner of basal proximal quadrant, apical and lateral margins fulvous; metasternum fulvous, abdominal sterna with III-VI, and epipleuron fulvous, abdominal sternum VII fuscous; legs flavotestaceous; antennal scape and pedicel testaceous, antennomeres 3-6 and basal half of 7 deeply infuscated, apical half of 7 and 8-11 white. Dorsal surface devoid of microsculpture, surface luster very shiny. Pronotum moderately convex with lateral margin effaced except just anterior to hind angle and there a simple bead; hind angle moderately prominent; anterior angles feebly produced; median line feebly defined. Elytral interneurs evident as continuous rows of widely spaced coarse punctures, punctures fuscous.

##### Description.

***Habitus*** (Fig. 42). ***Size*:** [See also Table 14] Medium-size to large for the genus; ABL = 2.610 mm, SBL = 2.191 mm, TW (total width) 1.270 mm, LP = 0.46 mm, WP = 0.592 mm, LE = 1.372 mm. ***Color*:** See diagnosis above. ***Luster*:** See diagnosis above. ***Head*** (Fig. 42): as in description for genus above. ***Prothorax*.** Pronotum (Fig. 42) moderately broad, as wide as head across eyes (WH/WP, 1,062) longer than head (LP/LH, mean both sexes: 1.388), wider than long (W/L, mean both sexes: 1.726); slightly cordiform and rounded, lateral margin effaced with seta at anterior third on slightly raised area; apex markedly constricted; anterior angle feebly produced, hind angle slightly produced and setose; median line feebly defined, apical transverse impressions punctate, surface smooth throughout. ***Pterothorax*.** Normal for genus, see description for genus above. Elytra moderate convex; at apical third twice as wide as head across eyes (WH/TW, mean both sexes: 0,495) and pronotum (WP/TW, mean both sexes: 0,495). Elytral interneurs evident as continuous rows of widely spaced coarse punctures, interneurs continuous along length of entire elytron. Hind wings fully developed. ***Legs*.** Overall, normal for genus, see description for genus above. ***Abdominal sterna*.** Overall, normal for genus, see description for genus above. ***Male genitalia*.** Male unknown. ***Female genitalia*.** Not investigated, presumably similar to that of *Asklepia demiti* sp. n.

##### Dispersal potential.

These beetles are macropterous and probably capable of flight. They are moderately swift and agile runners.

##### Distribution.

(Fig. 78). This species has been found at only one location on the middle Rio Cuiabá. But that does not at all indicate its real distribution: as has been pointed out above, very small beetles are inadequately sampled, especially in the Neotropics.

##### Way of life.

See Erwin (1991) for a general description. Adults of this species are active in August in these tropical savannah habitats with mild dry winter season; in August the air temperatures reach maxima between 30° to lower 40°C.

##### Other specimens examined.

None.

#### Asklepia ecuadoriana

Taxon classificationAnimaliaColeopteraCarabidae

Erwin & Zamorano sp. n.

http://zoobank.org/40AFE3C2-454B-42E4-998A-BBC8014E7406

Ecuadorian pattern-wing beetle Figs 43 , 67 , 68 , 78

##### Holotype.

**Ecuador**, Sucumbíos, Limoncocha, 0.4088°S, 76.6176°W, 233m, 11 June 1977 (W.E Steiner)(NMNH: ADP132485, male).

##### Derivation of specific epithet.

The specific epithet, *ecuadoriana*, is a singular Latinized feminine noun in apposition, based on the name of the country in which these beetles are found.

##### Proposed english vernacular name.

Ecuadorian pattern-wing beetle.

##### Diagnosis.

With the attributes of the genus *Asklepia* as described by Liebke (1938) and as noted above under the generic diagnosis, and medium to large-sized for the genus (SBL = 2.590–3.131 mm). Adults with head aurantiacus and prothorax flavotestaceous, elytral maculae fulvous or aurantiacus in some individuals; elytron flavotestaceous with a triangular flavous macula in the basal proximal quadrant, macula covering half of the quadrant, and elongated, longitudinally oriented flavous macula in the medial proximal quadrant, a flavous macula in the medial lateral quadrant and a triangular flavous macula in the apical proximal quadrant; metasternum, abdominal sterna III-VI, and epipleuron flavotestaceous, abdominal sternum VII infuscated; legs testaceous; antennal scape, pedicel and antennomere 3 testaceous, antennomeres 4-6 and basal half of 7 deeply infuscated, apical half of 7, 8-11 white. Dorsal surface devoid of microsculpture, surface luster very shiny. Pronotum markedly convex with lateral margin effaced except just anterior to hind angle and there a simple bead; hind angle feebly produced; median line feebly defined. Elytral interneurs effaced from most of the elytron surface, only evident as short discontinuous rows of course punctures in the basal proximal quadrant and upper right corner of basal lateral quadrant.

##### Description.

(Figs 43, 67, 68). ***Habitus*:** (Fig. 43). ***Size*:** [See also Table 15] Medium-size for the genus; ABL = 2.894–2.995 mm, SBL = 2.501–2.598 mm, TW (total width) 1.325–1.490 mm, LP = 0.555–0.595 mm, WP = 0.638–0.691 mm, LE = 1.607–1.632 mm. ***Color*:** See diagnosis above. ***Luster*:** See diagnosis above. ***Head*** (Fig. 43): as in description for genus above. ***Prothorax*.** Pronotum (Fig. 43) moderately broad, slightly narrower than head across eyes (WH/WP, mean both sexes: 1.140), longer than head (LP/LH, mean both sexes: 1.586), about as longer than wide (WP/LP, mean both sexes: 1.157); markedly cordiform and convex lateral margin effaced with seta at anterior third on slightly raised area; apex markedly constricted; anterior angles feebly produced, hind angle slightly acutely produced and setose; median line feebly defined, basal transverse impressions punctate, punctures infuscated; surface smooth throughout. ***Pterothorax*.** Normal for genus, see description for genus above. Elytra moderately convex; at apical third twice as wide as head across eyes (WH/TW, mean both sexes: 0.535) and pronotum (WP/TW, mean both sexes: 0.469), longer than wide. Elytral interneurs effaced from most of the elytron surface, only evident as short discontinuous rows of coarse punctures, punctures with fuscous halo. Scattered fuscous punctures in the medial and apical quadrants present in some individuals. Hind wings fully developed. ***Legs*.** Overall, normal for genus, see description for genus above. ***Abdominal sterna*.** Overall, normal for genus, see description for genus above. ***Male genitalia*** (Figs 67, 68, see Fig. 61 for attribute labels). Median lobe with phallobase short about a fourth the length of shaft, basal opening small, oriented parallel to shaft. Shaft broad, slightly curved ventrally then curved dorsally near apex, dorsally sclerotized except for short ostium; in ventral aspect tapered toward rather broadly rounded apex, in lateral aspect, a narrowly rounded apex. Parameres: left very large and broad, right small and triangular; apex of left paramere narrowly rounded much longer than right paramere about half the length of shaft (measured in left lateral aspect). Endophallus with 2 preapical spines; we have illustrated an everted endophallus to demonstrate the location of the spines in a median field and an apical field. ***Female genitalia*.** Not investigated, presumably similar to that of *Asklepia demiti* sp. n.

##### Dispersal potential.

These beetles are macropterous and probably capable of flight. They are moderately swift and agile runners.

##### Distribution.

(Fig. 78). This species has been found at only one location on a lake shore near the white-water system of the Río Napo drainage system. But that does not at all indicate its real distribution: as has been pointed out above, very small beetles are inadequately sampled, especially in the Neotropics.

##### Way of life.

See Erwin (1991) for a general description of the genus. Adults of this species are active in the rainy season on the shore of a small lake in lowland rainforest.

##### Other specimens examined.

**Ecuador**, Orellana, Limoncocha, 0.4088°S, 76.6176°W, 233m, 11 June 1977 (W.E Steiner)(NMNH: ADP132500, female paratype, ADP109192, ADP132468, male paratypes).

#### Asklepia kathleenae

Taxon classificationAnimaliaColeopteraCarabidae

Erwin & Zamorano sp. n.

http://zoobank.org/5E51D7E3-F8BB-4005-98F4-3688921D1674

Kathleen’s pattern-wing beetle Figs 44 , 69 , 78

##### Holotype.

**Brazil**, Pará, Belém, 5m, 1.46°S, 48.42°W, 5-8 October 1978 (G.E Ball, K.E. Ball)(NMNH: ADP132460, male).

##### Derivation of specific epithet.

The specific epithet, *kathleenae*, is an eponym, feminine singular, genitive case, based on the given name of Kathleen E. Ball, who along with her husband, George E. Ball, collected the holotype.

##### Proposed english vernacular name.

Kathleen’s pattern-wing beetle.

##### Diagnosis.

With the attributes of the genus *Asklepia* as described by Liebke (1938) and as noted above under the generic diagnosis, and small-sized for the genus (SBL = 2.167 mm). Adults with head brunneus, prothorax testaceous, elytral maculae testaceous; elytron brunneus with a small triangular macula in the basal proximal quadrant, an arc-shaped, horizontally oriented macula crossing medial lateral and proximal quadrants, sutural area of apical quadrant testaceous; metasternum, abdominal sterna III-VI, and epipleuron flavotestaceous, abdominal sternum VII slightly infuscated; legs testaceous; antennal scape and pedicel testaceous, antennomeres 3-6 deeply infuscated, 7-11 white. Dorsal surface devoid of microsculpture, surface luster very shiny. Pronotum markedly convex with lateral margin effaced except just anterior to hind angle and there a simple bead; hind angle acute, slightly prominent; median line feebly defined. Elytral interneurs evident as continuous rows of widely spaced coarse punctures.

##### Description.

(Figs 44, 69). ***Habitus*:** (Fig. 44). ***Size*:** [See also Table 16] Medium-size for the genus; ABL = 2.428 mm, SBL = 2.167 mm, TW (total width) 1.241 mm, LP = 0.484 mm, WP = 0.569 mm, LE = 1.341 mm. ***Color*:** See diagnosis above. ***Luster*:** See diagnosis above. ***Head*** (Fig. 44): as in description for genus above. ***Prothorax*.** Pronotum (Fig. 44) slightly broad, narrower than head across eyes (WH/WP: 1.135), longer than head (LP/LH: 1.414), longer than wide (WP/LP: 1.176); surface markedly cordiform, lateral margin effaced with seta at anterior third on slightly raised area; base markedly constricted; anterior angle feebly produced, hind angle slightly produced and setose, median line feebly defined, apical transverse impression punctate, punctures infuscated; smooth throughout. ***Pterothorax*.** Normal for genus, see description for genus above. Elytra slightly convex; twice as wide as head across eyes (WH/TW: 0.520) and pronotum (WP/TW: 0.459), longer than wide. Elytral interneurs evident as continuous rows of widely spaced coarse punctures; punctures infuscated; elytron substantially transparent. Hind wings fully developed. ***Legs*.** Overall, normal for genus, see description for genus above. ***Abdominal sterna*.** Overall, normal for genus, see description for genus above. ***Male genitalia*** (Fig. 69, see Fig. 61 for attribute labels). Median lobe with phallobase moderately long about a fourth the length of shaft, basal opening moderately large, oriented parallel to shaft at apical third. Shaft moderately broad, abruptly curved ventrally, dorsally sclerotized except for short ostium; in ventral aspect tapered toward rather narrowly acute apex, in lateral aspect, a moderately broad rounded apex. Parameres: left very large and broad, right small and triangular; apex of left paramere lobate much longer than right paramere about two-thirds the length of shaft (measured in left lateral aspect). Endophallus with 2 preapical spines. ***Female genitalia*.** Not investigated, presumably similar to that of *Asklepia demiti* sp. n.

##### Dispersal potential.

These beetles are macropterous and probably capable of flight. They are moderately swift and agile runners.

##### Distribution.

(Fig. 78). This species has been found at two nearby locations in open grassy swamps along the shore of the lower Río Amazonas. But that does not at all indicate its real distribution: as has been pointed out above, very small beetles are inadequately sampled, especially in the Neotropics.

##### Way of life.

See Erwin (1991) for a general description of the genus. Adults of this species are active in the transition from wet to dry seasons and occur in open grassy marshes.

##### Other specimens examined.

**Brazil**, Pará, Belém, 5m, 1.46°S, 48.42°W, 5–8 October 1978 (G.E Ball, K.E. Ball)(NMNH: ADP132529, male paratype).

#### Asklepia macrops

Taxon classificationAnimaliaColeopteraCarabidae

Erwin & Zamorano sp. n.

http://zoobank.org/9F65FBFB-63E9-484A-A195-3F4A441D8F9C

**Figure 45–48.**
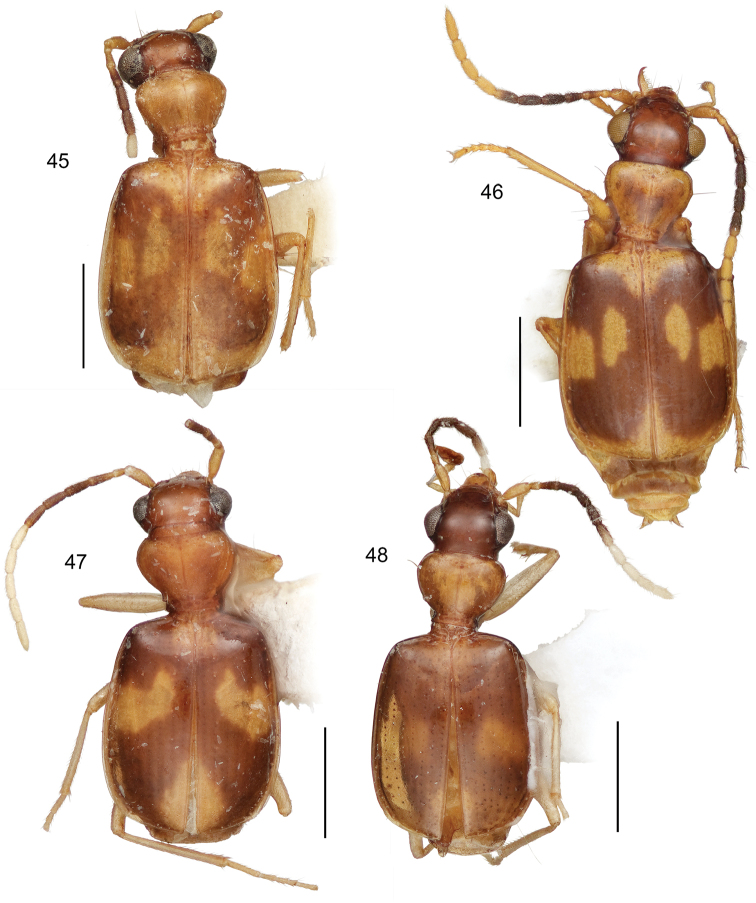
Digital Photo-illustrations, habitus, dorsal aspect of holotypes. **45** *Asklepia macrops* Erwin & Zamorano, sp. n. ADP132496, Concordia, Río Uruguay, Argentina **46** *Asklepia marchantaria* Erwin & Zamorano, sp. n. Adis #000727, Ilha de Marchantaria, Lago Camaleão, Brazil **47** *Asklepia marituba* Zamorano & Erwin, sp. n. ADP132494, Ananindeua, Marituba, Brazil **48** *Asklepia pakitza* Erwin & Zamorano, sp. n. ADP132466, Patkitza, Perú. Scale line = 1 mm.

Argentina pattern-wing beetle Figs 45 , 78

##### Holotype.

**Argentina**, Entre Ríos, Concordia, Río Uruguay, 31.368°S, 57.993°W, 9m, (M.A. Cazier)(AMNH: ADP132496, female).

##### Derivation of specific epithet.

The specific epithet, *macrops*, is a Latin adjective descriptive of the eyes of adults of this species, which are bigger than those of congeners.

##### Proposed english vernacular name.

Argentina pattern-wing beetles.

##### Diagnosis.

With the attributes of the genus *Asklepia* as described by Liebke (1938) and as noted above under the generic diagnosis, and large-sized for the genus (SBL = 3.054 mm). Adults with head brunneus, prothorax aurantiacus, elytral maculae aurantiacus; elytron fuscous with a broad triangular aurantiacus macula covering most of proximal basal quadrant and humeral area of lateral basal quadrant, a narrow elongated aurantiacus macula vertically oriented in the medial lateral and proximal quadrant, sutural area fuscous, lateral margin, apical margin and sutural area aurantiacus; metasternum fulvous, abdominal sterna with III-VI, and epipleuron fulvous, abdominal sternum VII fuscous; legs flavotestaceous; antennal scape, pedicel, antennomere 3 and basal half of 4 testaceous, apical half of antennomere 4, 5-6 deeply infuscated, 7-11 white. Similar to *Asklepia adisi* and *Asklepia marchantaria*, but with very large eyes in which the lateral convexity of eye from head margin slightly more than length of antennomere 3, whereas in the other species it is less than the length of antennomere 3. Dorsal surface devoid of microsculpture, surface luster very shiny. Pronotum markedly convex with lateral margin effaced except just anterior to hind angle and there a simple bead; hind angle moderately prominent; median line barely defined. Elytral interneurs effaced from the greater part of elytron, only evident as scattered infuscated punctures.

##### Description.

***Habitus*** (Fig. 45). ***Size*:** [See also Table 17] Medium-size to large-size for the genus; ABL = 3.141 mm, SBL = 3.054 mm, TW (total width) 1.648 mm, LP = 0.621 mm, WP = 0.779 mm, LE = 1.942 mm. ***Color*:** See diagnosis above. ***Luster*:** See diagnosis above. ***Head*** (Fig. 45): as in description for genus above. ***Prothorax*.** Pronotum (Fig. 45) moderately broad, slightly narrower than head across eyes (WH/WP: 1.119), longer than head (LP/LH: 1.267), wider than longer (W/L: 1.254); markedly cordiform and convex, apical margin straight, lateral margin effaced with seta at anterior third on slightly raised area; apex markedly constricted; hind angle slightly produced and setose; median line feebly define as an infuscated line, transverse impressions punctate, punctures infuscated; surface smooth throughout. ***Pterothorax*.** Normal for genus, see description for genus above. Elytra markedly convex; at apical third twice as wide as head across eyes (WH/TW: 0.529) and pronotum (WP/TW: 0.472), longer than wide. Elytral interneurs evident as short rows of discontinuous, coarse and spaced punctures. Hind wings fully developed. ***Legs*.** Overall, normal for genus, see description for genus above. ***Abdominal sterna*.** Overall, normal for genus, see description for genus above. ***Male genitalia*.** Male unknown. ***Female genitalia*.** Not investigated, presumably similar to that of *Asklepia demiti* sp. n.

##### Dispersal potential.

These beetles are macropterous and probably capable of flight. They are moderately swift and agile runners.

##### Distribution.

(Fig. 78). This species has been found at only one location on the Río Uruguay drainage system. But that does not at all indicate its real distribution: as has been pointed out above, very small beetles are inadequately sampled, especially in the Neotropics.

##### Way of life.

See Erwin (1991) for a general description of the genus. Nothing specific about the way of life is known about this species.

##### Other specimens examined.

None.

##### Note.

The single known specimen is damaged, i.e., missing some appendages; however, there is the entire body with enough physical attributes and enough antennomeres to determine it represents a distinct species.

#### Asklepia marchantaria

Taxon classificationAnimaliaColeopteraCarabidae

Erwin & Zamorano sp. n.

http://zoobank.org/012D955A-C67A-4280-B602-38678E1EFA35

Marchantaria pattern-wing beetle Figs 46 , 70 , 78

##### Holotype.

**Brazil**, Amazonas, Rio Solimões, Ilha de Marchantaria, Lago Camaleão, 3.2488°S, 59.9556°W, 7m, 20 October 1981 (NMNH: ADIS # 000727, female).

##### Derivation of specific epithet.

The specific epithet, *marchantaria*, is a singular Latinized feminine noun in apposition, based on the name of the island on which these beetles are found.

##### Proposed english vernacular name.

Marchantaria pattern-wing beetle.

##### Diagnosis.

With the attributes of the genus *Asklepia* as described by Liebke (1938) and as noted above under the generic diagnosis, and medium-size for the genus (SBL = 2.599–2.828 mm). Adults with head brunneus, prothorax aurantiacus, elytral maculae aurantiacus; elytron brunneus with a triangular aurantiacus macula covering the left half of the apical proximal quadrant, a narrow aurantiacus macula vertically oriented in the medial proximal quadrant, a broad aurantiacus macula in the medial lateral quadrant, lateral and apical margins aurantiacus; metasternum and metasternum flavotestaceous, abdominal sterna with III-VI, and epipleuron flavotestaceous, abdominal sternum VII fuscous; legs flavotestaceous; antennal scape, pedicel and antennomere 3 testaceous, antennomeres 4-6 and basal half of 7 deeply infuscated, apical half of 7, 8–11 white. Dorsal surface devoid of microsculpture, surface luster very shiny. Pronotum markedly convex with lateral margin effaced except just anterior to hind angle and there a simple bead; hind angle barely prominent; median line feebly defined. Elytral interneurs effaced from greater part of the elytron surface, only evident as short discontinuous rows of scattered, coarse punctures unevenly spaced in the medial quadrants and along interneur 1.

##### Description.

(Fig. 46, 70). ***Habitus*:** (Fig. 46). ***Size*:** [See also Table 18] Medium-size for the genus; ABL = 2.80–3.41 mm, SBL = 2.599–2.828 mm, TW (total width) 1.324–1.582 mm, LP = 0.542–0.619 mm, WP = 0.670–0717 mm, LE = 1.613–1.809 mm. ***Color*:** See diagnosis above. ***Luster*:** See diagnosis above. ***Head*** (Fig. 46): as in description for genus above. ***Prothorax*.** Pronotum (Fig. 46) slightly broad, narrower than head across eyes (WH/WP, mean both sexes: 1.109), longer than head (LP/LH, mean both sexes: 1.382), wider than long (WP/LP, mean both sexes: 1.185); markedly cordiform and convex, lateral margin effaced with seta at anterior third on slightly raised area; apex markedly constricted; anterior angle feebly produced, hind angle slightly prominent and setose; median line feebly defined, transverse impressions punctate, punctures infuscated; surface smooth throughout. ***Pterothorax*.** Normal for genus, see description for genus above. Elytra moderately convex; at apical third twice as wide as head across eyes (WH/TW, mean both sexes: 0.529) and pronotum (WP/TW, mean both sexes: 0.476), longer than wide. Elytral interneur effaced from greater part of the elytron surface, only evident as short discontinuous rows of scattered, coarse punctures unevenly spaced in the medial quadrants and along interneur 1. Hind wings fully developed.

***Legs*.** Overall, normal for genus, see description for genus above. ***Abdominal sterna*.** Overall, normal for genus, see description for genus above. ***Male genitalia*** (Fig. 70, see Fig. 61 for attribute labels). Median lobe with phallobase short about a fourth the length of shaft, basal opening moderately large, oriented slightly oblique to shaft. Shaft broad, moderately curved ventrally, dorsally sclerotized except moderate-length ostium; in ventral aspect tapered toward narrowly rounded apex, in lateral aspect, a broadly rounded apex. Parameres missing. Endophallus with 2 preapical spines. ***Female genitalia*.** Not investigated, presumably similar to that of *Asklepia demiti* sp. n.

##### Dispersal potential.

These beetles are macropterous and probably capable of flight. They are moderately swift and agile runners.

##### Distribution.

(Fig. 78). This species has been found at only one location on a white-water lake shore within the Amazon River drainage system. But that does not at all indicate its real distribution: as has been pointed out above, very small beetles are inadequately sampled, especially in the Neotropics.

##### Way of life.

See Erwin (1991) for a general description. Adults of this species are active in the rainy season in the Varzea rainforest along the main course of the Rio Solimões. They occur at the Varzea forest edge on the floating macrophyte, *Eichornia crassipes* (Mart.) Solms.

##### Other specimens examined.

**Brazil**, Amazonas, Rio Solimões, Ilha de Marchantaria, Lago Camaleão, 3.2488°S, 59.9556°W, 7m, 1 October 1981 (J. Adis)(NMNH: ADIS # 000235, female paratype, ADIS # 000654, male paratype), 20 October 1981 (NMNH: ADIS # 001459, ADIS # 001429, ADIS # 001897, female paratypes; 4 November 1981 (NMNH: ADIS # 000864, ADIS # 001041, female paratypes). Non-paratypes and damaged: **Brazil**, Amazonas, Rio Solimões, Ilha de Marchantaria, Lago Camaleão, 3.2488°S, 59.9556°W, 7m, ADIS # 000780, ADIS # 001103, males, ADIS # 001282, ADIS # 001034, females, 14 August 1981 (NMNH: ADIS # 001663, female).

#### Asklepia marituba

Taxon classificationAnimaliaColeopteraCarabidae

Zamorano & Erwin sp. n.

http://zoobank.org/7DFCFA8B-D046-473E-BB61-E68FF399A356

Marituba pattern-wing beetle Figs 47 , 78

##### Holotype.

**Brazil**, Pará, Marituba, Ananindeua, 1.3712 °S, 48.3689°W, 10m, (F.M. Oliveira, P. Wygodzinsky)(AMNH: ADP132494, female).

##### Derivation of specific epithet.

The specific epithet, *marituba*, is a singular Latinized feminine noun in apposition, based on the name of the place where these beetles are found.

##### Proposed english vernacular name.

Marituba pattern-wing beetle.

##### Diagnosis.

With the attributes of the genus *Asklepia* as described by Liebke (1938) and as noted above under the generic diagnosis, and medium-size for the genus (SBL = 2.732 mm). Adults with head and prothorax aurantiacus, elytral maculae fulvous; elytron brunneus with scutellar area flavous, a broad flavous macula transversely oriented in the medial lateral quadrant, barely prolonged into the medial proximal quadrant, and a narrow oval flavous macula in the proximal apical quadrant, macula extended to the sutural area; metasternum, abdominal sterna III-VI, and epipleuron flavotestaceous, abdominal sternum VII infuscated; legs testaceous; antennal scape and pedicel fulvous, antennomeres 3–7 infuscated, 8–11 white. Dorsal surface devoid of microsculpture, surface luster very shiny. Pronotum markedly convex with lateral margin effaced except just anterior to hind angle and there a simple bead; hind angle slightly prominent; median line feebly defined. Elytral interneurs effaced from the greater part of the elytron, only visible as scattered coarse punctures.

##### Description.

(***Habitus***, Fig. 47). ***Size*:** [See also Table 19] Medium-size for the genus; ABL = 2.924 mm, SBL = 2.732 mm, TW (total width) = 0.820 mm, LP = 0.605 mm, WP = 0.768 mm, LE = 1.694 mm. ***Color*:** See diagnosis above. ***Luster*:** See diagnosis above. ***Head*** (Fig. 47): as in description for genus above. ***Prothorax*.** Pronotum (Fig. 47) moderately broad, as wide as head across eyes, (WH/WP: 1.069), longer than head (LP/LH: 1.398), slightly wider than long (WP/LP: 1.268); markedly cordiform and explanate, lateral margin beaded with seta at anterior third; base markedly constricted with medial lobe at base; anterior angles moderately produced, hind angle slightly produced, a right angle, and setose; median line markedly defined, basal and apical transverse impressions punctate, punctures infuscated; surface smooth throughout. ***Pterothorax*.** Normal for genus, see description for genus above. Elytra slightly convex; at apical third twice as wide as head across eyes (WH/TW: 0.521) and pronotum (WP/TW: 0.488), longer than wide. Elytral interneurs evident as rows of continuous punctures; punctures homogeneous. Hind wings fully developed. ***Legs*.** Overall, normal for genus, see description for genus above. ***Abdominal sterna*.** Overall, normal for genus, see description for genus above. ***Male genitalia*.** Male unknown. ***Female genitalia*.** Not investigated, presumably similar to that of *Asklepia demiti* sp. n.

##### Dispersal potential.

These beetles are macropterous and probably capable of flight; they are attracted to lights. They are moderately swift and agile runners.

##### Distribution.

(Fig. 78). This species has been found at only one location on a white-water system on the lower Río Amazonas. But that does not at all indicate its real distribution: as has been pointed out above, very small beetles are inadequately sampled, especially in the Neotropics.

##### Way of life.

See Erwin (1991) for a general description. Nothing is known about the way of life of this species.

##### Other specimens examined.

None.

#### Asklepia pakitza

Taxon classificationAnimaliaColeopteraCarabidae

Erwin & Zamorano sp. n.

http://zoobank.org/3537EF7A-F9CD-4395-A43B-57C286DE2EB8

Pakitza pattern-wing beetle Figs 48 , 71 , 78

##### Holotype.

**Perú**, Madre de Dios, BIOLAT Biological Station, Pakitza, Rio Manu, 11.9350°S, 71.3032°W, 329m, 18 & 21 February 1990 (T.L. Erwin, E. Pfuno S., F. Pfuno S.)(MUSM, ADP132466, male).

##### Derivation of specific epithet.

The specific epithet, *pakitza*, is a singular Latinized feminine noun in apposition, based on the name of the area in which these beetles are found. These beetles were collected under the auspices of the BIOLAT Program (see above under *Asklepia biolat* sp. n.).

##### Proposed english vernacular name.

Pakitza pattern-wing beetle.

##### Diagnosis.

With the attributes of the genus *Asklepia* as described by Liebke (1938) and as noted above under the generic diagnosis, and medium-sized for the genus (SBL = 2.635–2.683 mm). Adults with head and fuscous, prothorax aurantiacus, elytral maculae aurantiacus in some individuals; elytron brunneus with a narrow rectangular-shaped aurantiacus macula horizontally oriented in the lower half of medial lateral quadrant, scutellar area aurantiacus, sutural area of apical quadrant aurantiacus except for apical margin; metasternum, abdominal sterna III-VI, and epipleuron flavotestaceous, abdominal sternum VII infuscated; legs testaceous; antennal scape and pedicel testaceous, antennomeres 3-6 markedly infuscated, 8-11 white. Dorsal surface devoid of microsculpture, surface luster very shiny. Pronotum cordiform, feebly explanate, with medial lobe at base, lateral margin beaded; hind angle feebly prominent; median line markedly defined. Elytral interneurs effaced from greater part of the elytron surface, only evident as scattered punctures in the medial quadrants, as well as along interneur 1.

##### Description.

(Fig. 48, 71). ***Habitus*:** (Fig. 48). ***Size*:** [See also Table 20] Medium-size for the genus; ABL = 3.080–2.873 mm, SBL = 2.635–2.683 mm, TW (total width) 1.399–1.430 mm, LP = 0.564–0.572 mm, WP = 0.748–0.772 mm, LE = 1.701–1.743 mm. ***Color*:** See diagnosis above. ***Luster*:** See diagnosis above. ***Head*** (Fig. 48): as in description for genus above. ***Prothorax*.** Pronotum (Fig. 48) moderately broad, as wide as head across eyes (WH/WP, mean both sexes: 1.009), longer than head (LP/LH, mean both sexes: 1.539), wider than long (WP/LP, mean both sexes: 1.338); markedly cordiform and feebly explanate, lateral margin beaded with seta at anterior third; base markedly constricted with medial lobe at base; anterior angles feebly produced, hind angle markedly produced and setose; median line markedly define, apical transverse impressions punctate, punctures infuscated; surface smooth throughout. ***Pterothorax*.** Normal for genus, see description for genus above. Elytra slightly convex; at apical third twice as wide as head across eyes (WH/TW, mean both sexes: 0.542) and pronotum (WP/TW, mean both sexes: 0.538), longer than wide. Elytral interneurs effaced from greater part of the elytron surface, only evident as scattered punctures in the medial quadrants, as well as along interneur 1; elytron substantially transparent. Hind wings fully developed. ***Legs*.** Overall, normal for genus, see description for genus above. ***Abdominal sterna*.** Overall, normal for genus, see description for genus above. ***Male genitalia*** (Fig. 71, see Fig. 61 for attribute labels). Median lobe with phallobase very short about a fifth the length of shaft, basal opening small, oriented parallel to shaft. Shaft swollen at middle, slightly sinuate ventrally, dorsally sclerotized except for moderate-length ostium; in ventral aspect tapered toward rather rounded apex, in lateral aspect, a rounded apex. Parameres: left large and broad, right small and lobed; apex of left paramere lobate much longer than right paramere, about two-thirds the length of shaft (measured in left lateral aspect). Endophallus with one preapical spine. ***Female genitalia*.** Not investigated, presumably similar to that of *Asklepia demiti* sp. n.

##### Dispersal potential.

These beetles are macropterous and probably capable of flight. They are moderately swift and agile runners.

##### Distribution.

(Fig. 78). This species has been found at only one location on the shore of an isolated black-water of the Río Manu a part of the upper Amazon River drainage system. But that does not at all indicate its real distribution: as has been pointed out above, very small beetles are inadequately sampled, especially in the Neotropics.

##### Way of life.

See Erwin (1991) for a general description of the genus. Adults of this species are active in the dry season in wet leaf litter at the edge of a small lake.

##### Other specimens examined.

**Perú**, Madre de Dios, BIOLAT Biological Station, Pakitza, Rio Manu, 11.9350°S, 71.3032°W, 329m, 18 & 21 February 1990 (T.L. Erwin, E. Pfuno S., F. Pfuno S.) (NMNH, ADP132575, female paratype).

##### Notes.

See Erwin (1990) and Venable & Erwin (1997) for detailed trail maps of the BIOLAT Biodiversity Station. Adults of this species have a slightly explanate pronotal margin; however, they have the male endophallus of the *pulchripennis* group, thus the placement we suggest here. We have a specimen, in poor condition, that represents an undescribed species from Posto Jacaré, Brazil that also has a slightly explanate pronotal margin; however, it has the male endophallus of the *hilaris* group. With additional collecting and more specimens in the future, there may be an additional species group that we cannot, at present, define.

#### Asklepia paraguayensis

Taxon classificationAnimaliaColeopteraCarabidae

Zamorano & Erwin sp. n.

http://zoobank.org/717F296B-E122-4CF2-8DBB-F43574AB5E3D

**Figure 49–52.**
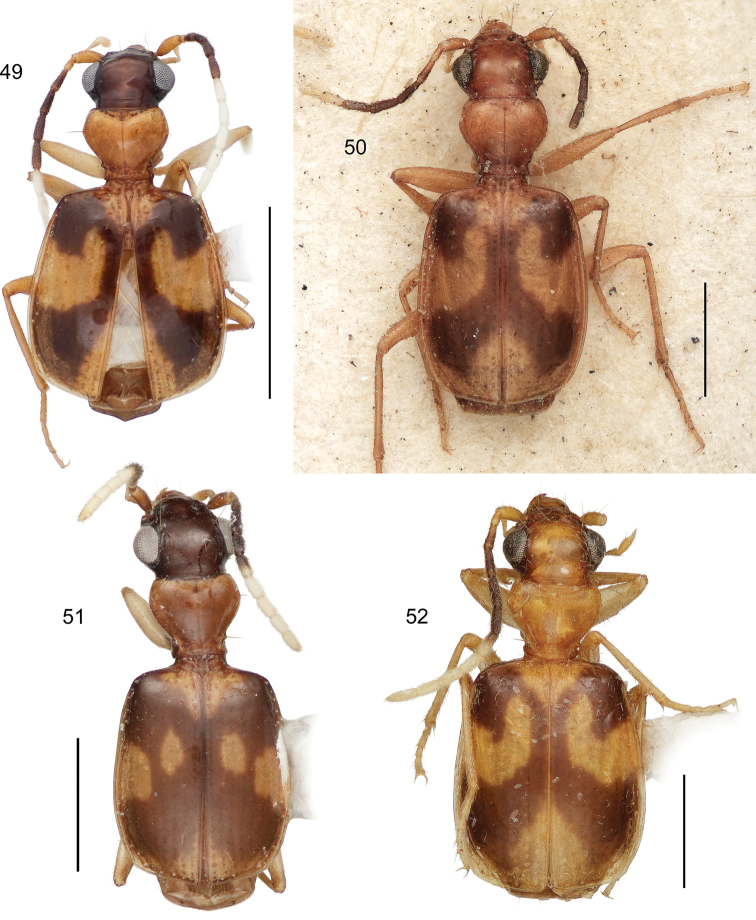
Digital Photo-illustrations, habitus, dorsal aspect of holotypes. **49** *Asklepia paraguayensis* Erwin & Zamorano, sp. n. ADP130038, San Lorenzo, Río Paraguay, Paraguay **50** *Asklepia pulchripennis* (Bates, 1871), **comb. n.**, ADP132531, Santarém, Río Tapajos, Brazil **51** *Asklepia samiriaensis* Zamorano & Erwin, sp. n. ADP051663, Boca del Río Samiria, Perú **52** *Asklepia stalametlitos* Zamorano & Erwin, sp. n. ADP132535, Guayamerín, Río Mamoré, Bolivia. Scale line = 1 mm.

Paraguayan pattern-wing beetle Figs 49 , 72 , 78

##### Holotype.

**Paraguay**, Central, San Lorenzo, Rio Paraguay, 25.385°S, 57.621°W, 52m, 23–24 November 1986 (J. Kochalka)(CMNH: ADP130038, male).

##### Derivation of specific epithet.

The specific epithet, *paraguayensis*, is a singular Latinized feminine noun in apposition, based on the name of the country in which these beetles are found.

##### Proposed english vernacular name.

Paraguayan pattern-wing beetle.

##### Diagnosis.

With the attributes of the genus *Asklepia* as described by Liebke (1938) and as noted above under the generic diagnosis, and small to medium-size for the genus (SBL = 2.478–2.769 mm). Adults with head fuscous, prothorax fulvous, elytral maculae fulvous or slightly aurantiacus in some individuals; elytron fuscous with a slender triangular flavous macula in the lower right corner of the proximal apical quadrant, broad flavous macula ending in hook crossing from medial lateral quadrant to right half of medial proximal quadrant, small triangular flavous macula in the upper right corner of basal proximal quadrant, apical and lateral margin fulvous, macula does not reach the humerus; metasternum fulvous, abdominal sterna III-VI, and epipleuron fulvous, abdominal sternum VII fuscous; legs flavotestaceous; antennal scape and pedicel testaceous, antennomeres 3-6 deeply infuscated, 7-11 white. Dorsal surface devoid of microsculpture, surface luster very shiny. Pronotum markedly convex and globose with lateral margin effaced except just anterior to hind angle and there a feeble bead; hind angle moderately prominent; anterior angles feebly produced; median line moderately defined. Elytral interneurs effaced from most of the elytron surface, only evident as short discontinuous rows of widely spaced coarse punctures.

##### Description.

(Fig. 49, 72). ***Habitus*:** (Fig. 49). ***Size*:** [See also Table 21] Medium-size to large for the genus; ABL = 3.002–3.372 mm, SBL = 2.478–2.769 mm, TW (total width) 1.397–1.598 mm, LP = 0.556–0.751 mm, WP = 0.703–0.861 mm, LE = 1.623–2.024 mm. ***Color*:** See diagnosis above. ***Luster*:** See diagnosis above. ***Head*** (Fig. 49): as in description for genus above. ***Prothorax*.** Pronotum (Fig. 49) moderately broad, as wide as head across eyes (WH/WP, mean both sexes: 1.072), longer than head (LP/LH, mean both sexes: 1.397), wider than long (W/L, mean both sexes: 1.693); markedly cordiform and rounded, lateral margin effaced with seta at anterior third on slightly raised area; apex markedly constricted; anterior angle feebly produced, hind angle slightly produced and setose; median line moderately defined, apical transverse impressions punctate, punctures infuscated; surface smooth throughout. ***Pterothorax*.** Normal for genus, see description for genus above. Elytra moderate convex; at apical third twice as wide as head across eyes (WH/TW, mean both sexes: 0.526) and pronotum (WP/TW, mean both sexes: 0.491). Elytral interneurs effaced from most of the elytron surface, only evident as short discontinuous rows of widely spaced coarse punctures. Hind wings fully developed. ***Legs*.** Overall, normal for genus, see description for genus above. ***Abdominal sterna*.** Overall, normal for genus, see description for genus above. ***Male genitalia*** (Fig. 72, see Fig. 61 for attribute labels). Median lobe with phallobase moderate, about a fourth the length of shaft, basal opening large, oriented oblique to shaft. Shaft broad, slightly curved ventrally, dorsally sclerotized except for short ostium; in ventral aspect tapered toward rather narrowly acute apex, in lateral aspect, a slightly rounded apex. Left paramere (missing) (**lp)**, probably very large and broad, right small and triangular; apex of left paramere probably lobate and much longer than right paramere (**rp**). Internal sac with one median spine, one long distal spine. ***Female genitalia*.** Not investigated, presumably similar to that of *Asklepia demiti* sp. n.

##### Dispersal potential.

These beetles are macropterous and capable of flight; they are attracted to lights. They are moderately swift and agile runners.

##### Distribution.

(Fig. 78). This species has been found at only one location along the white-water river of the middle Río Paraguay drainage system. But that does not at all indicate its real distribution: as has been pointed out above, very small beetles are inadequately sampled, especially in the Neotropics.

##### Way of life.

See Erwin (1991) for a general description. Adults of this species are active in the rainy season on margins of large rivers. They likely occur in wet leaf litter on wet soil in swales off to the side of the main river course.

##### Other specimens examined.

**Paraguay**, Central, San Lorenzo, Rio Paraguay, 25.385°S, 57.621°W, 52m, 23–24 November 1986 (J. Kochalka) (CMNH: ADP132769, ADP132767, male paratypes; ADP132765, female paratype).

#### Asklepia pulchripennis

Taxon classificationAnimaliaColeopteraCarabidae

(Bates) 1871 comb. n.

Beautiful pattern-wing beetle Fig. 50 , 78

Eucaerus pulchripennis Bates, 1871:79.

##### Holotype.

Brazil, Pará, Santarém, Rio Tapajos, 2.4079°S, 54.7969°W, 30m, (H.W.Bates) (MNHP: ADP132531, female). Specimen labeled “Holotype” by George E. Ball in 1972.

##### Derivation of specific epithet.

The specific epithet, *pulchripennis*, is a noun in apposition that adequately describes this species with pretty (pulchri-) patterned elytra (-pennis) contrasted with shiny aurantiacus head and pronotum.

##### Proposed english vernacular name.

Beautiful pattern-wing beetle.

##### Diagnosis.

With the attributes of the genus *Asklepia* as described by Liebke (1938) and as noted above under the generic diagnosis, and medium-sized for the genus (SBL = 2.718 mm). Adults with head and prothorax fulvous, elytral maculae fuscous; elytron fulvous with a fuscous macula in the basal lateral quadrant barely trespassing proximal quadrant and a fuscous macula in the apical lateral quadrant extending to the upper half of apical proximal quadrant; lower half of basal and medial quadrant’s sutural area fuscous; metasternum, abdominal sterna III-VI, and epipleuron flavous, abdominal sternum VII fuscous; legs testaceous; antennal scape, pedicel, and antennomere 3 testaceous, antennomeres 4-6 deeply infuscated, 7-11 white. Dorsal surface devoid of microsculpture, surface luster very shiny. Pronotum markedly convex with lateral margin effaced except just anterior to hind angle and there a simple bead; anterior angles feebly produced, hind angle slightly prominent, median line markedly defined. Elytral interneurs effaced from the greater part of the elytron surface, only evident as pale coarse punctures in the apical proximal quadrant and with scattered punctures in the medial quadrants, as well as along entire interneurs 1 and 2.

##### Description.

(***Habitus***, Fig. 50). ***Size*:** [See also Table 22] Medium-size for the genus; ABL = 3.019 mm, SBL = 2.718 mm, TW (total width) 1.366 mm, LP = 0.584 mm, WP = 0.700 mm, LE = 1.732 mm. ***Color*:** See diagnosis above. ***Luster*:** See diagnosis above. ***Head*** (Fig. 50): as in description for genus above. ***Prothorax*.** Pronotum (Fig. 50) moderately broad, as wide as head across eyes (WH/WP: 1.0987), longer than head (LP/LH: 1.454), about as longer than wide (WP/LP: 1.198); markedly cordiform and convex, lateral margin effaced with seta at anterior third on slightly raised area; apex markedly constricted; anterior angles feebly produced, hind angle slightly produced and setose; median line feebly defined, basal and apical transverse impressions punctate, punctures infuscated; surface smooth throughout. ***Pterothorax*.** Normal for genus, see description for genus above. Elytra slightly convex; at apical third twice as wide as head across eyes (WH/TW: 0.563) and pronotum (WP/TW: 0.512), longer than wide. Elytral interneurs effaced from the greater part of the elytron surface, only evident as pale coarse punctures of the apical proximal quadrant and scattered punctures on medial quadrants, as well as along entire interneurs 1 and 2. Hind wings fully developed. ***Legs*.** Overall, normal for genus, see description for genus above. ***Abdominal sterna*.** Overall, normal for genus, see description for genus above. ***Male genitalia*.** Unknown. ***Female genitalia*.** Not investigated, presumably similar to that of *Asklepia demiti* sp. n.

##### Dispersal potential.

These beetles are macropterous and probably capable of flight. They are moderately swift and agile runners.

##### Distribution.

(Fig. 78). This species has been found at only one location on a clear-water system of the middle Amazon River drainage system. But that does not at all indicate its real distribution: as has been pointed out above, very small beetles are inadequately sampled, especially in the Neotropics.

##### Way of life.

See Erwin (1991) for a general description. Bates reported finding the holotype on the shore of the Rio Tapajos, a gravelly clear water tributary of the Rio Amazonas.

##### Other specimens studied.

None.

#### Asklepia samiriaensis

Taxon classificationAnimaliaColeopteraCarabidae

Zamorano & Erwin sp. n.

http://zoobank.org/3B4E42CE-FDE3-43D2-9706-00DF613D2FF2

Samiria pattern-wing beetle Figs 51 , 78

##### Holotype.

**Perú**, Loreto, Boca del Río Samiria, 1 km SW Vigilante post No. 1, 4.5005°S, 74.0659°W, 99m, 16 August 1991 (T.L. Erwin, M.G. Pogue)(MUSM: ADP051663, male).

##### Derivation of specific epithet.

The specific epithet, *samiriaensis*, is a singular Latinized feminine noun in apposition, based on the name of the river near which these beetles were found.

##### Proposed english vernacular name.

Samiria pattern-wing beetles.

##### Diagnosis.

With the attributes of the genus *Asklepia* as described by Liebke (1938) and as noted above under the generic diagnosis, and small-sized for the genus (SBL = 2.436 mm). Adults with head brunneus, prothorax flavotestaceous, elytral maculae fulvous; elytron fuscous with a small triangular flavous macula in the basal proximal quadrant reaching the sutural area, medial lateral quadrant fulvous, an enclosed ocellate flavous macula in the middle of medial proximal quadrant, a triangular flavous macula in the apical proximal quadrant, lateral and apical margins fulvous; abdominal sterna III-VI, and epipleuron fulvous, abdominal sternum VII fuscous; legs fulvous; antennal scape, pedicel and antennomere 3 testaceous, antennomeres 4-6 and basal half of 7 deeply infuscated, apical half of 7 and 8-11 white. Dorsal surface devoid of microsculpture, surface luster very shiny. Pronotum markedly convex with lateral margin effaced except just anterior to hind angle and there a simple bead; hind angle feebly produced, median line feebly defined. Elytral interneurs effaced from greater part of elytron surface, only evident as short discontinuous rows of spaced punctures at apical and basal sutural area, punctures fuscous.

##### Description.

(***Habitus***, Fig. 51). ***Size*:** [See also Table 23] Medium-size for the genus; ABL = 2.853 mm, SBL = 2.436 mm, TW (total width) 1.274 mm, LP = 0.537 mm, WP = 0.613 mm, LE =1.503 mm. ***Color*:** See diagnosis above. ***Luster*:** See diagnosis above. ***Head*** (Fig. 51): as in description for genus above. ***Prothorax*.** Pronotum (Fig. 51) slightly broad, slightly narrower than head across eyes (WH/WP: 1.188), longer than head (LP/LH: 1.357), about as wide as long (W/L: 1.141); markedly cordiform and rounded, lateral margin effaced with seta at anterior third on slightly raised area; apex markedly constricted; anterior angles feebly produced, hind angle slightly produced and setose, median line feebly defined, basal margin fuscous; surface smooth throughout. ***Pterothorax*.** Normal for genus, see description for genus above. Elytra moderately convex; twice as wide as head across eyes (WH/TW: 0.571) and pronotum (WP/TW: 0.481), longer than wide. Elytral interneurs effaced from greater part of the elytron surface, only evident as short discontinuous rows of spaced punctures at apical and basal sutural area, punctures fuscous; elytron substantially transparent. Hind wings fully developed. ***Legs*.** Overall, normal for genus, see description for genus above. ***Abdominal sterna*.** Overall, normal for genus, see description for genus above. ***Male genitalia*.** Not investigated due to the fragile nature of the holotype. ***Female genitalia*.** Unknown.

##### Dispersal potential.

These beetles are macropterous and probably capable of flight. They are moderately swift and agile runners.

##### Distribution.

(Fig. 78). This species has been found at only one location on a black-water system of the upper Amazon River drainage system. But that does not at all indicate its real distribution: as has been pointed out above, very small beetles are inadequately sampled, especially in the Neotropics.

##### Way of life.

See Erwin (1991) for a general description. Adults of this species are active in the rainy season and occur in open grassy marshes.

##### Other specimens examined.

None.

#### Asklepia stalametlitos

Taxon classificationAnimaliaColeopteraCarabidae

Zamorano & Erwin sp. n.

http://zoobank.org/2A7E97C1-458D-42D3-9D17-C5B41DD22579

Honey-drop pattern-wing beetle Fig. 52 , 78

##### Holotype.

**Bolivia**, Beni, Guayamer, Rio Mamoré, 10.8033°S, 65.3476°W, 118m, 24 August 1964 (J.K. Bouseman, L. Lussenhop)(AMNH: ADP132535, female).

##### Derivation of specific epithet.

The specific epithet, *stalametlitos*, is derived from the Greek, σταλα (stalas) = drop, μηλλιτοσ (melitos) = of honey, drop of honey, and used as a noun in apposition in reference to the golden color of the elytra of these beetles.

##### Proposed english vernacular name.

Honey-drop pattern-wing beetles.

##### Diagnosis.

With the attributes of the genus *Asklepia* as described by Liebke (1938) and as noted above under the generic diagnosis, and medium-sized for the genus (SBL = 2.815 mm). Adults with head and prothorax flavous, elytral maculae flavous; elytron (cf. Fig. 30) fuscous with triangular flavous macula on proximal basal quadrant, maculae reach the sutural area, medial quadrants largely fulvous, medial sutural area fuscous, proximal apical quadrant with triangular macula flavous macula on proximal basal quadrant reaching the sutural area, medial quadrants largely fulvous without reaching the sutural area, proximal apical quadrant with triangular flavous macula, testaceous, antennomere 3 aurantiacus, antennomeres 4-6 and basal half of 7 deeply infuscated, apical half of 7, 8-11 white. Dorsal surface devoid of microsculpture, surface luster very shiny. Pronotum markedly convex with lateral margin effaced except just anterior to hind angle and there a simple bead; hind angle moderately prominent. Elytral interneurs effaced from the greater part of the elytron surface, only evident as pale spots on apical proximal quadrant and scattered punctures on medial quadrants; elytron substantially transparent.

##### Description.

(***Habitus***, Fig. 52). ***Size*:** [See also Table 24] Medium-size for the genus; ABL = 3.043 mm, SBL = 2.815 mm, TW (total width) 1.467 mm, LP = 0.597 mm, WP = 0.751 mm, LE = 1.788 mm. ***Color*:** See diagnosis above. ***Luster*:** See diagnosis above. ***Head*** (Fig. 52): as in description for genus above. ***Prothorax*.** Pronotum (Fig. 52) moderately broad, as wide as head across eyes (WH/WP: 1.089), longer than head (LP/LH: 1.388), wider than longer (W/L: 1.024); markedly cordiform and convex, lateral margin effaced with seta at anterior third on slightly raised area; apex markedly constricted; anterior angle feebly produced, hind angle slightly produced and setose; devoid of median line and transverse impression; surface smooth throughout. ***Pterothorax*.** Normal for genus, see description for genus above. Elytra moderately convex; twice as wide as head across eyes (WH/TW: 0.557) and pronotum (WP/TW: 0.512), longer than wide. Elytral interneurs effaced from the greater part of the elytron surface, only evident as pale spots on apical proximal quadrant and scattered punctures on medial quadrants. Hind wings fully developed. ***Legs*.** Overall, normal for genus, see description for genus above. ***Abdominal sterna*.** Overall, normal for genus, see description for genus above. ***Male genitalia*.** Male unknown. ***Female genitalia*.** Not investigated, presumably similar to that of *Asklepia demiti* sp. n.

##### Dispersal potential.

These beetles are macropterous and probably capable of flight. They are moderately swift and agile runners.

##### Distribution.

(Fig. 78). This species has been found at only one location on a white-water system of the upper Amazon River drainage system. But that does not at all indicate its real distribution: as has been pointed out above, very small beetles are inadequately sampled, especially in the Neotropics.

##### Way of life.

See Erwin (1991) for a general description. Adults of this species are active in the rainy season along a large river.

##### Other specimens examined.

None.

#### Asklepia strandi

Taxon classificationAnimaliaColeopteraCarabidae

Liebke, 1938

**Figure 53–55.**
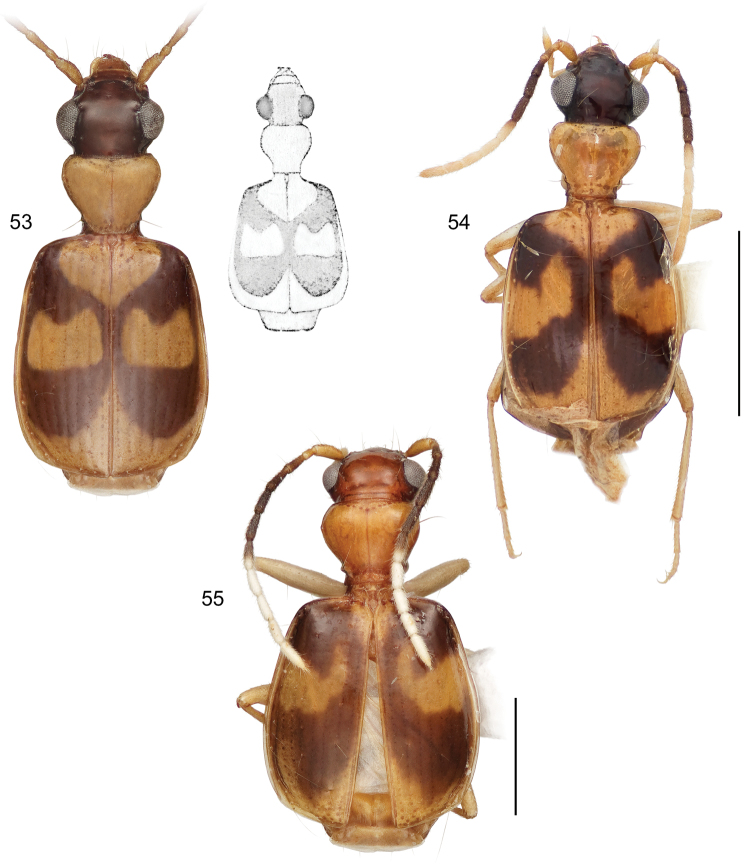
Digital Photo-illustrations, habitus, dorsal aspect of holotypes. **53** *Asklepia strandi* Liebke, 1938, Guyana, ABL = 3.00 mm (from Liebke, 1938:114). Liebke’s illustration of *Asklepia strandi* Liebke. The single specimen Liebke described was likely lost during World War II. We have seen no specimens from Guyana, nor any specimens matching this illustration from anywhere **54** *Asklepia surinamensis* Erwin & Zamorano, sp. n. ADP130040, l’Hermitage, Surinam River, Surinam **55** *Asklepia vigilante* Erwin & Zamorano, sp. n. ADP051642, Boca del Río Samiria, Perú. Scale line = 1 mm.

Strand’s pattern-wing beetle Figs 53 , 78

Asklepia strandi *Asklepia Strandi* Liebke, 1938:113.

##### Holotype.

**Guyana** (not seen by us, not listed by Mroczkowski (1960) as being in the collections of the Polish Academy of Sciences in Warsaw; it was probably lost during World War II).

##### Derivation of specific epithet.

The specific epithet, *strandi*, is an eponym, noun in apposition, genitive case, based on the family name of Professor Dr. Embrik Strand, Norwegian Arachnologist, who spent his later years as a Professor in Germany.

##### Proposed english vernacular name.

Strand’s pattern-wing beetle.

##### Diagnosis.

With the attributes of the genus *Asklepia* as described by Liebke (1938) and as noted above under the generic diagnosis, and large-sized for the genus (ABL = 3.0). The following is Liebke’s original description.

“**Asklepia Strandi** n. sp. (Fig. 113).

Hellgelbbraun, Kopf dunkelbraun. Spitze des vierten Fühlerglie-

Des, fünftes und sechstes Glied vollständig dunkelbraun, siebentes

bis elftes Glied gelblichweiss. Flügeldecken zum grössten Teil dun-

kelgelbbraun, ein grosser Skutellarfleck. *2* Scheibenflecke, der Spit-

zenrand in breitem Umfange und der Seitenrand bis fast zur Schulter

hinauf schmal blassgelb. Der vordere Teil der Epipleuren, die Hin-

terbrust und die Rückenringe des Hinterleibes sind dunkelbraun.

Kopf rundlich, kurz, stark gewölbt, mit sehr grossen, stark ge-

wölbten, stark vorstehenden Augen, dieselben sind ungewöhnlich

grob fazettiert. Schläfen sehr kurz, leicht verengt. Kopfschild voll-

kommen glatt und unpunktiert. Hals sehr kurz und sehr dick. Hals-

schild stark herzförmig, etwa ^1^/_6_ breiter als lang, kaum merklich

schmäler als der Kopf, stark gewölbt: Vorderrand gerade, Vorder-

winkel breit abgerundet. Seiten vor der Mitte stark gerundet erwei-

tert, sodann stark verengt, kurz vor der Basis am stärksten einge-

schnürt; Basalwinkel spitz, etwas vorspringend; Basalrand gerade.

Seiten undeutlich gerandet, Rand im Vorderdrittel vollkommen ver-

loschen. Mittellängslinie schwach eingedrückt. Ganze Oberseite

vollkommen glatt, unpunktiert. Am Seitenrand kurz vor der Mitte

eine lange Borste. Flügeldecken kurzviereckig, stark gewölbt, nach

hinten stark verbreitert, etwa ein Viertel länger als der Vorderkör-

per, an der breitesten Stelle (im zweiten Drittel) kaum schmäler

als lang; Schultern schräg abfallend. Schulterwinkel abgerundet, an

der Spitze gerade abgestutzt, Spitzenrand gerade, Spitzenaussen-

winkel breit abgerundet. Epipleuren der Flügeldecken an der Schul-

ter sehr breit, dann aber, in etwa ^1^/_3_ der Länge, verschmälern sie

sich plötzlich sehr stark, behalten höchstens noch ^1^/_5_ der vorderen

Breite, um dann allmählich spitz auszulaufen. Punktstreifen nicht

erkennbar, wohl aber sind schwach gewölbte Zwischenräume er-

kennbar, diese sind g1att und unpunktiert, jedoch im Grunde äusserst

fein netzmaschig gerunzelt; auf dem 3., 5. und 7. stehen je 3-4 lange

aufrechte Borstecn. Länge 3 mm.

Ein Stück. bezettelt «Guyana », in meiner Sammlung.

Dem Jubilar, Professor Dr. **Embrik Strand** gewidmet.”

##### Note.

From Liebke’s drawing, enhanced here (Fig. 53), we can classify this species in the *pulchripennis* species group because the pronotum is not laterally explanate, an attribute that is always associated with an aedeagal endophallus having only two spines. We have seen no specimens from Guyana, nor any specimens matching this illustration from anywhere. The distinguishing attribute is the pale elytral margin extended from the apex into quadrant A (Fig. 53). A neotype will be needed upon discovery of more specimens from Guyana that match Liebke’s description and illustration.

**Figure 56.**
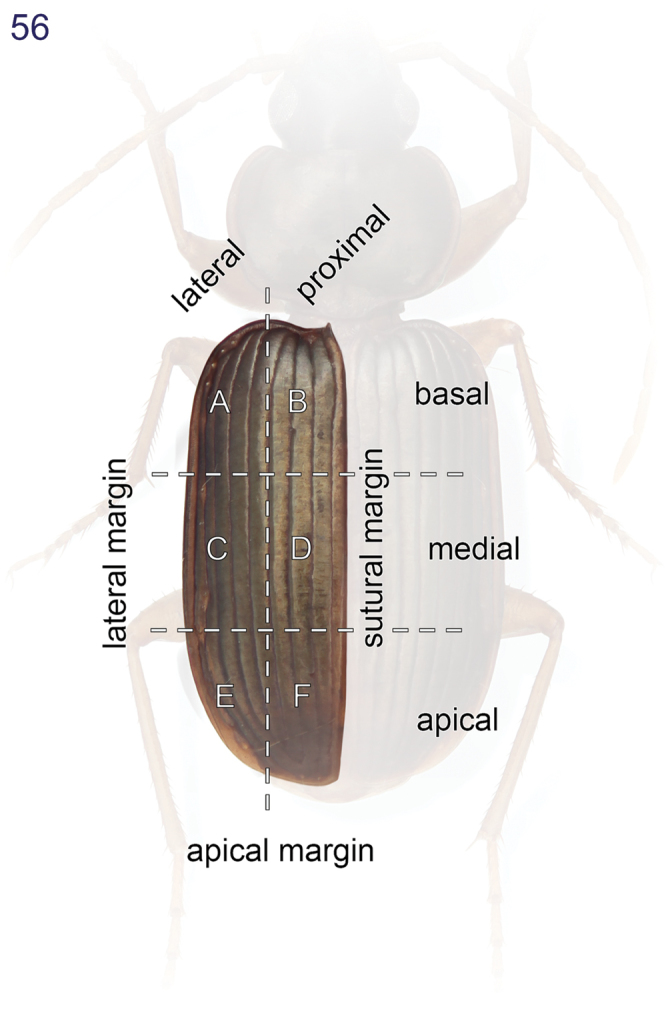
Image of elytron with diagram of quadrants used in the descriptions.

#### Asklepia surinamensis

Taxon classificationAnimaliaColeopteraCarabidae

Zamorano & Erwin sp. n.

http://zoobank.org/EEC60AF5-9DBC-4304-AE84-901AFEF674FD

Surinam pattern-wing beetle Figs 54 , 73 , 78

##### Holotype.

**Surinam**, Paramaribo, l’Hermitage, Surinam River, 5.8182°N, 55.1639°W, 2m, 14 July 1969 (N. Nieser)(CMNH: ADP130040, male).

##### Derivation of specific epithet.

The specific epithet, *surinamensis*, is a singular Latinized feminine noun in apposition, based on the name of the country in which these beetles are found.

##### Proposed english vernacular name.

Surinam pattern-wing beetle.

##### Diagnosis.

With the attributes of the genus *Asklepia* as described by Liebke (1938) and as noted above under the generic diagnosis, and medium-size to large-size for the genus (SBL = 2.841–2.99 mm). Adults with head fuscous, prothorax fulvous, elytral maculae fulvous or slightly aurantiacus in some individuals; elytron fuscous with a triangular flavous macula in the upper right corner of the proximal basal quadrant, broad flavous macula ending in hook crossing from medial lateral quadrant to left half of medial proximal quadrant, triangular flavous macula crossing from the lower right corner of apical proximal quadrant to lower half of apical lateral quadrant, apical and lateral margin fulvous; metepisternum fuscous, metasternum fuscous laterally flavous medially, abdominal sterna with III-VI, and epipleuron fulvous, abdominal sternum VII fuscous; legs flavotestaceous; antennal scape, pedicel and antennomere 3 testaceous, antennomeres 4-6 deeply infuscated, 7-11 white. Dorsal surface devoid of microsculpture, surface luster very shiny. Pronotum markedly convex and globose with lateral margin effaced except just anterior to hind angle and there a feeble bead; hind angle moderately prominent; anterior angles feebly produced; median line feebly defined. Elytral interneurs evident as continuous rows of widely spaced coarse punctures.

##### Description.

(Fig. 54, 73). ***Habitus*:** Fig. 54). ***Size*:** [See also Table 25] Medium-size to large for the genus; ABL = 3.002–3.372 mm, SBL = 2.841–2.992 mm, TW (total width) 1.420–1.584 mm, LP = 0.575–0.626 mm, WP = 0.795–0.836 mm, LE = 1.709–1.803 mm. ***Color*:** See diagnosis above. ***Luster*:** See diagnosis above. ***Head*** (Fig. 54): as in description for genus above. ***Prothorax*.** Pronotum (Fig. 54) slightly broad, as wide as head across eyes (WH/WP, mean both sexes: 1.101), length about the same (LP/LH, mean both sexes: 1.093), wider than long (W/L, mean both sexes: 1.367); markedly cordiform and rounded, lateral margin effaced with seta at anterior third on slightly raised area; apex markedly constricted; anterior angle feebly produced, hind angle slightly produced and setose; median line feebly defined, apical transverse impressions punctate, punctures infuscated; surface smooth throughout. ***Pterothorax*.** Normal for genus, see description for genus above. Elytra moderate convex; at apical third twice as wide as head across eyes (WH/TW, mean both sexes: 0.542) and pronotum (WP/TW, mean both sexes: 0.4923). Elytral interneurs evident as continuous rows of widely spaced coarse punctures, punctures infuscated, interneurs continuous along length of entire elytron. Hind wings fully developed. ***Legs*.** Overall, normal for genus, see description for genus above. ***Abdominal sterna*.** Overall, normal for genus, see description for genus above. ***Male genitalia*** (Fig. 73, see Fig. 61 for attribute labels). Median lobe with phallobase moderate in length, about a fifth the length of shaft, basal opening moderate, oriented oblique to shaft. Shaft broad, slightly curved ventrally, dorsally sclerotized except for short ostium; in ventral aspect tapered toward broadly rounded apex, in lateral aspect, a rounded blunt apex. Left paramere very large and broad, right paramere moderately large and triangular; apex of left paramere lobate much, longer than right paramere about two-thirds the length of shaft (measured in left lateral aspect). Endophallus with one small median spine, and one very large distal spine. ***Female genitalia*.** Not investigated, presumably similar to that of *Asklepia demiti* sp. n.

##### Dispersal potential.

These beetles are macropterous and capable of flight; they are attracted to lights. They are moderately swift and agile runners.

##### Distribution.

(Fig. 78). This species has been found at only one location on the white-water system of the middle Surinam River drainage system. But that does not at all indicate its real distribution: as has been pointed out above, very small beetles are inadequately sampled, especially in the Neotropics.

##### Way of life.

See Erwin (1991) for a general description. Adults of this species are active in July.

##### Other specimens examined.

**Surinam**, Paramaribo, l’Hermitage, Surinam River, 5.8182°N, 55.1639°W, 2m, 10 July 1969 (N. Nieser)(CMNH: ADP130042, ADP132761, female paratypes; ADP132763, ADP132759, ADP132757, ADP133569 male paratypes).

#### Asklepia vigilante

Taxon classificationAnimaliaColeopteraCarabidae

Erwin & Zamorano sp. n.

http://zoobank.org/47519645-5E47-4EA7-A8E2-A3B30C7E251B

Vigilante pattern-wing beetle Figs 55 , 74 , 78

##### Holotype.

**Perú**, Loreto, Boca del Río Samiria, 1 km SW Vigilante post No. 1, 4.5005°S, 74.0659°W, 99m, 16 August 1991 (T.L. Erwin, M.G. Pogue)(MUSM: ADP051642, female).

##### Derivation of specific epithet.

The specific epithet, *vigilante*, is a singular Latinized feminine noun in apposition, based on the name of the place near which these beetles are found.

##### Proposed english vernacular name.

Vigilante pattern-wing beetle.

##### Diagnosis.

With the attributes of the genus *Asklepia* as described by Liebke (1938) and as noted above under the generic diagnosis, and medium-size to large-size for the genus (SBL = 2.589–3.259 mm). Adults with head aurantiacus, prothorax fulvous, elytral maculae fulvous or aurantiacus in some individuals; elytron fuscous with a triangular flavous macula in the lower right corner of the proximal apical quadrant, broad flavous macula ending in hook crossing from medial lateral quadrant to right half of medial proximal quadrant, triangular flavous macula in the upper right corner of apical proximal quadrant, apical and lateral margin fulvous; metasternum fulvous, abdominal sterna with III-VI, and epipleuron fulvous, abdominal sternum VII fuscous; legs flavotestaceous; antennal scape and pedicel testaceous, antennomeres 3-6 and basal half of 7 deeply infuscated, apical half of 7 and 8-11 white. Dorsal surface devoid of microsculpture, surface luster very shiny. Pronotum markedly convex with lateral margin effaced except just anterior to hind angle and there a simple bead; hind angle moderately prominent; anterior angles feebly produced; median line feebly defined. Elytral interneurs evident as short discontinuous rows of widely spaced coarse punctures, interneurs effaced in the medial quadrants.

##### Description.

(Fig. 55, 74). ***Habitus*:** (Fig. 55). ***Size*:** [See also Table 26] Medium-size to large for the genus; ABL = 3.002–3.372 mm, SBL = 2.589–3.259 mm, TW (total width) 1.397–1.598 mm, LP = 0.556–0.751 mm, WP = 0.703–0.861 mm, LE = 1.623–2.024 mm. ***Color*:** See diagnosis above. ***Luster*:** See diagnosis above. ***Head*** (Fig. 55): as in description for genus above. ***Prothorax*.** Pronotum (Fig. 55) slightly broad, about as wide as head across eyes (WH/WP, mean both sexes: 1.051), longer than head (LP/LH, mean both sexes: 1.436), about as wide as long (WP/LP, mean both sexes: 1.209); markedly cordiform and rounded, lateral margin effaced with seta at anterior third on slightly raised area; apex markedly constricted; anterior angle feebly produced, hind angle slightly produced and setose; median line feebly defined, apical transverse impressions punctate, punctures infuscated; surface smooth throughout. ***Pterothorax*.** Normal for genus, see description for genus above. Elytra moderate convex; at apical third twice as wide as head across eyes (WH/TW, mean both sexes: 0.534) and pronotum (WP/TW, mean both sexes: 0.508), longer than wide. Elytral interneurs evident as short discontinuous rows of widely spaced coarse punctures, interneurs effaced in the medial quadrants. Hind wings fully developed. ***Legs*.** Overall, normal for genus, see description for genus above. ***Abdominal sterna*.** Overall, normal for genus, see description for genus above. ***Male genitalia*** (Fig. 74, see Fig. 61 for attribute labels). Median lobe with phallobase short about a fourth the length of shaft, basal opening large, oriented parallel to shaft. Shaft broad, moderately curved ventrally, dorsally sclerotized except for short ostium; in ventral aspect tapered toward rather broadly acute apex, in lateral aspect, a rounded apex. Left paramere very large and broad, right small and triangular; apex of left paramere lobate much longer than right paramere, about half the length of shaft (measured in left lateral aspect). Endophallus with 2 preapical spines, distal one very large. ***Female genitalia*.** Not investigated, presumably similar to that of *Asklepia demiti* sp. n.

##### Dispersal potential.

These beetles are macropterous and probably capable of flight. They are moderately swift and agile runners.

##### Distribution.

(Fig. 78). This species has been found at only one location on the black-water system of the upper Amazon River drainage system. But that does not at all indicate its real distribution: as has been pointed out above, very small beetles are inadequately sampled, especially in the Neotropics.

##### Way of life.

See Erwin (1991) for a general description. Adults of this species are active in the rainy season in Igapó rainforest. They occur in wet leaf litter on wet soil in swales off to the side of the main river course and in open grassy marshes with some standing water.

##### Other specimens examined.

**Perú**, Loreto,1 km SW Boca del Rio Samiria, Vigilante post No. 1, 4.5005°S, 74.0659°W, 99m, 5 May 1990 (T.L. Erwin)(NMNH: ADP132520, female paratype),14 August 1991 (T.L. Erwin)(NMNH: ADP067302, female paratype, ADP067301, male paratype),16 August 1991 (T.L. Erwin, M.G. Pogue) (NMNH: ADP051665, male paratype).

**Figure 57–62.**
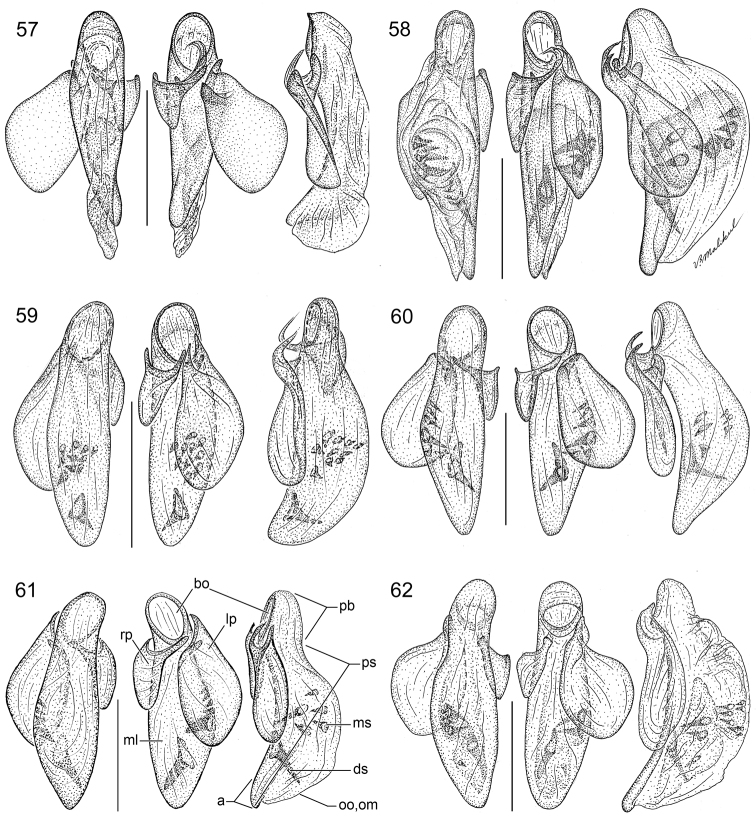
Illustrations, male aedeagus, dorsal, ventral, left lateral aspects. **57** *Asklepia geminata* (Bates, 1871) ADP109186, Río Samiria, Boca Caño Inglés Camp, Perú **58** *Asklepia campbellorum* Zamorano & Erwin, sp. n. ADP109196, 20 km SW Manaus, Brazil **59** *Asklepia demiti* Erwin & Zamorano, sp. n. ADP132585, Rio Demiti, Brazil **60** *Asklepia grammechrysea* Zamorano & Erwin, sp. n. ADP052565, Río Sucusari, Perú **61** *Asklepia laetitia* Zamorano & Erwin, sp. n. ADP109190, Leticia, Colombia. Legend: **a** apical area; **bl** basal lobe; **bo** basal orifice; **lp** left paramere; **ml** median lobe; **om** ostial membrane; **oo** ostial opening; **rp** right paramere; **sh** shaft; **pb** phallobase; **ps** phallobase shaft; **ms** medial spine; **ds** distal spine **62** *Asklepia lebioides* (Bates, 1871), comb. n., ADP109208, Rio Demiti, Brazil. Scale line = 0.25 mm.

**Figure 63–68.**
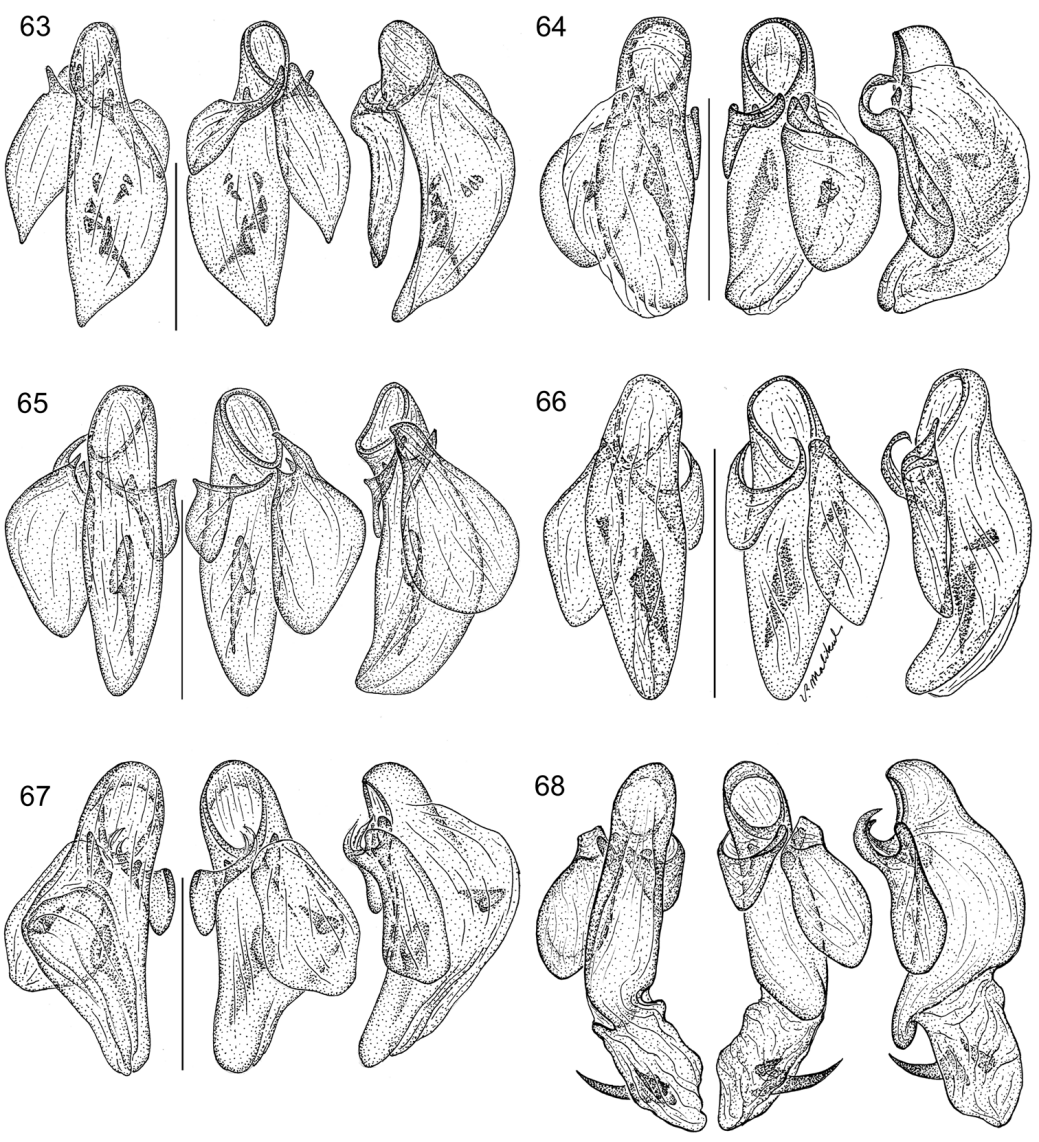
Illustrations, male aedeagus, dorsal, ventral, left lateral aspects. **63** *Asklepia matomena* Zamorano & Erwin, sp. n. ADP132527, 20 km SW Manaus, Brazil **64** *Asklepia adisi* Erwin & Zamorano, sp. n. Adis # 001335, Ilha de Marchantaria, Lago Camaleão, Brazil **65** *Asklepia biolat* Erwin & Zamorano, sp. n. ADP132480, Pakitza, Perú **66** *Asklepia bracheia* Zamorano & Erwin, sp. n. ADP067304, Boca del Río Samiria Perú **67** *Asklepia ecuadoriana* Erwin & Zamorano, sp. n. ADP132468, Limoncocha, Ecuador. Endophallus not everted **68** *Asklepia ecuadoriana* Erwin & Zamorano, sp. n. ADP132468, Limoncocha, Ecuador. Endophallus everted. Scale line = 0.25 mm.

**Figure 69–74.**
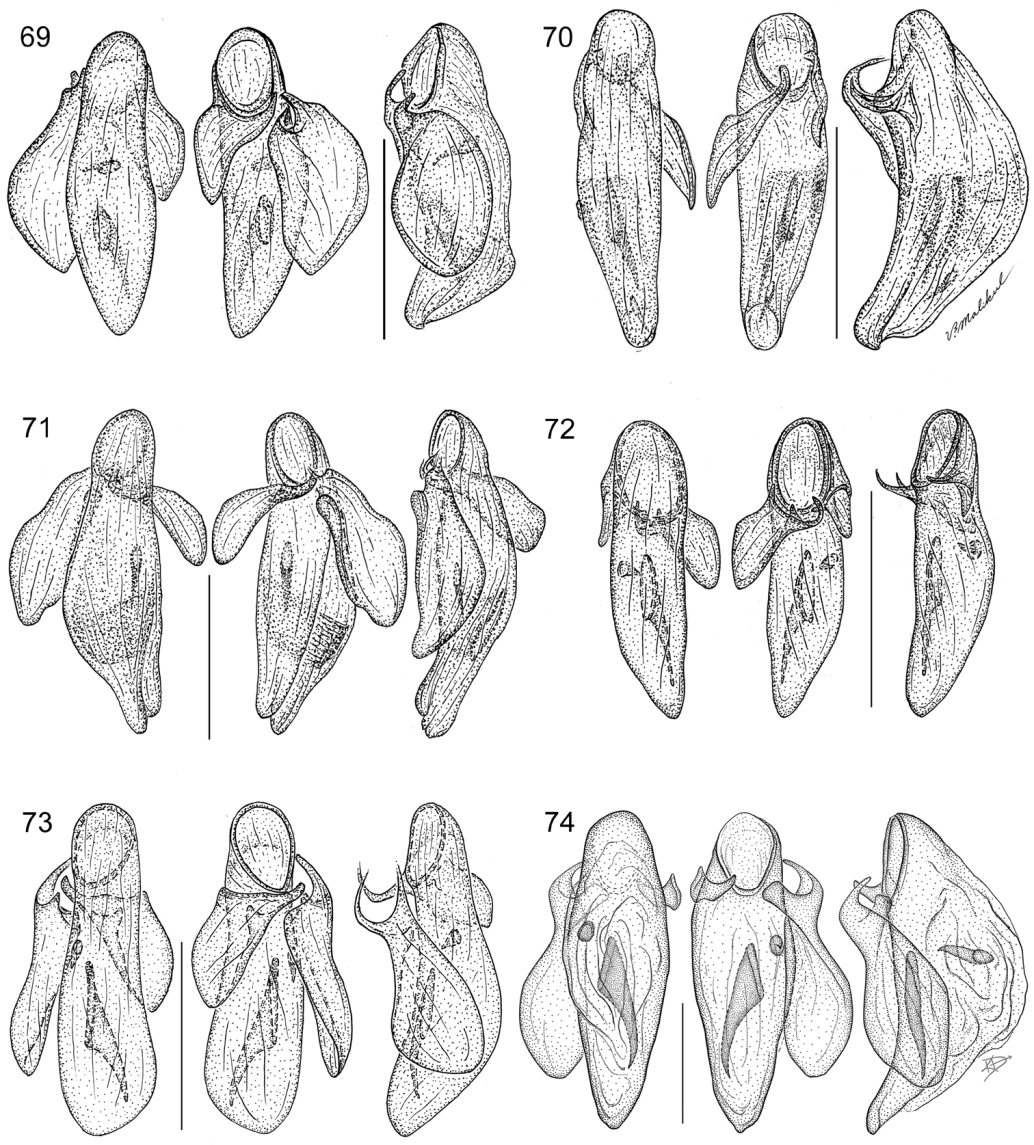
**69** *Asklepia kathleenae* Erwin & Zamorano, sp. n. ADP132529, Belém, Brazil **70** *Asklepia marchantaria* Erwin & Zamorano, sp. n. Adis # 001103, Ilha de Marchantaria, Lago Camaleão, Brazil **71** *Asklepia pakitza* Erwin & Zamorano, sp. n. ADP132466, Pakitza, Perú **72** *Asklepia paraguayensis* Zamorano & Erwin, sp. n. ADP132769, San Lorenzo Paraguay **73** *Asklepia surinamensis* Zamorano & Erwin, sp. n. ADP132763, l’Hermitage, Surinam **74** *Asklepia vigilante* Erwin & Zamorano, sp. n. ADP067301, Boca del Río Samiria, Perú.

**Figure 75.**
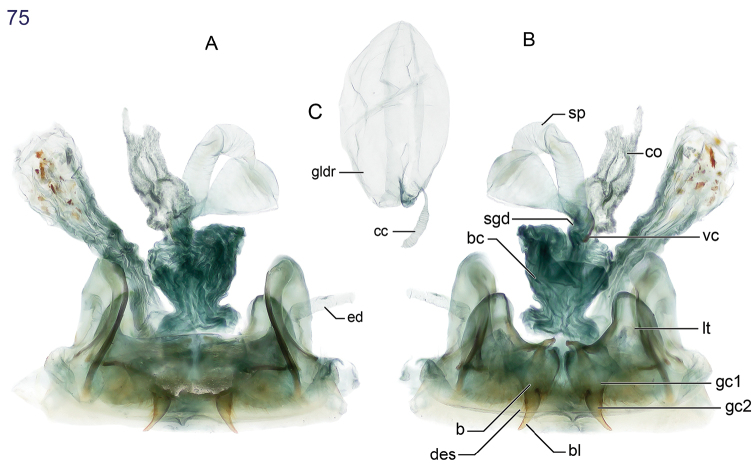
*Asklepia demiti* sp. n. Digital Photo-illustration, female genitalia based on specimen ADP132483 from Rio Demiti, Brazil. **A** Dorsal aspect. Legend, **bc**, bursa copulatrix; **co** common oviduct; **sg** spermathecal gland; **sgd** spermathecal gland duct; **sp** spermatheca. dorsal aspect; **vc** villous canal; **lt** laterotergite; **gc1** gonocoxite 1; **gc2** gonocoxite 2. **B** Gonocoxite 2, dorsal aspect: Legend, **b** base of gonocoxite 2; **bl** blade of gonocoxite 2; **des** dorsal ensiform seta. C. Defense gland (**gldr**); **cc** accessory gland; **ed** efferent duct.

**Figure 76–77.**
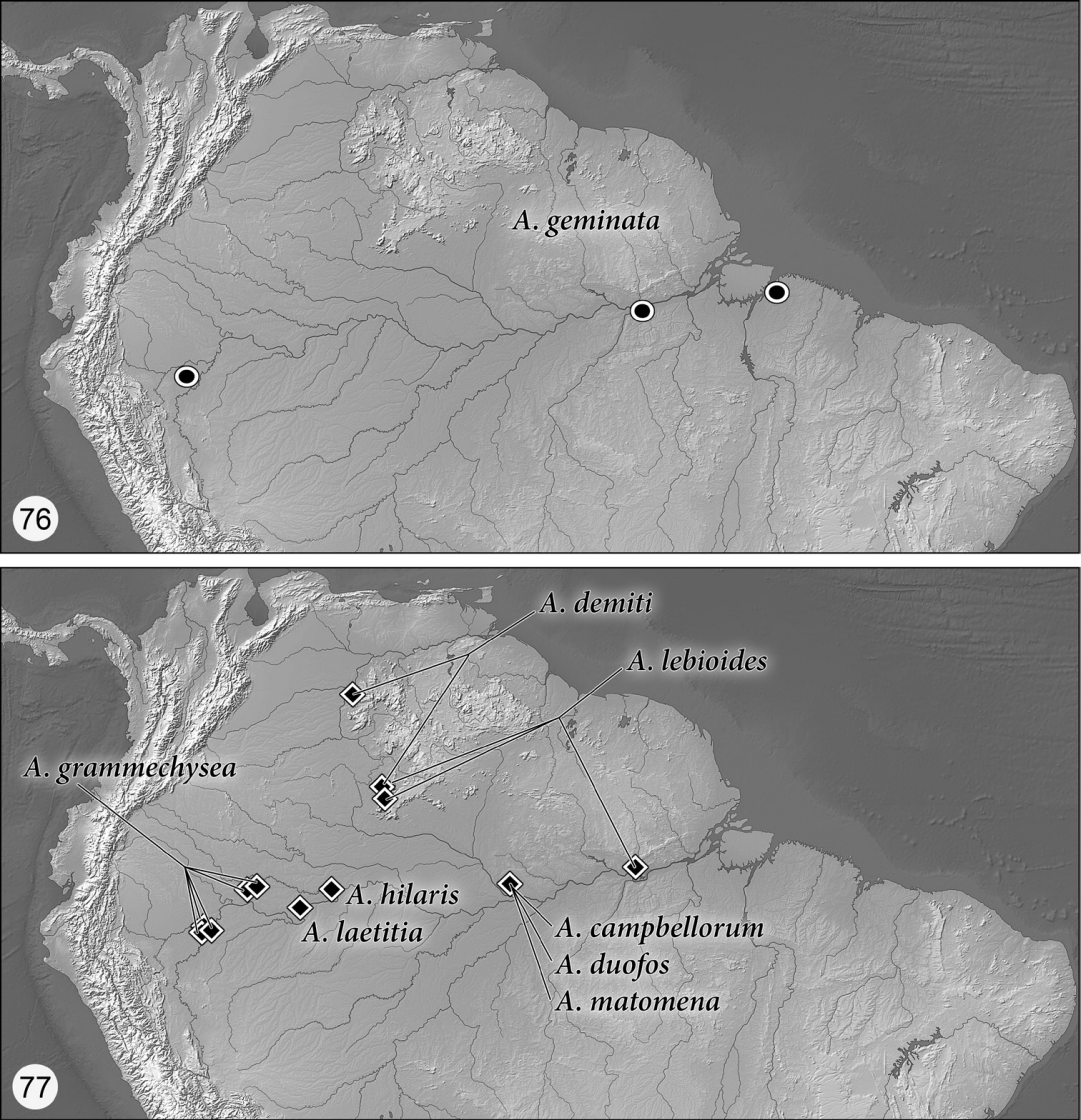
**76** Distribution map for known localities of *Asklepia geminata* (Bates) **77** Distribution map for known localities of *Asklepia* species of the *hilaris* group.

**Figure 78.**
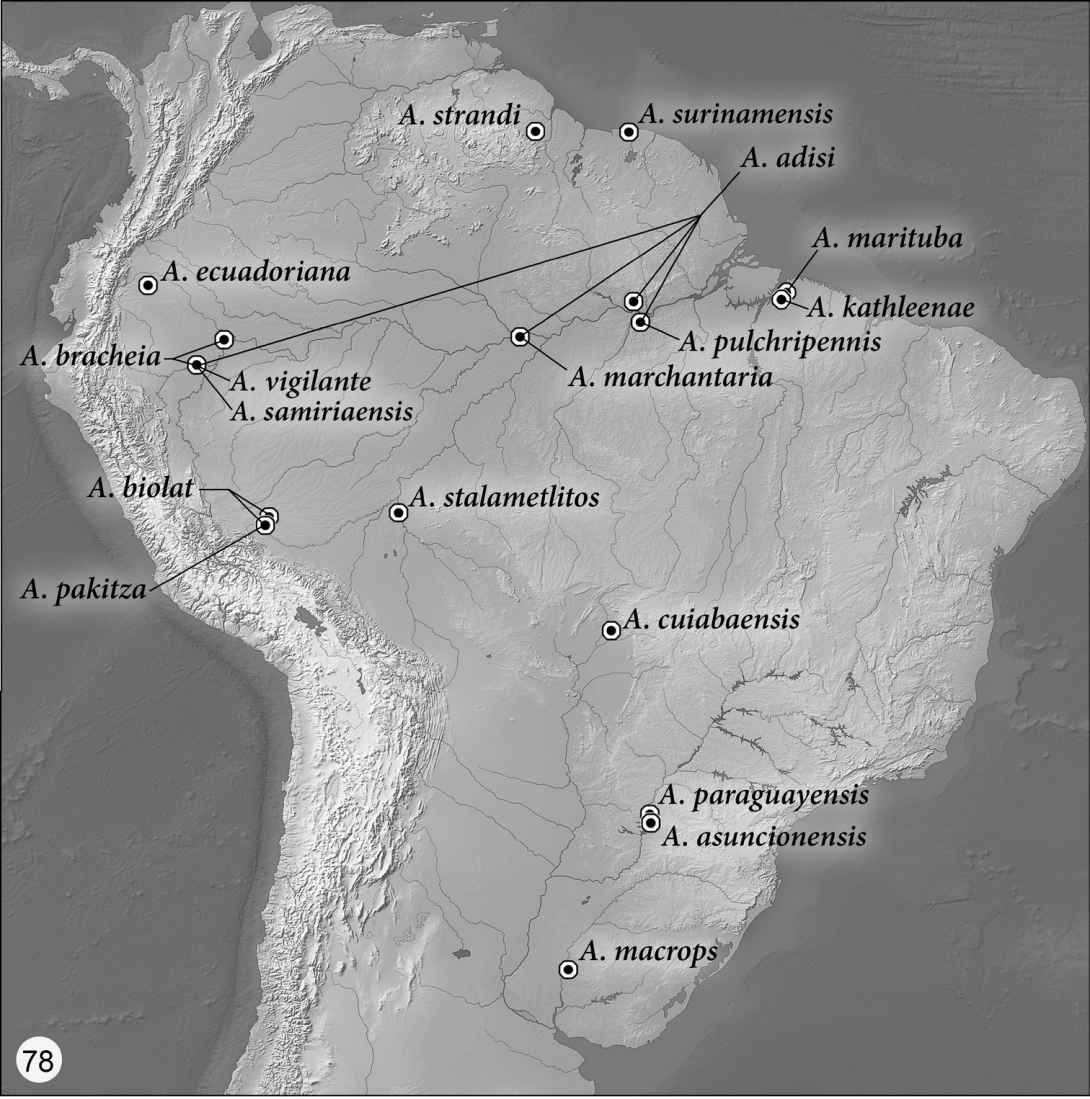
Distribution map for known localities of *Asklepia* species of the *pulchripennis* group.

**Figure 79.**
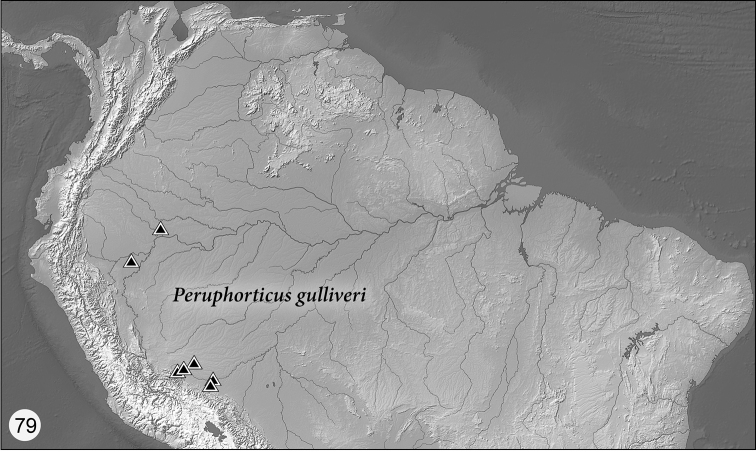
Distribution map for known localities of *Peruphorticus gulliveri* sp. n.

## Summary and future directions

The lachnophorine assemblage is species rich as evidenced by vast collections in museums. Adults are diverse and divergent in physical attributes ranging from the agonine-like *Anchonoderus* and *Homethes* to the rather elegant and bizarre ant-like *Ega*, *Selina*, and *Stenocheila*. In color attributes, the adults range from some charcoal black *Anchonoderus* to those of the incredibly beautiful, multi-hued *Quammenis spectabilis* (Fig. 1). In spite of this strikingly broad, readily perceived range of attributes that are pleasing to the eye, and challenging to the mind of evolutionary biologists, revisions, or monographs for the Tribe, or its component genera, surprisingly are lacking. The literature contains many single species descriptions in several of the genera, mostly from the 19^th^ century – the golden age of exploration and descriptive entomology. Ball (1960) provided keys and notes on the North American taxa and later (Ball and Hilchie 1983) recognized the “Eucaerine complex” as Lachnophorini rather than Lebiini. Reichardt (1977) provided a key and notes on the Neotropical genera known to him. These comparatively recent regional treatments of Carabidae include cursory to detailed reference to lachnophorine taxa, featuring the generic level, using adult attributes. Liebherr (1983) described the larva of *Ega sallei* Chevrolat and that is the only described larva of the entire Tribe Lachnophorini; however, the junior author (LSZ) has specimens of two additional genera that she will include in her Master’s studies. Liebherr (1988) also provided a phylogenetic treatment of Lachnophorini of the Caribbean Islands and aligned the tribe with the Odacanthini. More recently, the senior author (TLE) provided descriptions of two new genera (Erwin 2000, 2004) and keys to most lachnophorine genera (Erwin et al. 2002; Erwin et al. 2012) in the Western Hemisphere. Here, we add two additional genera found in Brazil, and three genera from the Eastern Hemisphere.

That information, noted above, was the platform available to us for beginning construction of a robust understanding of the Lachnophorini to at least a level represented by many Northern Hemisphere tribes of Carabidae. So that others may join in this potentially exciting pursuit, we have offered an illustrated preliminary taxonomic synopsis of the known lachnophorine genera, with keys, and a species-level treatment of the taxonomically complex *Asklepia* Liebke.

During this study, it became readily apparent that the tropics, in both New and Old world, are the center of origin and radiation of taxa. A very few species occur in the southern parts of the United States, likewise south only to mid Argentina. Relationships between New and Old world taxa need to be understood and that will only come with a well-founded understanding of the phylogeny. With regard to the species of *Asklepia*, it is quite obvious that the three species groups (defined so well by attributes of the endophallus and pronotal margins) have co-evolved in an Amazon center, probably tied in some way to Varzea and Igapó waters as habitats for differentiation of species groups; however, we do not have sufficient samples of most species to test this idea. Some species occur south as far as Argentina, but none have made it to Middle America; all are cis-Andean. Additionally, thanks to an observation made by George E. Ball (pers. comm.), it seems that the least derived species groups, *geminatus* (typical carabid pronotum with lateral explanations, convex elytral intervals, and no spines on the endophallus) and *hilaris* (typical carabid pronotum with lateral explanations and multiple small spines on the endophallus) are confined to the central Amazon River drainage, while the most derived group *pulchripennis* (lacking pronotal explanations and having two large spines on the endophallus) is not confined and has member species occurring as far south as Argentina and Uruguay, as well as on extreme tributaries of the Amazon drainage (see Figs 76–78). We ponder whether this is indicative of a flatland continental taxon cycle and suggest that with better collections, this should be tested.

The path forward is branched. For the near future, the branch to be followed by one of us, (LSZ), will lead to an attempt, using morphological and molecular attributes, to reconstruct the phylogeny of the known lachnophorine genera, with the expectation that the inferred system of relationships will provide a firm basis for a stable classification. In turn, this classification will provide a rational basis for exploration of diversity and divergence within each of the lachnophorine genera, the second branch.

## Supplementary Material

XML Treatment for Lachnophorini

XML Treatment for Aeolodermus

XML Treatment for Amphithasus

XML Treatment for Anchonoderus

XML Treatment for Aporesthus

XML Treatment for Calybe

XML Treatment for Diplacanthogaster

XML Treatment for Ega

XML Treatment for Eucaerus

XML Treatment for Euphorticus

XML Treatment for Guatemalteca

XML Treatment for Homethes

XML Treatment for Lachnaces

XML Treatment for Lachnophorus

XML Treatment for Peruphorticus

XML Treatment for Peruphorticus gulliveri

XML Treatment for Pseudophorticus

XML Treatment for Quammenis

XML Treatment for Selina

XML Treatment for Stenocheila

XML Treatment for Asklepia

XML Treatment for Asklepia geminata

XML Treatment for Asklepia campbellorum

XML Treatment for Asklepia demiti

XML Treatment for Asklepia duofos

XML Treatment for Asklepia grammechrysea

XML Treatment for Asklepia hilaris

XML Treatment for Asklepia laetitia

XML Treatment for Asklepia lebioides

XML Treatment for Asklepia matomena

XML Treatment for Asklepia adisi

XML Treatment for Asklepia asuncionensis

XML Treatment for Asklepia biolat

XML Treatment for Asklepia bracheia

XML Treatment for Asklepia cuiabaensis

XML Treatment for Asklepia ecuadoriana

XML Treatment for Asklepia kathleenae

XML Treatment for Asklepia macrops

XML Treatment for Asklepia marchantaria

XML Treatment for Asklepia marituba

XML Treatment for Asklepia pakitza

XML Treatment for Asklepia paraguayensis

XML Treatment for Asklepia pulchripennis

XML Treatment for Asklepia samiriaensis

XML Treatment for Asklepia stalametlitos

XML Treatment for Asklepia strandi

XML Treatment for Asklepia surinamensis

XML Treatment for Asklepia vigilante

## Appendix 1

Morphological measurements and ratios for adults of species of *Asklepia* Liebke, 1938. All values are in millimeters. Apparent body length (ABL) is provided in the descriptions. Means provided for ratios are “harmonic means.”

### *geminata* species group

**Table 1.** *Asklepia geminata* (Bates, 1871)

**Standard deviation**							
**SBL**	**MW**	**LP**	**LE**	**LH**	**WP**	**WH**	**WE**
0.199	0.021	0.009	0.048	0.161	0.033	0.061	0.010
**Total length**							
Specimens	N	Range		Mean			
	2	2.512–2.794		2.653			
**Total length**							
Specimens	N	Range		Mean			
	2	1.411–1.440		1.426			
**Width of head/Total width**							
Specimens	N	Range		Mean			
	2	0.513–0.536		0.524			
**Pronotum: Width (at widest part)/Length**							
Specimens	N	Range		Mean			
	2	1.359–1.368		1.364			
**Length of pronotum / Length of head**							
Specimens	N	Range		Mean			
	6	1.514–1.768		1.631			

### *hilaris* species group

**Table 2.** *Asklepia campbellorum* Zamorano & Erwin, sp. n.

**Standard deviation**							
**SBL**	**MW**	**LP**	**LE**	**LH**	**WP**	**WH**	**WE**
0.282	0.174	0.041	0.060	0.190	0.073	0.079	0.087
**Total length**							
Specimens	N	Range		Mean			
	25	2.182–3.199		2.254			
**Total width**							
Specimens	N	Range		Mean			
	25	1.107–1.824		1.615			
**Width of head/Total width**							
Specimens	N	Range		Mean			
	25	0.458–0.547		0.491			
**Pronotum: width (at widest part)/length**							
Specimens	N	Range		Mean			
	25	1.294–1.498		1.372			
**Length of pronotum / Length of head**							
Specimens	N	Range		Mean			
	25	1.265–1.590		1.411			

**Table 3.** *Asklepia demiti* Erwin & Zamorano, sp. n.

**Standard deviation**							
**SBL**	**MW**	**LP**	**LE**	**LH**	**WP**	**WH**	**WE**
0.135	0.092	0.053	0.028	0.086	0.027	0.029	0.046
**Total length**							
Specimens	N	Range		Mean			
	17	2.590–3.131		2.953			
**Total length**							
Specimens	N	Range		Mean			
	17	1.491–1.815		1.678			
**Width of head/Total width**							
Specimens	N	Range		Mean			
	17	0.459–0.525		0.491			
**Pronotum: Width (at widest part)/Length**							
Specimens	N	Range		Mean			
	17	1.331–1.550		1.380			
**Length of pronotum / Length of head**							
Specimens	N	Range		Mean			
	17	1.185–1.928		1.446			

**Table 4.** *Asklepia duofos *Zamorano & Erwin, sp. n.

N	**SBL**	**MW**	**LH**	**LP**	**LE**	**WH**	**WP**	**WE**
1	2.941	1.592	0.417	0.593	1.931	0.779	0.829	0.796
	**WH/TW**	**PW/PL**	**LP/LH**	**WP/TW**	**WH/WP**			
	0.489	1.397	1.423	0.521	0.939			

**Table 5.** *Asklepia grammechrysea* Zamorano & Erwin, sp. n.

**Standard deviation**							
**SBL**	**MW**	**LP**	**LE**	**LH**	**WP**	**WH**	**WE**
0.128	0.260	0.071	0.103	0.126	0.115	0.012	0.163
**Total length**							
Specimens	N	Range		Mean			
	33	2.265–3.736		3.095			
**Total length**							
Specimens	N	Range		Mean			
	33	1.382–2.216		1.811			
**Width of head/Total width**							
Specimens	N	Range		Mean			
	33	0.430–0.491		0.481			
**Pronotum: Width (at widest part)/Length**							
Specimens	N	Range		Mean			
	33	1.273–2.228		1.381			
**Length of pronotum / Length of head**							
Specimens	N	Range		Mean			
	33	1.273–2.228		1.512			

**Table 6.** *Asklepia hilaris* (Bates, 1871), comb. n.

N	**SBL**	**MW**	**LH**	**LP**	**LE**	**WH**	**WP**	**WE**
1	2.336	1.233	0.359	0.483	1.495	0.678	0.625	0.617
	**WH/TW**	**PW/PL**	**LP/LH**	**WP/TW**	**WH/WP**			
	1.099	1.294	1.346	1.013	1.085			

**Table 7.** *Asklepia laetitia* Zamorano & Erwin, sp. n.

N	**SBL**	**MW**	**ABL**	**LH**	**LP**	**LE**	**WH**	**WP**	**WE**
1	2.494	1.463	2.990	0.372	0.489	1.633	0.684	0.706	0.731
	**WH/TW**	**WP/LP**	**LP/LH**	**WP/TW**	**WP/WH**				
	0.468	1.444	1.316	0.483	1.032				

**Table 8.** *Asklepia lebioides* (Bates, 1871), comb. n.

**Standard deviation**							
**SBL**	**MW**	**LP**	**LE**	**LH**	**WP**	**WH**	**WE**
0.108	0.088	0.019	0.021	0.082	0.029	0.030	0.044
**Total length**							
Specimens	N	Range		Mean			
	17	2.692–3.142		2.991			
**Total length**							
Specimens	N	Range		Mean			
	17	1.504–1.790		1.666			
**Width of head/Total width**							
Specimens	N	Range		Mean			
	17	0.460–0.547		0.487			
**Pronotum: Width (at widest part)/Length**							
Specimens	N	Range		Mean			
	17	1.686–1.960		1.824			
**Length of pronotum / Length of head**							
Specimens	N	Range		Mean			
	17	1.244–1.430		1.349			

**Table 9.** *Asklepia matomena* Zamorano & Erwin, sp. n.

**Standard deviation**							
**SBL**	**MW**	**LP**	**LE**	**LH**	**WP**	**WH**	**WE**
0.343	0.256	0.213	0.036	0.070	0.149	0.062	0.090
**Total length**							
Specimens	N	Range		Mean			
	2	2.189–2.551		2.370			
**Total length**							
Specimens	N	Range		Mean			
	2	1.150–1.452		1.301			
**Width of head/Total width**							
Specimens	N	Range		Mean			
	2	0.493–0.546		0.518			
**Pronotum: Width (at widest part)/Length**							
Specimens	N	Range		Mean			
	2	1.338–1.351		1.345			
**Length of pronotum / Length of head**							
Specimens	N	Range		Mean			
	2	1.348–1.425		1.385			

### *pulchripennis* species group

(Note: There are no measures for *Asklepia strandi* Liebke except ABL).

**Table 10.** *Asklepia adisi* Erwin & Zamorano, sp. n.

**Standard deviation**							
**SBL**	**MW**	**LP**	**LE**	**LH**	**WP**	**WH**	**WE**
0.055	0.045	0.016	0.028	0.042	0.017	0.021	0.022
**Total length**							
Specimens	N	Range		Mean			
	7	2.542–2.688		2.632			
**Total length**							
Specimens	N	Range		Mean			
	7	1.341–1.473		1.412			
**Width of head/Total width**							
Specimens	N	Range		Mean			
	7	0.517–0.573		0.535			
**Pronotum: Width (at widest part)/Length**							
Specimens	N	Range		Mean			
	7	1.154–1.314		1.226			
**Length of pronotum / Length of head**							
Specimens	N	Range		Mean			
	7	1.221–1.464		1.327			

**Table 11.** *Asklepia asuncionensis* Erwin & Zamorano, sp. n.

N	**SBL**	**TW**	**LH**	**LP**	**LE**	**WH**	**WP**	**WE**
1	2.477	1.27	0.378	0.533	1.566	0.671	0.648	0.635
	**WH/TW**	**WP/LP**	**LP/LH**	**WP/TW**	**WH/WP**			
	0.528	1.714	1.191	0.337	2.555			

**Table 12.** *Asklepia biolat* Erwin & Zamorano, sp. n.

**Standard Deviation**							
**SBL**	**MW**	**LP**	**LE**	**LH**	**WP**	**WH**	**WE**
0.123	0.103	0.060	0.028	0.074	0.034	0.041	0.051
**Total length**							
Specimens	N	Range		Mean			
	24	2.787–3.247		3.014			
**Total length**							
Specimens	N	Range		Mean			
	24	1.388–1.816		1.633			
**Width of head/Total width**							
Specimens	N	Range		Mean			
	24	0.490–0.594		0.522			
**Pronotum: Width (at widest part)/Length**							
Specimens	N	Range		Mean			
	24	1.141–1.322		1.244			
**Length of pronotum / Length of head**							
Specimens	N	Range		Mean			
	24	1.398–2.162		1.603			

**Table 13.** *Asklepia bracheia* Zamorano & Erwin, sp. n.

**Standard deviation**							
**SBL**	**MW**	**LP**	**LE**	**LH**	**WP**	**WH**	**WE**
0.113	0.059	0.037	0.035	0.051	0.034	0.048	0.030
**Total length**							
Specimens	N	Range		Mean			
	8	1.952–2.345		2.150			
**Total length**							
Specimens	N	Range		Mean			
	8	1.074–1.274		1.197			
**Width of head/Total width**							
Specimens	N	Range		Mean			
	8	0.502–0.584		0.529			
**Pronotum: Width (at widest part)/Length**							
Specimens	N	Range		Mean			
	8	1.000–1.304		1.205			
**Length of pronotum / Length of head**							
Specimens	N	Range		Mean			
	8	1.359–2.050		1.590			

**Table 14.** *Asklepia cuiabaensis* Erwin & Zamorano, sp. n.

**SBL**	**TW**	**LH**	**LP**	**LE**	**WH**	**WP**	**WE**
2.191	1.27	0.343	0.476	1.372	0.629	0.592	0.635
	**WH/TW**	**WP/LP**	**LP/LH**	**WP/TW**	**WH/WP**		
	0.495	1.726	1.334	0.361	2.744		

**Table 15.** *Asklepia ecuadoriana* Erwin & Zamorano, sp. n.

**Standard deviation**							
**SBL**	**MW**	**LP**	**LE**	**LH**	**WP**	**WH**	**WE**
0.135	0.092	0.053	0.028	0.086	0.027	0.029	0.046
**Total length**							
Specimens	N	Range		Mean			
	17	2.590- 3.131		2.953			
**Total length**							
Specimens	N	Range		Mean			
	17	1.491–1.815		1.678			
**Width of head/Total width**							
Specimens	N	Range		Mean			
	17	0.459–0.525		0.491			
**Pronotum: Width (at widest part)/Length**							
Specimens	N	Range		Mean			
	17	1.331–1.550		1.380			
**Length of pronotum / Length of head**							
Specimens	N	Range		Mean			
	17	1.185–1.928		1.446			

**Table 16.** *Asklepia kathleenae* Erwin & Zamorano, sp. n.

**SBL**	**MW**	**LH**	**LP**	**LE**	**WH**	**WP**	**WE**
2.167	1.241	0.342	0.484	1.341	0.646	0.569	0.620
	**WH/TW**	**WP/LP**	**LP/LH**	**WP/TW**	**WH/WP**	**LE/WE**	
	0.520	1.176	1.414	0.459	1.135	2.162	

**Table 17.** *Asklepia macrops* Erwin & Zamorano, sp. n.

N	**SBL**	**MW**	**LH**	**LP**	**LE**	**WH**	**WP**	**WE**
1	3.054	1.648	0.490	0.621	1.942	0.871	0.779	0.824
	**WH/TW**	**WP/LP**	**LP/LH**	**WP/TW**	**WH/WP**			
	0.529	1.254	1.267	0.472	1.119			

**Table 18.** *Asklepia marchantaria* Erwin & Zamorano, sp. n.

**Standard deviation**							
**SBL**	**MW**	**LP**	**LE**	**LH**	**WP**	**WH**	**WE**
0.077	0.109	0.032	0.027	0.067	0.028	0.018	0.055
**Total length**							
Specimens	N	Range		Mean			
	7	2.599–2.828		2.675			
**Total length**							
Specimens	N	Range		Mean			
	7	1.324–1.582		1.457			
**Width of head/Total width**							
Specimens	N	Range		Mean			
	7	0.500–0.578		0.529			
**Pronotum: Width (at widest part)/Length**							
Specimens	N	Range		Mean			
	7	1.108–1.238		1.185			
**Length of pronotum / Length of head**							
Specimens	N	Range		Mean			
	7	1.114–1.523		1.382			

**Table 19.** *Asklepia marituba* Zamorano & Erwin, sp. n.

**SBL**	**MW**	**ABL**	**HL**	**PL**	**EL**	**WH**	**WP**	**WE**
2.732	1.574	2.924	0.433	0.605	1.694	0.820	0.768	0.787
	**WH/TW**	**WP/LP**	**PL/LH**	**WP/TW**	**WH/WP**			
	0.521	1.268	1.398	0.488	1.069			

**Table 20.** *Asklepia pakitza* Erwin & Zamorano, sp. n.

**Standard deviation**							
**SBL**	**MW**	**LP**	**LE**	**LH**	**WP**	**WH**	**WE**
0.033	0.022	0.057	0.006	0.030	0.007	0.017	0.011
**Total length**							
Specimens	N	Range		Mean			
	2	2.635–2.683		2.659			
**Total length**							
Specimens	N	Range		Mean			
	2	1.399–1.430		1.414			
**Width of head/Total width**							
Specimens	N	Range		Mean			
	6	0.540–0.545		0.542			
**Pronotum: Width (at widest part)/Length**							
Specimens	N	Range		Mean			
	6	1.308–1.370		1.338			
**Length of pronotum / Length of head**							
Specimens	N	Range		Mean			
	6	1.39–1.713		1.538			

**Table 21.** *Asklepia paraguayensis* Zamorano & Erwin, sp. n.

**Standard deviation**							
**SBL**	**MW**	**LP**	**LE**	**LH**	**WP**	**WH**	**WE**
0.135	0.105	0.019	0.027	0.094	0.049	0.048	0.053
**Total length**							
Specimens	N	Range		Mean			
	4	2.478–2.769		2.574			
**Total length**							
Specimens	N	Range		Mean			
	4	1.264–1.506		1.362			
**Width of head/Total width**							
Specimens	N	Range		Mean			
	4	0.517–0.544		0.527			
**Pronotum: Width (at widest part)/Length**							
					
Specimens	N	Range		Mean			
	4	1.617–1.779		1.693			
**Length of pronotum / Length of head**							
Specimens	N	Range		Mean			
	4	1.365–1.447		1.397			

**Table 22.** *Asklepia pulchripennis* (Bates, 1871), comb. n.

**SBL**	**MW**	**LH**	**LP**	**LE**	**WH**	**WP**	**WE**
2.718	1.366	0.402	0.584	1.732	0.769	0.700	0.683
	**WH/TW**	**WP/LP**	**LP / LH**	**WP/TW**	**WH/WP**		
	0.563	1.198	1.454	0.512	1.099		

**Table 23.** *Asklepia samiriaensis* Zamorano & Erwin, sp. n.

**SBL**	**MW**	**LH**	**LP**	**LE**	**WH**	**WP**	**WE**
2.436	1.274	0.396	0.537	1.503	0.728	0.613	0.637
	**WH/TW**	**WP/LP**	**LP/LH**	**WP/LP**	**WP/TW**	**WH/WP**	
	0.571	1.141	1.357	0.962	0.481	1.188	

**Table 24.** *Asklepia stalametlitos* Zamorano & Erwin, sp. n.

**SBL**	**MW**	**LH**	**LP**	**LE**	**WH**	**WP**	**WE**
2.815	1.467	0.430	0.597	1.788	0.818	0.751	0.733
	**WH/TW**	**WP/LP**	**LP/LH**	**WP/LP**	**WP/TW**	**WH/WP**	
	0.557	1.258	1.388	1.024	0.512	1.089	

**Table 25.** *Asklepia surinamensis* Zamorano & Erwin, sp. n.

**Standard deviation**							
**SBL**	**MW**	**LP**	**LE**	**LH**	**WP**	**WH**	**WE**
0.156	0.138	0.036	0.029	0.098	0.040	0.046	0.069
**Total length**							
Specimens	N	Range		Mean			
	6	2.541–2.992		2.843			
**Total length**							
Specimens	N	Range		Mean			
	6	1.214–1.584		1.459			
**Width of head/Total width**							
Specimens	N	Range		Mean			
	6	0.518–0.599		0.551			
**Pronotum: Width (at widest part)/Length**							
Specimens	N	Range		Mean			
	6	1.340–1.385		1.369			
**Length of pronotum / Length of head**							
Specimens	N	Range		Mean			
	6	1.042–1.169		1.105			

**Table 26.** *Asklepia vigilante* Erwin & Zamorano, sp. n.

**Standard deviation**							
**SBL**	**MW**	**LP**	**LE**	**LH**	**WP**	**WH**	**WE**
0.277	0.081	0.065	0.182	0.040	0.061	0.063	0.040
**Total length**							
Specimens	N	Range		Mean			
	6	2.589–3.259		2.867			
**Total length**							
Specimens	N	Range		Mean			
	6	1.397–1.598		1.506			
**Width of head/Total width**							
Specimens	N	Range		Mean			
	6	0.505-0.565		0.508			
**Pronotum: Width (at widest part)/Length**							
Specimens	N	Range		Mean			
	6	1.146–1.265		1.209			
**Length of pronotum / Length of head**							
Specimens	N	Range		Mean			
	6	1.307–1.552		1.436			

### Appendix 2

**Table:** Table with measures and ratios for adults of species of *Peruphorticus gulliveri* Erwin & Zamorano gen. n., sp. n. All measures are in millimeters. Apparent body length (ABL) is provided in the description. Means provided are “harmonic means.”

**Standard deviation**							
**SBL**	**MW**	**LP**	**LE**	**LH**	**WP**	**WH**	**WE**
0.245	0.274	0.038	0.059	0.196	0.045	0.074	0.137
**Total length**							
Specimens	N	Range	Mean				
	36	5.281–6.146	5.803				
**Total length**							
Specimens	N	Range	Mean				
	36	2.3–3.6	2.685				
**Width of head/Total width**							
Specimens	N	Range	Mean				
	36	0.366–0.533	0.484				
**Pronotum: Width (at widest part)/Length**							
Specimens	N	Range	Mean				
	36	1.805–2.377	2.085				
**Length of pronotum / Length of head**							
Specimens	N	Range	Mean				
	36	1.340–1.757	1.496				
